# Angiogenic factor-driven inflammation promotes extravasation of human proangiogenic monocytes to tumours

**DOI:** 10.1038/s41467-017-02610-0

**Published:** 2018-01-24

**Authors:** Adama Sidibe, Patricia Ropraz, Stéphane Jemelin, Yalin Emre, Marine Poittevin, Marc Pocard, Paul F. Bradfield, Beat A. Imhof

**Affiliations:** 1 Department of Pathology and Immunology, Centre Médical Universitaire (CMU), Medical faculty, University of Geneva, Rue Michel-Servet 1, CH-1211 Geneva, Switzerland; 2 Department of Oncologic and Digestive Surgery, AP-HP, Hospital Lariboisière, 2 rue Ambroise Paré, F-75475 Paris cedex 10, France; 3 Université Paris Diderot, Sorbonne Paris Cité, CART, INSERM U965, 49 boulevard de la Chapelle, F-75475 Paris cedex 10, France; 4 Present Address: Department of Physiology and Metabolism, Centre Médical Universitaire (CMU), Medical faculty, University of Geneva, Rue Michel-Servet 1, CH-1211 Geneva, Switzerland

## Abstract

Recruitment of circulating monocytes is critical for tumour angiogenesis. However, how human monocyte subpopulations extravasate to tumours is unclear. Here we show mechanisms of extravasation of human CD14^dim^CD16^+^ patrolling and CD14^+^CD16^+^ intermediate proangiogenic monocytes (HPMo), using human tumour xenograft models and live imaging of transmigration. IFNγ promotes an increase of the chemokine CX3CL1 on vessel lumen, imposing continuous crawling to HPMo and making these monocytes insensitive to chemokines required for their extravasation. Expression of the angiogenic factor VEGF and the inflammatory cytokine TNF by tumour cells enables HPMo extravasation by inducing GATA3-mediated repression of CX3CL1 expression. Recruited HPMo boosts angiogenesis by secreting MMP9 leading to release of matrix-bound VEGF-A, which amplifies the entry of more HPMo into tumours. Uncovering the extravasation cascade of HPMo sets the stage for future tumour therapies.

## Abstract (web summary)

Circulating myeloid cells can leave the vasculature to infiltrate tumours and are thought to contribute to tumour angiogenesis. Here the authors live image monocytes that migrate to xenograft tumours and map an extravasation cascade of human proangiogenic monocytes into the tumour.

## Introduction

Targeting tumour growth is a successful strategy in treating cancer. However, tumour resistance that develops along the course of disease continues to be a fundamental challenge for long-term treatment strategies^1^. In mice, a population of CD11b^+^Gr1^+^ monocytes and their macrophage progeny have a central function in tumour resistance to VEGF-based anti-angiogenic therapy, suppression of anti-tumour immunity and metastasis^2^. In addition, the alternatively activated phenotypes of tumour-associated macrophages (TAM) promote the secretion of angiogenic factors in tumour hypoxic areas, leading to tumour neovascularization^3^. The plasticity of macrophages with an outcome of tumour suppression or tumour growth highlights the challenges in targeting these cells in cancer therapies^4^. Defining human monocyte subsets with proangiogenic and protumorigenic functions, as well as understanding their extravasation cascade would present opportunity for rapid clinical translation.

The monocyte pool consists of different subsets with a diversity of specific functions in a variety of processes^5,6^. In mice, a circulating monocyte subset expressing angiopoietin receptor Tie2 exerts proangiogenic and immune suppressor functions on solid tumours^5,7^. Other subsets, including CD11b^+^Gr1^+^ myeloid cells and CD11b^+^Vegfr1^+^ myeloid-derived suppressor cells (MDSC), might also sustain tumour angiogenesis^2,8^. Further studies in mice have identified two distinct monocyte populations in blood, namely GR1^+^ inflammatory and GR1^−^ patrolling monocytes, both endowed with specific inflammatory functions^9–11^. However, the question remains whether all murine proangiogenic monocytes belong to the same GR1^+^ subset^12,13^. Historically, functional differences between mouse and human monocytes has impeded the identification of subsets with evolutionarily conserved angiogenic functions. In humans, the pan-monocyte population is comprised of inflammatory CD14^+^CD16^−^, intermediate CD14^+^CD16^+^ and patrolling CD14^dim^CD16^+^ cells also with ascribed specific inflammatory functions^6^. In contrast to mouse, human TIE2^+^ monocytes have been reported to belong to CD16^+^ subsets and to elicit proangiogenic and protumorigenic activity in solid tumours in vivo^14,15^. Understanding the extended migration cascade of human angiogenic monocytes may identify therapeutic targets for human tumour treatment and allow rapid translation to the clinic.

Here we study the trafficking profiles of human angiogenic monocyte subsets to solid tumours. We define a new set of inflammatory conditions for which we further explore the mechanisms of action of proangiogenic monocyte recruitment to tumours, as well as their effects on tumour growth.

## Results

### Different homing of human proangiogenic monocytes to tumours

Previous studies suggested species differences in the definition of angiogenic subsets of monocytes, which was reported to be inflammatory GR1+ in mouse and non-classical CD16+ in human^8,11,14,15^. Human monocytes are defined as CD19^−^CD3^−^HLA-DR^+^CD300e^+^CD56^−^CD115^+^CD141^−^ cells (Supplementary Figure 1a). Among these we confirm classical inflammatory and the TIE2 high expressing, non-classical CD14^dim^CD16^+^ and TIE2 intermediately expressing CD14^+^CD16^+^ monocytes (Supplementary Note 1, Supplementary Figure 1b, c)^5,15,16^. The non-classical monocytes strongly promoted endothelial cell proliferation and tube formation, the intermediate cells were less active, while inflammatory monocytes showed no activity (Supplementary Note 2, Supplementary Figures 2, 3). Concordantly, the pool of CD16^+^ monocytes boosted angiogenesis in subcutaneous (s.c.) xenografts of human colorectal carcinoma cells (DLD1 and HCT116) in NOD/SCID mice when monocytes were co-injected along with the tumour cells (Supplementary Note 3, Supplementary Figure 4). We refer in this study to CD16^+^ monocytes collectively as human proangiogenic monocytes (HPMo) and to CD14^+^CD16^−^ inflammatory cells as human non-angiogenic monocytes (HNMo). However, HPMo did not affect angiogenesis and growth of breast tumour cell (SKBR7) xenografts indicating that in certain tumours the angiogenic function of HPMo is neutralised (Supplementary Figure 4b–d).

**Fig. 1:**
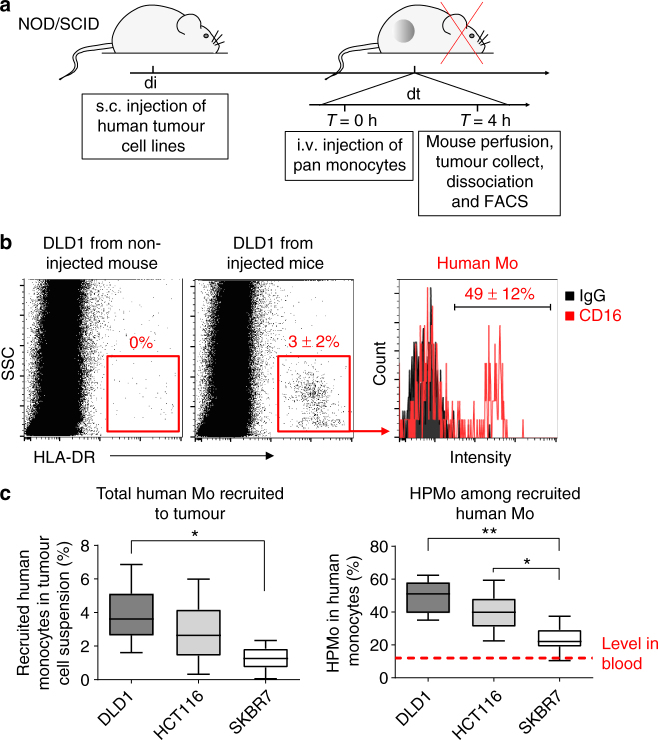
Human tumour xenografts recruit human proangiogenic monocytes. **a** Time course protocol of the assay of human monocyte recruitment to human tumour xenograft (colorectal adenocarcinoma DLD1, colorectal carcinoma HCT116 and breast carcinoma SKBR7) in vivo. Human tumour xenografts were injected subcutaneously at day di and let grow to reach 0.5 cm^3^ of tumour volume. The mice were then i.v. injected at day dt with freshly isolated human monocytes. After 4 h mice were perfused with PBS-EDTA and killed to collect tumours that were then dissociated for FACS analysis of recruited human monocytes. **b** FACS analysis of recruited human proangiogenic monocytes (HLA-DR+CD16+). Human HLA-DR staining identified human monocytes recruited to DLD1 xenograft. Gating on HLA-DR+ cells allowed quantification of human proangiogenic monocytes (CD16+). **c** Quantification of the percentage of total human monocytes within tumour cell suspension (left) and percentage of HPMo within recruited human monocytes (*n* = 12 tumours from six mice per group injected with monocytes from the same donor in each experiment, the data were combined from two independent experiments). About 20–60% of recruited human monocytes were from proangiogenic subset with the highest proportion for xenografts of colorectal adenocarcinoma cells DLD1 and the lowest for xenografts of breast carcinoma SKBR7. The data are presented as boxplot with median (horizontal line in boxes), 25–75th percentiles (box limits) and Min–Max indicated by whiskers. *p < 0.05, **p < 0.001 in one-way ANOVA

We then studied the recruitment of HPMo to the three xenografted human carcinoma cell lines. For this, adoptive transfer of human monocytes was performed after tumours reached 0.5 cm^3^ in volume (Fig. 1a). For appropriate collection and analysis of extravasated monocytes by FACS, the mice were first perfused with EDTA-containing buffer to wash out circulating and adherent leucocytes from blood vessels before tumour tissue was dissociated. Human monocytes were then identified in tumour cell suspensions by staining for HLA-DR as neither mouse cells nor human tumour cells expressed this human MHC class II molecule (Fig. 1b). Both, human non-angiogenic and angiogenic monocytes migrated into the tumours. Notably, the proportion of HPMo within recruited human monocytes was highest in DLD1 colorectal tumours, intermediate in HCT116 and lowest in SKBR7 grafts (Fig. 1c). In all xenografts, the proportion of HPMo within recruited monocytes was substantially increased compared to donor blood (20–60% versus 15%; Fig. 1c, right plot, red dashed line). By confocal microscopy, we also analyzed the distribution of HPMo in DLD1 tumours after recruitment by staining for human TIE2, expressed only by human angiogenic monocytes in the xenografts (Supplementary Figure 5). Interestingly, HPMo were found gathered around blood vessels 4 h after monocyte injection, which became spread throughout the tumour after 8 h. These results together indicate that human colorectal tumour xenografts create microenvironmental conditions that preferentially recruit human proangiogenic monocytes into tumour tissue due to factors in addition to inflammatory cytokines.

### Proangiogenic monocyte homing due to tumour inflammation

**Fig. 2:**
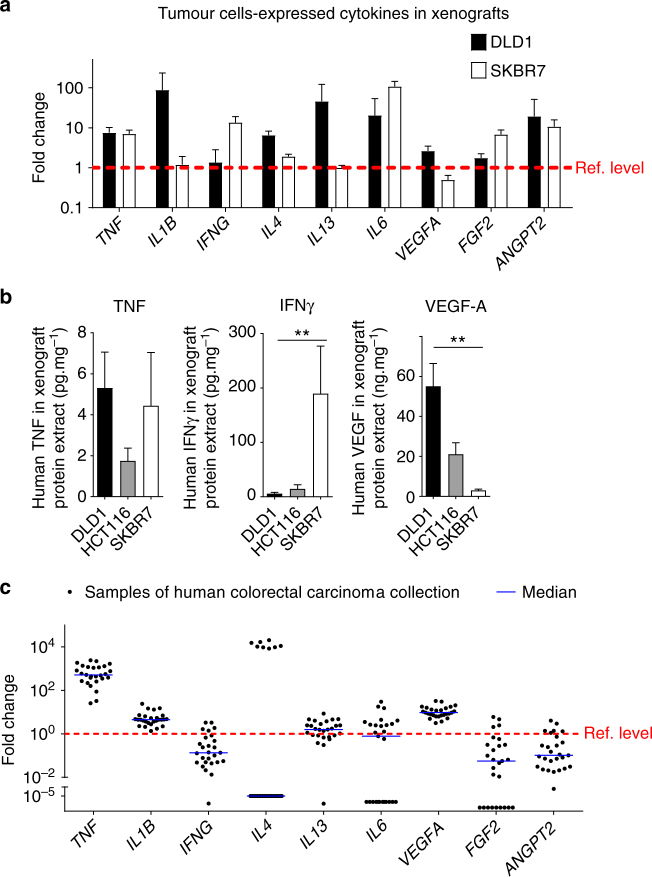
Human tumours express a panel of cytokines and angiogenic factors. **a** Screening of human tumour cells-expressed cytokines (screened with human primers) in the xenografts (*n* = 6 tumours per group pooled from two independent experiments). All molecule expression was normalised against the expression of housekeeping genes, namely *GAPDH* and *ACTB*. The expression levels of cytokines in DLD1 and SKBR7 xenografts are presented as relative (fold change) to their expression levels in HCT116 xenograft, which showed intermediate recruitment levels of proangiogenic monocytes. **b** Confirmation of cytokine expression level in tumour xenografts by ELISA. The protein levels of TNF, IFNγ and VEGF-A were quantified in protein extract of DLD1, HCT116 and SKBR7 xenografts (*n* = 5 samples per tumour type) by ELISA. The data are presented as mean ± s.d., **p-value <0.005 in Kruskal–Wallis test. **c** Cytokine and angiogenic factor expression in samples of human colorectal carcinoma from the CREMEC tumour collection. The expression levels in samples (*n* = 27 xenografts of independent human primary colorectal tumours from different individuals) are presented as relative (fold change) to their levels in HCT116 xenograft. The red dashed line indicates the reference level

To understand the inflammatory conditions enabling the entry of human proangiogenic monocytes to solid tumours, we first investigated the expression pattern of human cytokines and angiogenic factors by qPCR (Fig. 2a). As illustrated for colorectal carcinoma DLD1 and breast carcinoma SKBR7 xenografts, a large panel of cytokines and angiogenic growth factors were analyzed. They include tumour necrosis factor (TNF), interleukin-1β (*IL1B*)*, IL4, IL13*, vascular endothelial growth factor A (*VEGFA*) and angiopoetin 2 (*ANGPT2*). *TNF*, *IL6* and *ANGPT2* were expressed equally by both DLD1 and SKBR7 xenografts, whereas higher levels of *IL1B, IL4, IL13* and *VEGFA* were found in DLD1 tumours. Conversely, SKBR7 xenografts expressed higher levels of *IFNG* and *FGF2*. We analyzed TNF, IFNγ and VEGF-A in protein extracts of the three xenografts by enzyme-linked immunosorbent assay (ELISA) (Fig. 2b). As found in the mRNA screening, the level of TNF was not significantly different between the three tumour xenografts, IFNγ was high in xenografts of breast carcinoma SKBR7 and very low in the two colorectal carcinomas DLD1 and HCT116, whereas VEGF-A was high in the colorectal tumour xenografts and low in breast carcinoma. This supported the expression patterns observed in the mRNA screening. To further support the cytokine expression pattern found in xenografts of DLD1 tumours, we analyzed the expression levels of the same factors in 27 xenografts of primary human colorectal tumours, all engrafted in CB17-SCID mice immediately after resection from patients (CREMEC collection)^17^ (Fig. 2c). Concordantly, the xenografts of primary human colorectal tumours showed high amounts of *TNF, IL1B, IL13* and *VEGFA*. In contrast, the levels of *IFNG, FGF2* and *ANGPT2* were low in these samples comparable to the DLD1 xenografts. Altogether, these results have demonstrated that human tumour xenografts, which recruit human angiogenic monocytes, present a particular inflammatory environment that presumably modulates the extravasation of human proangiogenic monocytes to tumours by acting on tumour blood vessels. Therefore, we experimentally tested this possibility by using monocyte transendothelial migration (transmigration) under flow conditions.

### IFNγ hampers transmigration of proangiogenic monocytes

**Fig. 3:**
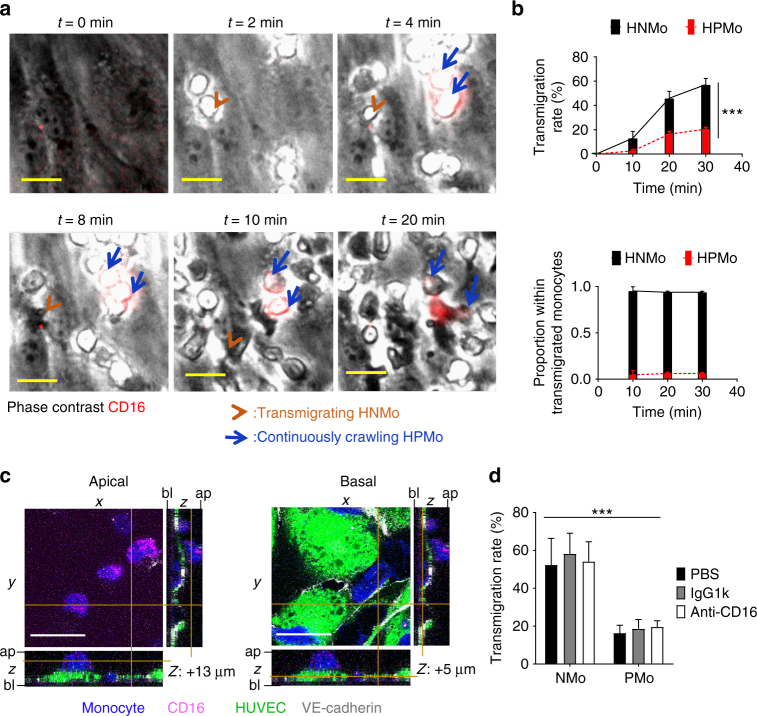
Angiogenic monocytes present low transmigration in conventional inflammatory conditions. **a** Prongiogenic (non-classical and intermediate CD16+) versus non-angiogenic (inflammatory CD16-) monocyte transmigration through TNF-activated endothelium under flow assay by live imaging. Non-angiogenic monocytes transmigrate through TNF-activated endothelial monolayer, whereas angiogenic monocytes adhere but remain crawling with very few transmigrating. Scale bar = 10 µm, blue arrows indicate crawling proangiogenic monocytes and brown arrowhead shows a transmigrating non-angiogenic monocyte. **b** Quantification of the transmigration efficiency of proangiogenic versus non-angiogenic monocytes overtime (top) and the proportion of monocyte subsets within the transmigrated pan-population (bottom) indicating most abluminal monocytes were non-angiogenic under conventional inflammatory conditions. *n* = 3 independent experiments with monocytes from three different donors, the data presented as mean ± s.e.m., ***p-value = 0.0004 in two-way ANOVA test. **c** Post-transmigration assay analysis of proangiogenic monocyte localisation by confocal microscopy. Scale bar = 10 µm, orthogonal presentation shows views from *x*, *y* and *z* axes. Basal (bl) and apical (ap) extremities are indicated. HUVEC and monocytes were pre-stained respectively with green and blue cell tracker, and proangiogenic monocytes and cell junctions were stained respectively with anti-CD16 and anti-VE-cadherin after transmigration assays. Orthogonal views show adherent but non-transmigrated proangiogenic monocytes on endothelial luminal surface. **d** Effect of CD16 labelling on proangiogenic monocytes transmigration analyzed after transmigration assay. *n* = 4 independent experiments with monocytes from four different donors, 150 individual cells tracked for each condition, the data presented as mean ± s.d., ***p-value <0.0001 in two-way ANOVA. The monocyte positioning was unaffected by labelling with the CD16 antibody used for tracking proangiogenic monocytes

**Fig. 4:**
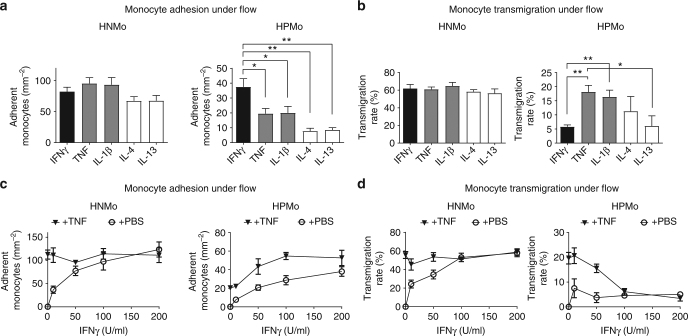
IFNγ increases proangiogenic monocyte adhesion but abolishes transmigration. **a** Analysis of proangiogenic versus non-angiogenic monocyte adhesion to endothelial monolayer activated with different inflammatory cytokines. *n* = 4 independent experiments with monocytes from four different donors, cell counted in 1 mm^2^ area, the data presented as mean ± s.d., *p-value <0.05, **p-value <0.005 in ANOVA test. Proangiogenic monocytes adhered at higher levels to IFNγ-activated endothelium. **b** Analysis of proangiogenic versus non-angiogenic monocyte transmigration to the endothelial monolayers activated with indicated inflammatory cytokines. *n* = 4 independent experiments with monocytes from four different donors, 40 individual cells tracked per condition, the data presented as mean ± s.d., *p-value <0.05, **p-value <0.005 in ANOVA test. IFNγ-activated endothelium exhibited the lowest transmigration rate for proangiogenic monocytes. **c** Analysis of the dose-response effect of IFNγ on monocyte subset adhesion to TNF-activated endothelial monolayer. *n* = 3 independent experiments with monocytes from three different donors, cell counted in 1 mm^2^ area, the data presented as mean ± s.d. IFNγ induced a dose-dependent increase in proangiogenic monocyte adhesion to TNF-activated endothelium. **d** Effect of IFNγ on monocyte transmigration through TNF-activated endothelial monolayers. *n* = 3 independent experiments with monocytes from three different donors, the data presented as mean ± s.d. Increasing concentrations of IFNγ induced a dose-dependent inhibition of proangiogenic monocyte transmigration through TNF-activated endothelial monolayers

We first used time-lapse imaging of monocyte transendothelial migration under flow to understand how extravasation of human non-angiogenic versus proangiogenic monocytes would be influenced by endothelial stimulatory cytokines (Supplementary Note 4, Supplementary Figure 6a, b, Supplementary Movies 1, 2)^18–21^. This method allows simultaneous investigation of the recruitment of both monocyte subsets (Supplementary Figure 6c,d). We used non-blocking fluorescently labelled CD16^−^ specific antibody to identify HPMo in the admixed HPMo/HNMo population (Supplementary Figure 7). When the endothelial monolayer was stimulated with TNF, the inflammatory cytokine that was equally expressed in all the three human xenografts, both HPMo and HNMo were captured at identical rates (Supplementary Figure 7a). The proportion of adherent and circulating HPMo was similar (Supplementary Figure 7b, c). Surprisingly, HPMo did not adhere to PBS-treated endothelial monolayers under flow conditions. Although endothelial monolayer activation with TNF-induced HPMo adhesion, only a low proportion of the adhering cells transmigrated, while inflammatory monocytes transmigrated (Fig. 3a, b, Supplementary Movie 3). Indeed, HPMo remained on TNF-stimulated endothelial luminal surfaces crawling in a manner consistent with a patrolling monocyte^6,12^. This monocyte positioning was confirmed by confocal microscopy, where endothelial cells and pan-monocytes were pre-stained respectively with green and blue cell tracker dyes (Fig. 3c). Confocal imaging confirmed that CD16^+^ monocytes remained on the luminal surface (apical), whereas inflammatory CD16^−^ monocytes populated the abluminal compartment of the endothelium (basal). To exclude effects of the labelling antibody on HPMo transmigration, pan-monocytes were pre-incubated with either PBS, control IgG or unlabelled anti-CD16 and the cell positioning after recruitment under flow was analyzed by confocal microscopy. There was no adverse effect of the antibody on monocytes (Fig. 3d). In conclusion, stimulation of vascular endothelium with TNF, which was equally present in DLD1 and SKBR7 tumour xenografts, led to continuous crawling of HPMo and induced transmigration of inflammatory monocytes. Endothelial activation by inflammatory cytokines IL-1β, IL-4 or IL-13, though higher in DLD1 grafts, showed similar effects as shown with TNF (Fig. 4a, b). Surprisingly, the endothelial monolayer activation with IFNγ which was at high levels in SKBR7 tumours, showed a twofold increased adhesion of HPMo, but even less transmigration of these cells. This suggests that IFNγ also found at low level in human colorectal cancer samples, might have a deleterious action on HPMo recruitment by causing their continuous crawling on the endothelial surface. The presence of IFNγ also increased TNF-induced HPMo adhesion but reduced their transmigration, both in a dose-dependent manner (Fig. 4c, d). These results showed that inflammatory cytokines activate the endothelium to allow preferential transmigration of non-angiogenic inflammatory monocytes and particularly IFNγ amplifies endothelial mechanisms that specifically promote adhesion and crawling of proangiogenic monocytes but inhibit their transmigration.

### VEGF-A tunes endothelial inflammation and HPMo transmigration

**Fig. 5:**
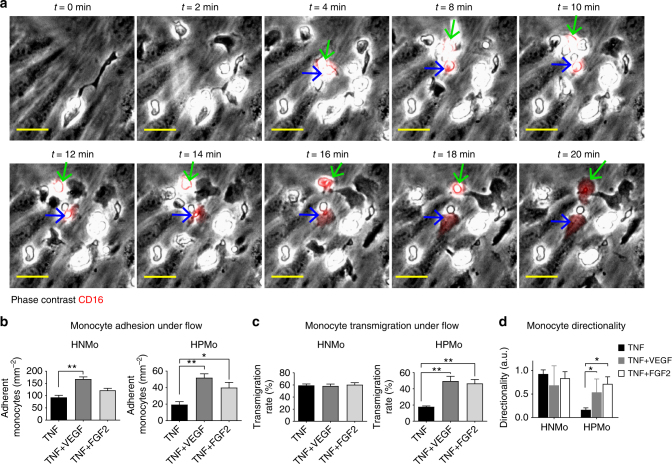
Human proangiogenic monocytes transmigrate in angiogenic factor-driven inflammation. **a** Time-lapse imaging of proangiogenic monocyte transmigration assay under flow through endothelium activated with a combination of TNF and VEGF-A. Scale bar = 25 µm. Green and blue arrows indicate two transmigrating proangiogenic monocytes. **b** Analysis of proangiogenic and non-angiogenic monocyte adhesion in ADIn (TNF + VEGF-A or TNF + FGF2) versus conventional (TNF alone) inflammation under flow. **c** Analysis of proangiogenic and non-angiogenic monocyte transmigration in ADIn versus conventional inflammation under flow. The data are presented as mean ± s.d., *p-value <0.05 and **p-value <0.005 in ANOVA test. **d** Analysis of the directionality of monocyte subsets in ADIn versus conventional inflammation. The data in **d** are presented as mean ± s.d., * indicates *p*-value <0.05 in Mann–Whitney test. The data were from 4 independent experiments

In addition to some inflammatory cytokines, the angiogenic factor VEGF-A was highly expressed by DLD1 xenografts. We therefore investigated the influence of angiogenic cues on transmigration of HPMo. Activation of endothelial monolayers with the angiogenic factors VEGF-A, ANGPT2 or FGF2 alone did not induce monocyte adhesion to endothelial cells under flow (data not shown). However, activation of endothelial cells with a combination of TNF and angiogenic factors substantially changed the profile of HPMo recruitment by increasing both adhesion and transmigration (Fig. 5a, Supplementary Movie 4). We described this set of conditions as angiogenic factor-driven inflammation (ADIn) as a similar outcome was found with another angiogenic factor FGF2 (Fig. 5a, b). Strikingly, ADIn-induced rapid transmigration of HPMo, comparable to rates found for inflammatory monocytes (Fig. 5c). The measurement of HPMo trajectory and calculation of their directionality showed that on ADIn-activated endothelium, proangiogenic monocytes crawled shorter distances before rapid transmigration (Fig. 5d). Confocal imaging after recruitment under flow confirmed the transmigration of HPMo into the abluminal compartment (Supplementary Figure 8). These results demonstrated that combined action of VEGF-A and TNF on the endothelium actively induces adhesion and transmigration of HPMo and suggested that the high expression of angiogenic factors by tumour cells plays a central role in augmenting proangiogenic monocyte extravasation.

### VEGF and IFNγ antagonize endothelial CX3CL1 expression

**Fig. 6:**
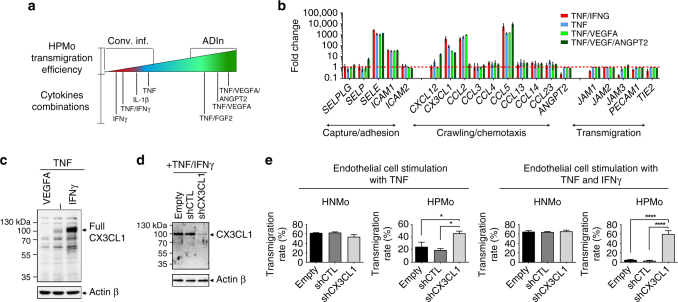
High-CX3CL1 expression regulates the luminal retention of proangiogenic monocytes. **a** Scheme of proangiogenic monocyte transmigration map indicating how cytokines and combinations of factors affect proangiogenic monocyte transmigration. **b** Screening by qPCR for differential mRNA expression by endothelial cells of molecules involved in leucocyte recruitment including capture, crawling and transmigration, after indicated stimulations. Normalisation was performed with housekeeping gene mRNA levels namely *ACTB* and *GAPDH*. The level in inflammation is expressed as the fold-change of expression level compared to non-stimulated endothelial cells. The horizontal dashed line (red) indicates baseline level. *n* = 3 technical replicates, representative of three independents experiments, the data are mean ± s.e.m. **c** Protein expression of CX3CL1 by endothelial cells upon activation with different cytokines and combination of factors. **d** Western blot showing efficient downregulation of CX3CL1 expression in HUVEC after transfection with a plasmid expressing *CX3CL1*-targeting shRNA. **e** Effect of CX3CL1 downregulation on transmigration of monocyte subsets through TNF or TNF + IFNγ-activated endothelium. *n* = 4 independent experiments with monocytes from four different donors, the data are presented as mean ± s.e.m., *p < 0.05; ****p < 0.0001 in ANOVA test

To decipher the endothelial mechanism leading to human proangiogenic monocyte transmigration, we first outlined an endothelial “stimulation map” with the corresponding efficiency of HPMo transmigration (Fig. 6a). Based on this map, we performed endothelial activation with combinations of cytokines and analyzed differential expression of molecules involved in leucocyte capture, crawling and transmigration between angiogenic factors-driven and conventional inflammations with inflammatory cytokines (Fig. 6b). Indeed chemokines and adhesion molecules are primary candidate molecules that can potentially influence these functions^22^. Notably the expression profile of *CX3CL1* was high in conventional inflammatory conditions and lower in ADIn. CX3CL1 is a unique chemokine existing in soluble and transmembrane forms, the former involved in chemotaxis and the latter in adhesion of leucocytes. Increased transmigration of human proangiogenic monocytes was associated with low *CX3CL1* expression (Supplementary Table 1). The protein expression pattern of CX3CL1 followed the trends of mRNA screening (Fig. 6c, Supplementary Figure 22). These results suggested CX3CL1 involvement in a process of HPMo retention during conventional stimulation of inflammation inducing CX3CL1 accumulation on the endothelial luminal surface as determined by confocal microscopy (Supplementary Figure 9a). The higher expression levels of the CX3CL1 receptor CX3CR1 by HPMo in comparison with HNMo reinforced this hypothesis and is consistent with previous studies^23,24^ (Supplementary Figure 9b). To investigate the existence of the luminal retention of HPMo during conventional inflammation, we stably downregulated CX3CL1 expression in endothelial cells by short hairpin RNA (shRNA) strategy (Fig. 6d). When these endothelial cells were activated with TNF + IFNγ combination, we observed a striking decrease in the level of HPMo adhesion (Supplementary Figure 9c). This was not seen when TNF was applied alone. In addition, CX3CL1 stable downregulation improved HPMo transmigration in conventional inflammatory conditions demonstrating a specific interference of CX3CL1 with extravasation of this monocyte subset (Fig. 6e). These results demonstrate that the high expression of membrane CX3CL1 promotes HPMo retention on endothelium during conventional inflammation, whereas angiogenic factors-driven inflammation reduces CX3CL1 expression and leads to a rapid transmigration of HPMo.

### VEGF-A modifies the inflammatory program of tumour vasculature

**Fig. 7:**
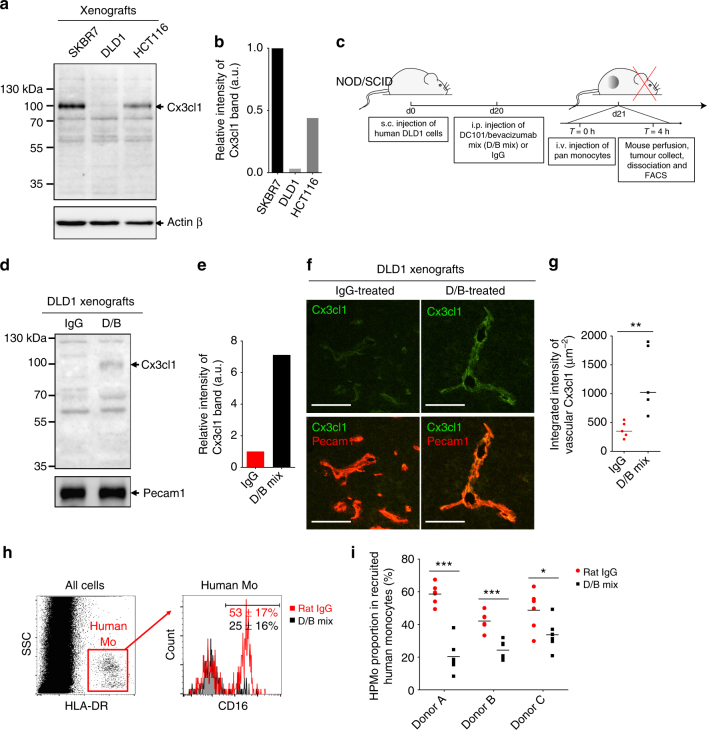
Blocking VEGF-A signalling inhibits homing of proangiogenic monocytes to tumours. **a** Analysis of Cx3cl1 protein expression in xenografts of SKBR7, DLD1 and HCT116 tumours. **b** Quantification of Cx3cl1 band intensity in **a** after normalisation with same sample Actin level. **c** Scheme of the study of VEGF-A implication in human monocyte recruitment in DLD1 xenograft. DLD1 xenograft-bearing mice were treated either with the mixture of DC101 and bevacizumab (D/B mix) or control IgG for 24 h before adoptive transfer of human pan monocytes. The mice were perfused with PBS-EDTA to wash out circulating and non-extravasated leucocytes before tumour collect and analysis. **d** Analysis of Cx3cl1 protein level in DLD1 tumour after 24 h of D/B mix treatment by western blotting. **e** Quantification of Cx3cl1 band intensity in **d** after normalisation with Pecam1 level in same samples. **f** Expression level of Cx3cl1 in tumour vasculature by immunofluorescence of DLD1 xenografts. Scale bar = 80 µm. **g** Quantification of Cx3cl1 in blood vessels. Data are presented as scatter dot plots with medians. Each dot represent the mean intensity of Cx3cl1 per µm^2^ blood vessels from 10 sections of the same tumour. *n* = 5 tumours per group were used for this quantification. **p < 0.005 in Mann–Whitney test. **h** FACS analysis of the effect of D/B mix on human proangiogenic monocyte recruitment to DLD1 xenograft. **i** Effect of blocking VEGF-A signalling on extravasation of proangiogenic monocytes from different donors. The data presented as aligned dot plot with medians. *n* = 6 tumours per group in independent adoptive transfer experiments with monocytes from three donors (DonorA, DonorB and DonorC). *p < 0.05, **p < 0.005, ***p < 0.001 in multiple *t*-test

Our results suggested that expression of VEGF-A by tumour cells along with the inflammatory cytokine TNF might program tumour vasculature to maintain expression of CX3CL1 at low levels enabling extravasation of HPMo. Consistently, CX3CL1 levels in protein extracts of xenografts were highest in breast carcinoma SKBR7 and barely detectable in colorectal DLD1 tumours (Fig. 7a, b). As a reminder, human colorectal and breast tumours express respectively high and low levels of VEGF-A (Fig. 2b). We therefore tested in vivo whether blocking VEGF-A activity would downregulate CX3CL1 in colorectal carcinoma grafts and lead to extravasation of HPMo (Fig. 7c). The mice bearing DLD1 xenografts were treated with a mixture of antibodies against mouse VEGFR2 (DC101) and anti-human VEGF-A (Bevacizumab), (D/B), 24 h before analysis of CX3CL1 expression or adoptive transfer of human monocytes (Fig. 7d–i, D/B mix). This short interference with VEGF-A signalling triggered upregulation of CX3CL1 expression in blood vessels of DLD1 xenografts without affecting the tumour vessel density or tumour size (Fig. 7d, e, Supplementary Figure 10). This demonstrated VEGF-A involvement in the inflammatory reprograming of blood vessels in colon carcinomas (Fig. 7f, g). Concordantly, the adoptive transfer of human monocytes, by i.v. injection 24 h after D/B mix treatment, led to reduced percentages of proangiogenic subsets within recruited human pan-monocytes (Fig. 7h, i). Altogether, these results demonstrated the crucial feature of VEGF-A in inflammatory programming of tumour vasculature, repressing CX3CL1 expression and enabling extravasation of proangiogenic monocytes to solid tumours. In addition, we found that in a non-tumoral peritonitis model in NOD/SCID mice, angiogenic factor supplementation induced proangiogenic monocyte extravasation (Supplementary Note 5, Supplementary Figure 11) showing that they have the capacity to extravasate into non-tumour environments if ADIn is present, suggesting also a potential recruitment mechanism for these cells in chronic inflammation.

### GATA3 alters endothelial CX3CL1 expression during ADIn

**Fig. 8:**
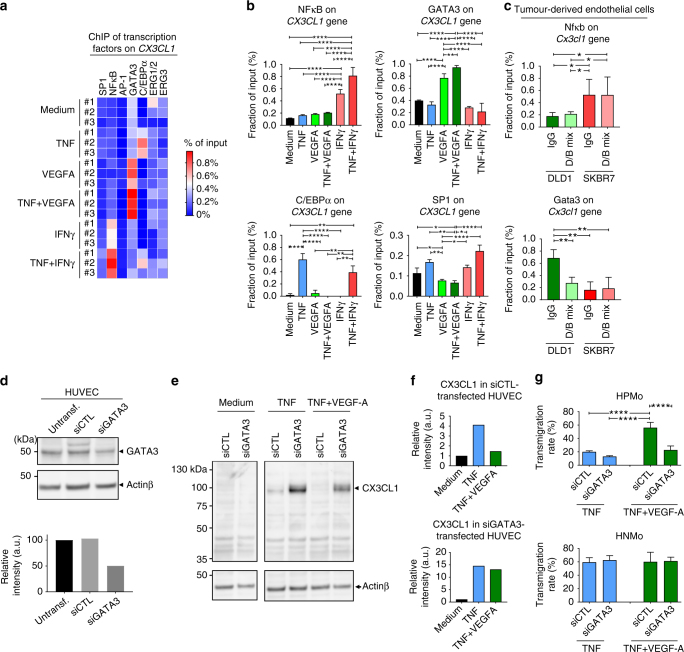
VEGF-A promotes GATA3-mediated alteration of *CX3CL1* expression and HPMo homing. **a** Heat map of the influence of inflammatory conditions on transcription factor binding to the promoter of *CX3CL1* in HUVECs. HUVECs were stimulated with inflammatory regimens, then chromatin immunoprecipitation (ChIP) was performed with antibodies against indicated transcription factors and immunoprecipitated DNA fragments in parallel with total input DNA were analysed. Analysis was done by qPCR for the presence of the *CX3CL1* promoter. The result is presented as percentage of input DNA. The data represent individual mean values of experimental replicates (3 experiments #1 to #3). **b** Analysis of the effect of inflammatory conditions on the binding of candidate transcription factors to the *CX3CL1* promoter from **a**. Data are presented as mean ± s.d. (*n* = 3 experiments). **c** Analysis of the binding of Nfκb and Gata3 to *Cx3cl1* promoter in tumour associated endothelial cells. DLD1 and SKBR7 xenograft-bearing mice were treated either with the mixture of DC101 and bevacizumab (D/B mix) or control IgG for 24 h. Mice were killed, and tumours collected to isolate endothelial cells for ChIP analysis of Nfκb and Gata3. Data are presented as mean ± s.d. of experimental replicates (*n* = 3). **d** Analysis of GATA3 downregulation in HUVEC with specific siRNA by western blotting and quantification. **e** Effect of GATA3 downregulation on CX3CL1 protein expression induced by TNF and TNF + IFNγ in HUVECs. **f** Quantification of GATA3 expression in **e**. **g** Role of GATA3 in HPMo transmigration induced by angiogenic factors-driven inflammation. The data are presented as mean ± s.d. of three independent experiments. *p < 0.05, **p < 0.001, ****p < 0.0001, in ANOVA for **b**, **c** and **g**, analysis with Holm-Sidak adjustment for multiple comparison

We further explored the mechanisms of VEGF-A-induced transcriptional programing of vascular inflammation, leading to alteration of CX3CL1 expression and promoting HPMo extravasation. Transcriptional regulation of a gene typically occurs either by chromatin modification or by the control of transcription factor binding to the gene promoter^25^. As gene repression can be caused by chromatin methylation, we then analyzed the methylation status of histone H3 around *CX3CL1* in HUVECs, activated by different inflammatory conditions. This was studied by chromatin immunoprecipitation (ChIP) and analyzed by qPCR. We found that the ChIP of trimethyl-H3(Lys9) showed no difference in histone methylation around *CX3CL1* by the inflammatory conditions applied to HUVECs (Supplementary Figure 12). We then performed ChIP on a panel of transcription factors interacting with the *CX3CL1* gene as defined by the SABiosciences’ proprietary database DECODE (Fig. 8a, b; Supplementary Figure 12). The analysis of co-precipitated DNA by qPCR showed the presence of DNA fragments corresponding to *CX3CL1* and control genes for each of the transcription factors, attesting the functionality of the ChIP assay. We found changes in the binding of NFκB, GATA3, SP1 and C/EBPα to *CX3CL1* (Fig. 8a, b). Indeed TNF alone or in combination with IFNγ strongly induced C/EBPα and SP1 binding to *CX3CL1*. Interestingly, in addition to these transcription factors, TNF combination with IFNγ led to the binding of NFκB. Conversely, the presence of VEGF-A led to alteration of TNF-induced C/EBPα and SP1 binding while provoking a striking interaction of GATA3 with *CX3CL1* promoter. Given the existence of NFκB and GATA3 binding sites also in the mouse *Cx3cl1* gene, we further investigated the binding of these transcription factors with *Cx3cl1* in tumour endothelial cells and eventual involvement of VEGF-A signalling in this process. Therefore NOD/SCID mice bearing DLD1 and SKBR7 xenografts were treated with either IgG or D/B mix to target VEGF-A signalling for 24 h before tumour collect and tumour-associated endothelial cell sorting for ChIP of NFκB and Gata3. We found that NFκB interaction with *Cx3cl1* was higher in endothelial cells of SKBR7 grafts compared to DLD1 tumours (Fig. 8c). Conversely, Gata3 binding to *Cx3cl1* was higher in endothelial cells from DLD1 tumours. Interestingly, targeting VEGF-A signalling decreased this binding of Gata3 in DLD1, whereas this had no effect on NFκB/*Cx3cl1* interaction in SKBR7 grafts. Altogether, these results suggested that VEGF-A-induced amplification of GATA3 binding to the *CX3CL1* gene may disturb the chemokine expression in endothelial cells thus enabling HPMo transmigration. Concordantly, this hypothesis was supported by the translocation of GATA3 from cell cytoplasm to the nucleus of TNF-activated HUVECs induced by VEGF-A supplementation (Supplementary Figure 13).

We tested this hypothesis by downregulating GATA3 expression in HUVECs by using small interference (si)RNA and analyzed the expression of CX3CL1 protein under stimulation by TNF or TNF in combination with VEGF-A (Fig. 8e, f). The downregulation of GATA3 led to a stronger induction of CX3CL1 in HUVECs under TNF stimulation. VEGF-A could no longer revert CX3CL1 expression in cells transfected with GATA3 siRNA. In addition GATA3 downregulation inhibited HPMo transmigration under ADIn without affecting HNMo transmigration (Fig. 8g). These results demonstrated the repressing consequence of GATA3 binding to the *Cx3cl1* gene and the requirement of GATA3 in endothelial cells for ADIn-induced repression of CX3CL1 expression, as well as subsequent HPMo transmigration.

### CX3CL1 alters HPMo activation by CCL2 and CCL5

**Fig. 9:**
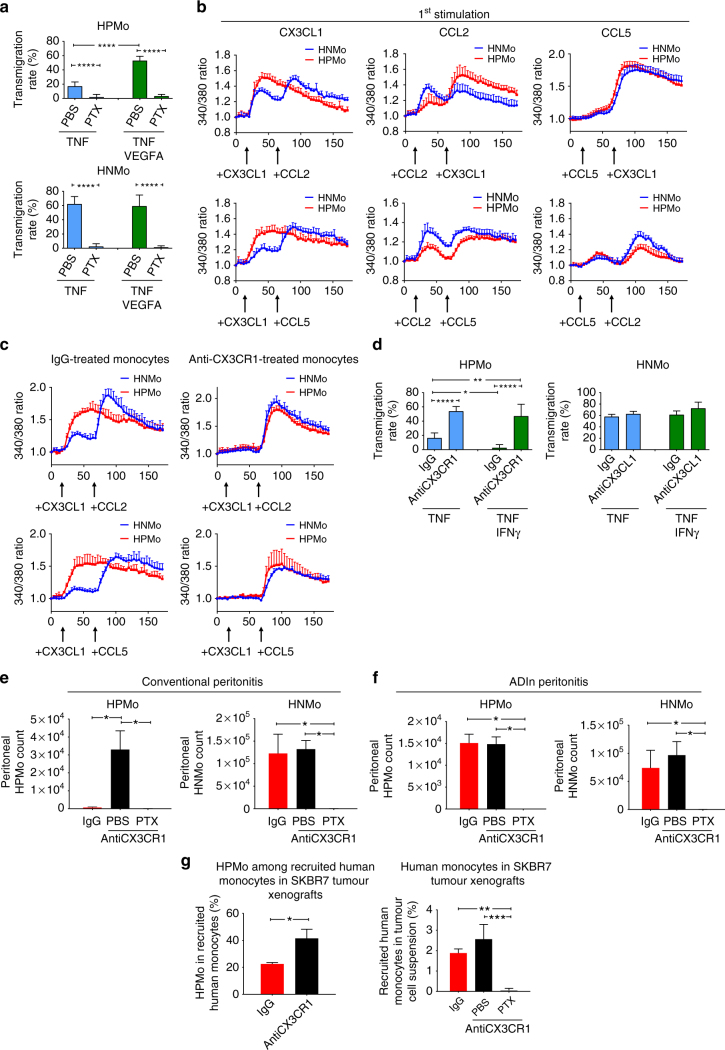
CX3CL1 prevents HPMo activation by chemokines required for monocyte homing. **a** Role of chemokines in HPMo transmigration promoted by angiogenic factors. Human monocytes were treated with PBS or pertussis toxin (PTX) at 1 µg/ml before applying them under flow to HUVECs prestimulated with TNF or TNF + VEGF-A. *n* = 3 experiments, data are mean ± s.d., two-way ANOVA with Holm-Sidak adjustment. **b** Kinetics of calcium flux in monocyte induced by sequential stimulations with chemokines analyzed by Fura2 QBT ratiometric assay. Black arrows indicate the time of chemokine addition. The chemokines were tested at 50 ng/ml. **c** Effect of blocking CX3CR1 on calcium flux induced by sequential stimulation of monocytes by chemokines. Monocytes were treated with either rabbit IgG or anti-CX3CR1 at 50 µg/ml prior to sequential activation. Data were reproduced in three experiments. **d** Analysis of the effect of CX3CR1 blockade on transmigration of monocyte subpopulations through HUVECs activated by TNF or TNF + VEGF. *n* = 3 experiments, data are mean ± s.d., two-way ANOVA with Holm-Sidak adjustment. **e**, **f** Analysis of the influence of CX3CR1 blockade on extravasation of adoptively transferred human monocytes to mouse peritoneum in thioglycolate +Tnf− (**e**) and thioglycolate +TnfVegf-a- (**f**) induced peritonitis. Peritonitis was induced as described in the schematic in Supplementary Fig. 11. Isolated monocytes were treated with either IgG or anti-CX3CR1 antibody at 50 µg/ml (with/without PTX) prior to mouse injection, *n* = 6 mice per group pooled from two experiments, mean ± s.d., ANOVA. **g** Effect of CX3CR1 blockade on HPMo recruitment to SKBR7 tumour xenografts. Human monocyte recruitment to the xenografts was performed as described in Fig.1 with human monocytes treated with the antibodies at 50 µg/ml (with/without PTX) before mouse injection. Left: Percentage of HPMo within recruited human monocytes, *n* = 9 mice per group injected with monocytes from 3 donors. Data are mean ± s.d., *p-value <0.05, paired *t*-test. Right: quantification of the percentage of total human monocytes within tumour cell suspension, *n* = 9 mice per group injected with monocytes from 3 donors. Data are mean ± s.d., *p-value <0.05, ANOVA

To further understand how ADIn induces HPMo transmigration, we investigated the impact of other chemokines in the angiogenic monocyte transmigration. Therefore, monocytes were treated with pertussis toxin, a potent inhibitor of G protein-coupled receptors including chemokine receptors, before co-culturing with activated HUVECs under flow (Fig. 9a). Interestingly, treatments with pertussis toxin totally blocked the transmigration capacity of both HPMo and HNMo regardless of inflammatory phenotype. This result demonstrated that though the surface expression of CX3CL1 on endothelial cells promoted specifically HPMo continuous crawling, the action of other chemokines was required for their transmigration. To understand how the action of CX3CL1 could take over the effect of other chemokines on HPMo, we tested the hypothesis of a specific CX3CL1–induced desensitisation of angiogenic monocytes for other chemokines. Both HPMo and HNMo express the chemokine receptors CX3CR1, CXCR4, CCR1, CCR2 and CCR5 (Supplementary Figure 14a). We used intracellular calcium flux to monitor the activation of these chemokine receptors. Activation of most of these receptors by specific chemokines individually triggered strong calcium mobilisation in both HPMo and HNMo (Supplementary Figure 14b). Monocytes treatment with pertussis toxin before the assay showed that chemokines-induced calcium mobilisations were dependent on their receptor activation as shown for CX3CL1 and CCL2 (Supplementary Figure 14c). However pertussis toxin did not affect the calcium flux induced by the ionophore ionomycin and thapsigargin, an inhibitor of calcium ATPase of endoplasmic reticulum serving as positive controls.

We next performed sequential double activations with CX3CL1, CCL2 and CCL5 as inflamed endothelial cells also expressed high amount of the chemokines CCL2 and CCL5 in all inflammatory conditions, including ADIn as showed above (Fig. 6b). In sequential double stimulations with chemokines, similar response patterns with two peaks of calcium flux in most cases were observed for HPMo and HNMo, as long as CX3CL1 was not used as the first stimulus (Fig. 9b). Notably, when HPMo were activated first with CX3CL1, no second response was detectable with neither CCL2 nor CCL5. This suggests that the CX3CL1 action dominates over the effect of CCL2 and CCL5 in HPMo, rendering the cells irresponsive to the latter chemokines.

### Targeting CX3CR1 enables HPMo responses to CCL2 and homing

**Fig. 10:**
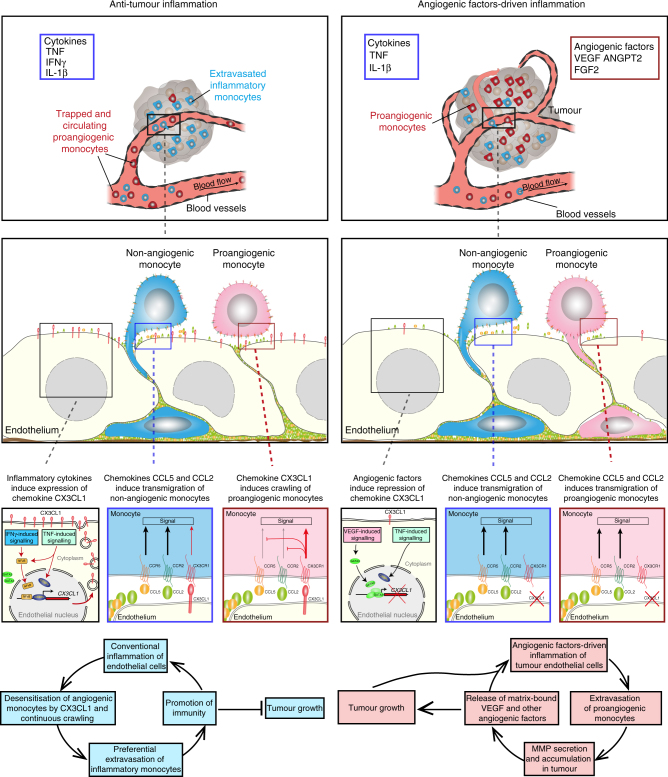
Impact of tumour inflammation on human proangiogenic monocyte extravasation. In conventional inflammation supporting anti-tumour immunity, the increased expression of IFNγ and TNF in the tumour milieu promotes higher endothelial expression of CX3CL1. This is mediated by several transcription factors, including NFkB. Luminal CX3CL1 overrides the action of chemokines CCL5 and CCL2, which are required for transmigration of human proangiogenic monocytes, by mediating a continuous state of crawling on the endothelial vessel wall. Conversely, under tumour-promoting conditions, cancer cells produce angiogenic factors that induce binding of the transcriptional factor GATA3 to the *CX3CL1* promoter. This disturbs expression of CX3CL1 during inflammation, enabling other chemokines, such as CCL5 and CCL2 to trigger extravasation of proangiogenic monocytes. Extravasated human proangiogenic monocytes secrete the protease MMP9 that mobilises extracellular matrix-bound angiogenic factors. Together, these processes boost the recruitment of more proangiogenic monocytes, tumour angiogenesis and tumour growth

We next studied the effect of interference with CX3CR1 function on the responses of monocytes to CCL2 and CCL5. Therefore we first validated the capacity of a polyclonal antibody originally developed against rat Cx3cr1 to block the activation of human CX3CR1 (Supplementary Figure 14d–f). This idea was based on the high conservation of CX3CR1 between rat and human (82% overall sequence similarity). The tested anti-CX3CR1 antibody could stain CX3CR1 on human monocytes and inhibited CX3CL1-induced calcium flux in a dose-dependent manner (Supplementary Figure 14d–f). When CX3CR1 function was blocked in HPMo, they did not respond to CX3CL1 but were strongly activated by CCL2 and CCL5 (Fig. 9c). Calcium flux patterns of HPMo became similar to that of HNMo demonstrating the dominant action of CX3CL1 over CCL2 and CCL5 specifically in HPMo but not in HNMo. In addition, the blockade of monocyte CX3CR1 improved HPMo transmigration through HUVECs activated by TNF alone or in combination with IFNγ in vitro without affecting HNMo transmigration (Fig. 9d). This demonstrated that when the CX3CL1 dominant action was alleviated, HPMo could transmigrate in conventional inflammations induced by TNF and IFNγ. Concordantly, in non-tumoral peritonitis, the blockade of CX3CR1 on human monocytes in vitro before adoptive transfer led to HPMo transmigration (Fig. 9e, f). The extravasation of HPMo induced by CX3CR1 blockade was completely inhibited by additional exposure of the monocytes to pertussis toxin in vitro before their adoptive transfer showing the definitive requirement of HPMo activation by other chemokines for their extravasation. Similar results were also found with SKBR7 tumour xenografts when monocytes were treated with an anti-CX3CR1 antibody in vitro prior to adoptive transfer (Fig. 9g). The blockade of CX3CR1 improved HPMo recruitment to SKBR7 tumours. Again, blocking chemokine receptors with pertussis toxin in vitro before monocyte adoptive transfer inhibited the recruitment of all monocytes to SKBR7 tumours. Altogether these results demonstrated that the chemokines required for human inflammatory monocyte transmigration, such as CCL2 or CCL5, are also needed for human proangiogenic monocyte transmigration. However, their action on angiogenic monocytes becomes negligible in conditions of high expression of CX3CL1, which dominates over CCL2 and CCL5, and imposes a continuous crawling behaviour to HPMo in vessel lumen (Fig. 10).

### Human angiogenic monocytes spontaneously secrete active MMP9

Having established the mechanism of HPMo transmigration, we focused on the proangiogenic function of these cells. We first tested the capacity of HPMo to secrete molecules that could trigger signal-transduction pathways involved in angiogenic activities of endothelial cells, such as activation of mitogen-activated protein kinase (MAPK) or phosphoinositide-3 kinase (PI3K)^26^. Therefore, the conditioned media of HPMo and HNMo were used to stimulate HUVECs. In these endothelial cells, HPMo conditioned medium induced phosphorylation of extracellular signal-regulated kinase (ERK)1/2 and protein kinase B (PKB/AKT), whereas HNMo medium showed no effect (Supplementary Figures 15a and 16). We then analyzed the mRNA expression levels of angiogenic factors in HPMo and HNMo. Surprisingly both monocyte subsets expressed similar mRNA levels of *ANGPT1*, *ANGPT2, VEGFA* and *FGF2* (Supplementary Figure 15b). However, these levels were substantially lower than those of quiescent endothelial cells. As HUVEC and tumour-derived vascular growth factors can bind to extracellular matrix, we explored the hypothesis of growth factor mobilisation caused by monocyte-derived proteases. Therefore, we assayed gelatinase activities in conditioned media of HPMo and HNMo by zymography (Supplementary Figure 15c). HPMo medium contained higher levels of active MMP9 compared to inflammatory monocytes or endothelial cells, whereas MMP2 activity was similar in all conditioned media. Thus, the constitutive secretion of active MMP9 by HPMo may release vascular growth factors from extracellular matrix to trigger MAPK and PI3K activation in endothelial cells.

### MMPs are essential for the proangiogenic activity of HPMo

To challenge the hypothesis of growth factor mobilisation by active MMP9 secreted by HPMo, we used an inhibitor of MMPs combined or not with a VEGF-A blocking antibody. Inhibition of MMP activity in HPMo conditioned medium with GM6001 impaired phosphorylation of ERK and AKT in endothelial cells (Supplementary Figure 17a). Notably, MMP inhibition combined with VEGF depletion did not exhibit an additive inhibitory effect, suggesting a singular mechanism. A similar impact of MMP inhibition and VEGF depletion was observed in endothelial tube formation and proliferation induced by HPMo (Supplementary Figures 17b and 18). As endothelial cells mostly express the inactive form of MMP9, we tested whether activation of endothelial MMPs with aminophenyl mercuric acetate (APMA) would also liberate VEGF and activate the kinases. We found phosphorylation of ERK, AKT and SRC in presence of APMA in a dose-dependent way (Supplementary Figure 19a). The use of genistein, a tyrosine kinase inhibitor, abrogated MMP-induced ERK and AKT phosphorylation, indicating a mobilisation of growth factors bound to extracellular matrix (Supplementary Figure 19b). These controls demonstrate that MMPs produced by HPMo are in active forms while those produced by endothelium are non-active. It also showed that the active form of MMP9 secreted by HPMo is required for the proangiogenic activity. Altogether, this suggests an essential role for MMP9 in VEGF-A mobilisation in the proangiogenic function of HPMo.

### TIMP1 impairs tumour growth induced by angiogenic monocytes

Given our finding that subcutaneous injection of tumour cells into mice simultaneously with human proangiogenic monocytes boosted the development of the colon carcinomas DLD1 and HCT116 but not the breast carcinoma SKBR7, we investigated whether tumour cell-derived inhibitors would block MMP9. Interestingly, the tissue inhibitor of metalloproteinase 1 (*TIMP1*) was highly expressed in SKBR7 breast carcinoma cells (Supplementary Figure 20a). We then experimentally tested the influence of TIMP1 overexpression on DLD1 tumour growth (Supplementary Figure 20b–g). Therefore, the DLD1 cells were stably transfected with either *TIMP1* (*TIMP1*-DLD1) or empty (*empty*-DLD1) vectors. The expression levels of other genes, such as *EGFR* were not affected by TIMP1 overexpression (Supplementary Figure 20b). Xenografts of *TIMP1*-DLD1 cells were smaller than the grafted *empty*-DLD1 or non-transfected DLD1 cells (Supplementary Figure 20d, e). The co-injection of HPMo boosted the growth of empty-DLD1 but failed to sustain the development of timp1-DLD1 tumours. This was not due to the tumour cell intrinsic capacity of proliferation as they showed normal proliferation rates (Supplementary Figure 20c). *TIMP1*-DLD1 tumours showed a drastic reduction of tumour vessel density that could not be improved by HPMo co-injection demonstrating the need of MMP activity for HPMo-induced tumour angiogenesis. Altogether these results confirm the requirement of MMP activity for the proangiogenic function of HPMo to sustain tumour growth.

## Discussion

In this study, we have demonstrated how tumour-derived angiogenic factors specify the inflammatory program of tumour vasculature to enable extravasation of human proangiogenic monocytes. Concordant with the initial work by Venneri et al.^14^, we found that human CD16^+^ patrolling monocytes are proangiogenic cells that boost the growth of human colorectal carcinoma xenografts. This is in striking difference to the mouse system where the inflammatory GR1^+^ monocytes were proposed as a proangiogenic monocyte subset that supports development of primary tumours^2,11,27,28^. Recent work by Hanna et al.^29^ proposed mouse patrolling Ly6C^–^ monocytes (GR1^−^) to limit tumour metastasis to the lung by possibly attacking tumour materials. In view of clinical translation, these functional species differences between human and mouse monocyte subsets suggest that more effort should focus on functional studies of human monocytes to better understand the mechanisms of their recruitment and proangiogenic function in primary tumours.

This is illustrated by the expression of different panels of inflammatory cytokines and angiogenic factors that differentially regulate the homing of human proangiogenic monocytes to tumours. Although human non-classical CD14^dim^CD16^+^ monocytes, the major component of HPMo, were reported to adhere to quiescent endothelium^6,12^, we rarely found CD16^+^ monocyte adhesion to non-activated endothelium under flow. This discrepancy could be due to differences in methodologies with previous studies. However, we found that endothelial activation with inflammatory cytokines induced adhesion of proangiogenic monocytes that mainly remained crawling without transendothelial migration. As for human inflammatory monocytes, the rare transmigrating HPMo used exclusively the paracellular route. Furthermore, we found IFNγ to amplify luminal retention and crawling of human proangiogenic monocytes leading to reduced transmigration. The relatively low level of IFNγ in colorectal carcinoma xenografts correlated well with the high proportion of immigrated angiogenic monocytes. In summary, there is surmounting evidence that IFNγ is a key regulator of extravasation of angiogenic monocytes.

Our study has provided the underlying mechanisms defining IFNγ−induced endothelial cell trapping of HPMo during conventional inflammation. We found that the high-luminal expression of CX3CL1/Fractalkine by endothelial cells is induced by IFNγ as shown previously^30^ and promotes luminal retention of HPMo. This function of CX3CL1 might be specific to its unique features. CX3CL1 is a unique CX3C chemokine expressed as a membrane-bound protein with leucocyte adhesion function^23,24^. It can also be found in a soluble form that has been reported to induce monocyte and lymphocyte chemotaxis^31–33^. Human CD14^dim^CD16^+^ monocytes express higher levels of CX3CR1 compared to inflammatory monocytes, the only receptor for CX3CL1 involved in capture of mouse patrolling monocytes^12^. A recent study proposed that CX3CL1 mediates extravasation of mouse patrolling monocytes in lung vasculature during tumour metastasis^29^. This latter was based on the increased CX3CL1 expression on inflamed blood vessels of mouse lung after intravenous injection of tumour cells, as well as some known deficiencies of CX3CR1−/− mice^12,34,35^. This includes the reduced numbers of patrolling monocytes, an alteration of their “scanning function” and an impairment of their capacity to uptake tumour material. We have provided mechanistic evidence that during conventional inflammation, membrane-bound CX3CL1 promotes continuous crawling of human proangiogenic monocytes. We have also demonstrated that in the presence of CX3CL1, HPMo do not respond to other chemokines normally required for their transmigration. This revisits CX3CL1 functionality for human patrolling monocyte recruitment where luminal expression plays a regulatory role in extravasation.

The exclusion of HPMo induced by IFNγ may be crucial in dictating the efficacy of T lymphocyte action observed in many tumour immunotherapies^36^. Tumour-infiltrated lymphocytes kill tumour cells by cell-mediated cytotoxicity and IFNγ production in tumour immunity^37–42^. The regulatory capacity of IFNγ to dictate luminal retention of proangiogenic monocytes may play a pivotal role in tumour growth inhibition induced by T cells. The clinical use of IFNγ immunotherapy should be considered to efficiently target the recruitment of proangiogenic monocytes and subsequent tumour growth.

Previous studies proposed that hypoxia increases the expression of the CXCL12 receptor CXCR4 on macrophages, including angiogenic, TIE2^+^ cells^43,44^. The secretion of ANGPT2 by tumour cells has been proposed to induce chemotaxis of TIE2^+^ monocytes^14,15,45^. However, ANGPT2 also participates in firm adhesion of all myeloid cells^46,47^. The latter non-specific effect of ANGPT2 and the need of hypoxia for inducing CXCR4 expression on all macrophages suggest that the CXCL12-CXCR4 axis as well as ANGPT2 might regulate interstitial migration of proangiogenic macrophages inside the tumour, rather than initial recruitment through blood vessels. This suggests that other mechanisms account for specific extravasation of HPMo.

In our study, we found that angiogenic factors change the inflammatory program of endothelial cells to allow transmigration of HPMo. Consistently, DLD1 colorectal carcinoma xenografts have shown higher levels of VEGF-A expression and a higher proportion of recruited HPMo, when compared to SKBR7 breast carcinoma xenografts. Although VEGF-A is known to induce vascular permeability to solutes and liquids, this is not sufficient for leucocyte transmigration^48^. In addition, the specific feature of VEGF-A signalling towards promotion of HPMo but not HNMo extravasation, excludes any role of VEGF-A-induced vascular permeability in this process. Concordantly, the simultaneous targeting of mouse Vegfr2 and DLD1 carcinoma-produced VEGF-A with the antibodies DC101 and bevacizumab, respectively, led to increased vascular expression of Cx3cl1 and reduced extravasation of HPMo to DLD1 xenografts. Moreover, although the downregulation of CX3CL1 is critical for HPMo extravasation, other chemokines, such as CCL2 and CCL5, presented only by inflamed endothelial cells, are also required. Non-inflamed endothelium or endothelium exposed to angiogenic factors alone could not enable HPMo extravasation. Furthermore, the high conservation between human and mouse CX3CR1 and the known cross-reactivity of mouse chemokine Cx3cl1 with the human receptor might account for the reduced extravasation of HPMo to tumours induced by the impairment of VEGF-A signalling (Supplementary Figure 21). This process is mediated by GATA3 binding to the *CX3CL1* promoter induced by VEGF-A. It is unclear how VEGF-A/VEGFR signalling triggers GATA3 translocation and binding to the *CX3CL1* gene. Previous studies have shown expression of VEGFR1 by all monocytes/macrophages and implied a role for VEGF-A in pan-monocyte chemotaxis^49–51^. VEGF-A-induced chemotaxis could also contribute to HPMo interstitial migration inside the tumour after extravasation. Thus, the patrolling or extravasation outcome of HPMo recruitment depends exclusively on the surrounding inflammatory environment that instructs the vasculature to enable or prevent HPMo transmigration. However we cannot exclude that in other contexts, alternative mechanisms could regulate the extravasation cascade of proangiogenic monocytes. An improvement in current tumour immunotherapies could be achieved by targeting both HPMo extravasation and interstitial migration. This postulation was supported by two recent studies, where combinations of anti-angiogenic treatments with checkpoint inhibitors demonstrated enhanced antitumour immunity^52,53^.

Our study has provided supporting mechanisms of HPMo function once in the tissue. Indeed the angiogenesis-promoting activity of HPMo is mediated by MMP activity and the release of matrix-bound growth factors including VEGF-A. We found that active MMP9 was highly secreted by human proangiogenic monocytes, which is a feature shared between mouse and human as expression of proMMP9 was previously shown for Tie2^+^ proangiogenic monocytes^5,15^. MMP9 was also proposed to be a key player in VEGF-A release and the angiogenic switch in Rip1-Tag2 mice^54^. The mobilisation of various growth factors from tumour interstitium by active MMP9 might be the major mechanism leading to rebound tumour angiogenesis, resistant to vascular disruption therapies^7,44^. Angiogenic factor mobilisation by MMPs is an amplifying process for angiogenesis. This process needs accumulation of growth factors in extracellular matrix by tumour cells and accumulation of active MMP9 secreted by proangiogenic monocytes. Due to blood flow, both of these events can occur only in the tissue and amplify the arrival of more proangiogenic monocytes. Once in the tumour microenvironment, it is possible that differentiated angiogenic macrophages also secrete angiogenic factors as suggested by previous works in mouse^55,56^.

In summary, here we report a central regulatory process developed by the immune system for tightly controlling the inflammatory outcome according to the tissue environment (Fig. 10). We have therefore postulated that under tumour-promoting conditions, cancer cells can induce angiogenic factors-driven inflammation in endothelium by inducing the transcription factor GATA3 binding to the *CX3CL1* gene that would disrupt expression during this type of inflammation, enabling other chemokines, such as CCL2 to foster proangiogenic monocyte extravasation. This would amplify tumour angiogenesis and support tumour growth through angiogenic factor mobilisation. Such an action would constitute a positive feedback mechanism that would increase the recruitment of more proangiogenic monocytes. During an anti-angiogenic therapy against only one factor (i.e., VEGF-A), proangiogenic monocytes could mobilise other compensatory angiogenic factors to maintain angiogenic factors-driven inflammation and tumour growth supporting angiogenesis. Conversely, in anti-tumour immunity, the high expression of IFNγ in the tumour milieu dictates high-endothelial expression of CX3CL1 that overrides the action of chemokines required for HPMo transmigration, thus imposing a state of continuous crawling for proangiogenic monocytes in the vessel lumen. Therefore, targeting the recruitment of proangiogenic monocytes with anti-angiogenic therapy combined with IFNγ immunotherapy, may be a more valuable strategy to improve anti-tumour therapy.

## Methods

### Mice and human blood samples

Four to six-week-old female NOD/SCID mice were purchased from Charles River Laboratory (Lyon, France). Animals were kept in individually ventilated cages in specific pathogen-free housing conditions in the central animal facility of University of Geneva. In all experiments, NOD/SCID mice were randomly used and assigned to experimental groups with no blinding. For proangiogenic monocyte recruitment study in vivo, all procedures were performed in agreement with the Institutional Ethics Committee of Animal Care in Geneva and the Swiss Cantonal Veterinary Office. Healthy volunteers routinely provided samples of blood used for monocyte isolation and experiments. All experiments using human materials were performed with informed consent of the volunteers.

### Human tumour cell lines

The cell lines of human colorectal adenocarcinoma DLD1 (ATCCCCL-221) and human colorectal carcinoma HCT116 (ATCCCCL-247) were purchased from American Type Culture Collection (ATCC). The human breast carcinoma SKBR7 were a gift from Michel Aurrand-Lions (Marseille, France). Tumour cells were used for engraftment in NOD/SCID mice in agreement with the Institutional Ethics Committee of Animal Care in Geneva and the Swiss Cantonal Veterinary Office.

### Samples of human colorectal tumours from CREMEC project

We used samples from a human tumour collection of primary human colorectal tumours (CREMEC project)^17^. Briefly, colorectal tumour samples were collected after patients’ informed consent. The tumours were engrafted subcutaneously to CB17-SCID mice (Charles River Laboratory). Xenografts were collected when they reached 0.15–0.3 cm^3^ in volume and were frozen at −80 °C to make the xenograft collection^17^. In our study, we analyzed the RNA extracts of 27 different xenograft samples from the CREMEC collection in order to analyze the expression levels of cytokines and angiogenic factors.

### Reagents and antibodies

Total leucocytes were isolated from erythrocyte lysis by treating human peripheral blood with 0.8% ammonium chloride. Lympholyte-H (Cedarlane Laboratories Ltd., Switzerland) or Ficoll-Paque Plus (GE Healthcare) gradients were used to isolate human peripheral blood mononuclear cells (PBMC). For untouched monocytes isolation, the Pan Monocyte isolation kit (Miltenyi Biotech, Germany) was used according to the manufacturer’s protocol. DC101 hybridoma cells (ATCC HB-11534) were used to produce blocking antibody against murine vascular endothelial growth factor receptor 2 (VEGFR2) in order to block the receptor signalling in murine endothelial cells. The antibody produced by this clone was validated in previous studies^57,58^. The antibody bevacizumab (Avastin, Roche) against soluble human vascular growth factor (VEGF)-A was used to deplete the human tumour cells-derived and HUVEC-expressed VEGF-A. The neutralising polyclonal antibody directed against rat CX3CR1 (GTX27200) from GeneTex Inc. was validated in our study to also bind and block the function of CX3CR1 on human monocytes (Supplementary Figure 14d–f). Antibodies used in this study are listed in Supplementary table 2. They were used for: studying monocyte subpopulations by flow cytometry, visualising cells by live-imaging or confocal imaging, chromatin immunoprecipitation (ChIP), functional blocking of chemokine receptor and investigating signalling pathways in endothelial cells. Antibodies were sometimes used as direct fluorochrome/Biotin conjugates, or in conjunction with secondary anti-IgG or streptavidin.

### Human monocyte isolation and cell sorting

PBMCs were isolated from peripheral blood by Ficoll-Paque Plus gradient. PBMC were then counted, the morphology analysed with BD Accuri C6 cytometer (BD Biosciences) and then used for pan monocyte isolation by negative selection using human Pan Monocyte isolation kit (Miltenyi Biotech).

For monocyte subset sorting by FACS, monocytes were enriched with the pan monocyte isolation kit and then incubated with combinations of antibodies and FcR blocking reagent in staining buffer that contained 0.5% bovine serum albumin (BSA), 2 mM EDTA in phosphate-buffered saline (PBS). After staining with a specified combination of antibodies, cells were then washed and incubated with fluorophore-coupled streptavidin when necessary. Cells were next washed and re-suspended in staining buffer containing 7-AAD to exclude non-viable or dead cells from cell sorting. Cells considered as monocytes were negative for granulocyte, lymphocyte, Natural Killer and immature dendritic cell markers (CD15^−^, CD2^−^, CD3^−^, CD56^−^, NKp46^−^, CD1c^−^ and CD141^−^) and positive for antigen-presenting cell and monocyte markers (HLA-DR^+^ and CD300e^+^). Within the pan monocyte population, three subsets (CD14^+^CD16^−^, CD14^+^CD16^+^ and CD14^dim^CD16^+^) were separated according to their expression level of CD14 and CD16 with the MoFlo Astrios cell sorter (Beckman Coulter). The sorted monocyte subsets were used for analysing mRNA expression, angiogenic monocyte identification, calcium flux analysis and adoptive transfer of human monocytes to NOD/SCID mouse experiments. For routine monocyte analysis by cytometry, a Gallios flow cytometer (Beckman Coulter) was used to acquire surface marker information and Flowjo analysis software (Flowjo, LLC) to analyze the data.

For sorting tumour-associated endothelial cells by FACS, human tumour xenografts were dissociated with the tumour dissociation kit and MACS gentle Dissociator (Miltenyi biotech) according to manufacturer instructions. Single-cell suspensions were blocked with FcR blocking reagent containing buffer of 0.5% BSA and 2 mM EDTA in PBS. Cell suspensions were stained for 20 min with combinations of antibodies. Cells were next washed and re-suspended in staining buffer containing 7-AAD to exclude non-viable or dead cells from cell sorting. Cells considered as mouse endothelial cells were Pecam1^+^, Ve-cadherin^+^, Ly-5^−^ and Gp38^-^ with the MoFlo Astrios cell sorter (Beckman Coulter). The sorted cells were used for chromatin immunoprecipitation (ChIP) experiments.

### Xenograft development and human monocyte recruitment

To study the xenograft development of human tumour cell line and the effect of proangiogenic monocytes on their growth, immune deficient NOD/SCID mice were subcutaneously injected with 5 × 10^5^ tumour cells (DLD1, HCT116 or SKBR7) alone or with either CD16+ proangiogenic monocytes (HPMo) or CD16- inflammatory monocytes (HNMo) in a ratio of 4:1 (monocytes: tumour cells). The mice were s.c. injected every 2 days with human colony stimulating factor (CSF)-1 (0.5 mg/kg) based on previous work by Qian et al.^59^. Mice in all groups of the same tumour type were killed when median of the condition of tumour cell alone reached about 0.1 cm^−3^ in volume. The xenograft size was measured after the mouse was killed. To study the involvement of matrix metalloproteinases (MMPs) in proangiogenic function of CD16+ monocytes, DLD1 cells were stably transfected with *TIMP1*-GFP or empty-GFP vectors with neomycin resistance gene (Origene). The GFP + DLD1 cells were sorted by FACS and cultured under the selection with G418, Geneticin (Thermo Fisher Scientific). TIMP1 overexpression was confirmed by quantitative PCR analysis. The effect of TIMP1 overexpression on DLD1 proliferation was checked by the use of fluorescent 5-ethynyl-2′-deoxyuridine (EdU) incorporation assay to monitor DNA synthesis with Click-iT Plus EdU Kit (Thermo Fisher Scientific) according to manufacturer recommendations.

To study human monocyte recruitment to established xenografts, mice were subcutaneously (s.c.) injected with 0.5 mg/kg of human colony stimulating factor^59^ (CSF)-1 (Peprotech) before isolated human pan-monocytes (2 × 10^7^ cells per mouse) were adoptively transferred i.v. to NOD/SCID mice bearing tumour xenografts of 0.5 cm^3^ in volume. After 4 h, mice were perfused with phosphate buffered saline containing 20 mM EDTA, killed and tumours collected for protein extraction, cryosection or dissociation. Tumours were dissociated with the tumour dissociation kit and MACS gentle Dissociator (Miltenyi biotech) according to manufacturer recommendations. Single-cell suspensions were analysed by FACS by excluding 7-AAD+ dead cells. Human monocytes were identified by their expression of human HLA-DR and CD16+ cells among total recruited human monocytes gave the proportion of recruited HPMo. To investigate the role of VEGF-A signalling in HPMo recruitment to the xenografts, NOD/SCID mice bearing DLD1 xenograft of 0.5 cm^3^ in volume, were treated intraperitoneally with a mixture of the antibodies DC101 (40 mg/kg) and bevacizumab (6 mg/kg), targeting mouse endothelial Vegfr2 and human tumour cells-derived soluble VEGF-A respectively or control IgG at the same concentration. After 24 h of treatment, mice were s.c. injected with 0.5 mg/kg of human CSF-1 before isolated human pan-monocytes were adoptively transferred i.v to the mice to analyse monocyte recruitment to the tumours as described above. To study the influence of blocking CX3CR1 function: the commercially available rabbit polyclonal anti-rat CX3CR1 antibody (GTX27200) from GeneTex was dialysed in PBS for 24 h at 4 °C to remove sodium azide before use in the assays. Isolated pan monocytes were incubated with the anti-CX3CR1 antibody at 50 µg/ml in PBS for 20 min in vitro and washed with PBS before adoptive transfer. Part of monocytes treated by anti-CX3CR1 were also treated with pertussis toxin at 1 µg/ml for 1 h before adoptive transfer to study the role of additional chemokine receptors. At least 3 NOD/SCID mice pre-injected s.c. with human CSF-1 (0.5 mg/kg) were used per group for the experiments of tumour xenografts development and human monocyte recruitment to tumour grafts. The experiments were reproduced with at least 2 different blood donors.

### Live-imaging of monocyte recruitment

The recruitment of monocyte subsets was investigated on the basis of previous reports with some modifications in order to fit our experimental settings^18–20^. Briefly, six chambers of µ-Slide VI 0.1 ibiTreat (IBIDI) were coated with 0.2% gelatin (Sigma-Aldrich), 1 mg/ml Collagen G (Biochrom AG) in PBS. HUVEC (3 × 10^4^ cells/chamber) were cultured in complete M199 (20% Fetal Calf Serum, 1% Endothelial Cell Growth Supplement (Thermo Fisher Scientific), 1% penicillin, 1% streptomycin, 0.1 mg/ml heparin sodium, 0.1 µM Hydrocortisone, 10 µg/ml l-ascorbic acid in M199 medium) for 7 days to have tightly junctive monolayer with medium change every 2 days. The monolayers were stimulated for 6 h with the cytokines as indicated in Supplementary Table 5, or combinations at the indicated concentrations unless specified in figures.

Pan monocytes were first stained with blue cell tracker 7-amino-4-chloromethylcoumarin CMAC (Thermo Fisher Scientific) or Celltracker Orange CMRA (Thermo Fisher Scientific). Proangiogenic monocytes were identified by staining with FITC-anti-CD16 or PE-anti-CD16 as suitable. Flow was generated over HUVEC monolayer by perfusing washing buffer (containing 0.2% BSA in M199 prewarmed at 37 °C) over cultured HUVEC monolayers using a calibrated pump (74,900; Cole Parmer). The flow rate was representative of shear rates in post-capillary venules (0.05 Pa). An image of a fixed field was generated every 15 s for 25 min (unless other indicated in figure legends), using combinations of phase-contrast and fluorescence imaging with Axiovert 200 microscope (Carl Zeiss) and images recorded with Openlab software. After 1 min of recording, a switch system was used to deliver pre-stained pan monocytes (3 × 10^6^ cells/ml) under continuous uninterrupted flow for 5 min. The switch back to wash buffer perfusion was performed for the remainder of the experiment. In phase-contrast microscopy, with endothelial cell monolayer serving as a background, we can clearly differentiate between brightened captured monocytes, grey to darker body with brightened edges of crawling non-transmigrated monocytes and completely dark and flat shape of transmigrated monocytes. Monocyte adhesion was presented as the mean adherent monocyte number per mm^2^ in 4 different fields of the same chamber ± standard error of mean. The transmigration rate for a single field was determined after tracking 120 individual monocytes (unless other specified in figure legend) and presented as a percentage of total monocytes counted in these 4 different fields mean ± standard error of mean (s.e.m.). To evaluate the effect of blocking CX3CR1 on human monocytes, isolated monocytes were incubated with dialysed anti-CX3CR1 for 20 min at room temperature before application under flow. To investigate the global role of chemokine receptors in monocytes transmigration, isolated monocytes were treated for 1 h with pertussis toxin at 1 µg/ml before application under flow. For monocyte directionality (Dir), it is calculated as $\rm Dir = \frac{{{\rm ed}}}{\it d}$, ed: Euclidian distance from capture point to transmigration point, *d*: length of the migratory path from capture to transmigration. Directionality is typically determined after tracking 120 individual monocytes (unless other specified in figure legend) and presented as mean ± s.d. The difference was considered significant and indicated on graphs with stars only when *p*-value ≤0.05 after Student’s *t*-test, Mann–Whitney or ANOVA tests as indicated in figure legends. Each graph is representative of at least two independent experiments with 4 fields considered for every conditions.

### Immunofluorescence and confocal imaging

Cryosections of human tumour xenografts were fixed with 10% formalin for 5 min before permeabilizatiion with 0.2% Triton X-100 for 10 min. Sections were blocked for 30 min with PBS containing 2% BSA before staining with primary antibodies (A488-anti mouse Pecam1 and or rabbit polyclonal anti-CX3CL1 antibodies) for 2 h. Sections were washed and stained with a secondary antibody if needed for 1 h. Sections were washed and stained with DAPI for 10 min before mounting with Vectashield HardSet antifade mounting medium and image acquisition with the Nikon Ar1 spectral confocal microscope (Nikon). Tumour vascular density, as well as Cx3cl1 expression level in Pecam1+ areas were quantified with imageJ. Pieces of 4–8 tumours (5–10 sections at different plan) were used in each group comparison.

To analyse human monocyte recruitment to human tumour xenografts by confocal imaging, mice were perfused with PBS-EDTA, killed and the tumours collected for cryosections. The staining were performed as described above for tumour sections. Tumour sections were stained with primary antibodies: rabbit anti-human TIE2 and Rat anti mouse Pecam1 for 2 h. The sections were washed and incubated with specific secondary antibodies. Images were acquired with Nikon Ar1 spectral microscope (Nikon).

For monocyte transmigration under flow, confocal imaging was used to further confirm the luminal or abluminal localisation of captured monocytes. Therefore, after live-imaging of monocyte recruitment under flow, the µ-slide chambers (containing pre-stained green endothelial monolayer, blue/red monocytes with proangiogenic ones also stained with PE-anti-CD16/FITC-anti-CD16) were analysed by confocal imaging using a Nikon Ar1 spectral microscope (Nikon). When necessary, endothelial junctions were stained with VE-cadherin specific antibody after chamber blocking with 2% BSA and 0.1% Triton X-100 in PBS. Stack image acquisition allowed the quantification of adherent monocytes on endothelial cell monolayer luminal surfaces (apical) and transmigrated monocytes in the abluminal compartment of the endothelium (basal).

To analyse of GATA3 distribution in activated HUVECs, monolayers of HUVECs were stimulated by TNF (500 U/ml) with or without VEGF-A (1 µg/ml) for 6 h before fixation with 10% formalin, permeabilization with 0.2% Triton X-100 and staining with primary antibodies against GATA3 and VE-cadherin. Cells were washed and incubated with the adequate secondary antibodies and DAPI to stain the nuclei as well. Images were acquired by confocal microscopy with Nikon Ar1 microscope. GATA3 distribution between the nucleus and the cytoplasm was analysed with ImageJ. The results were presented as percentage of total GATA3 in the cells.

### Monocyte recruitment in non-tumour inflammation in vivo

To analyse human monocyte recruitment in non-tumour inflammation vivo, we performed an adoptive transfer of freshly isolated negatively selected human monocytes to NOD/SCID mice. Briefly, mice were intraperitoneally injected with 500 µl of either PBS (untreated mice), Tnf (10^6^ U/kg) in 4% thioglycolate medium (conventional inflammation) or mixture of Tnf (10^6^ U/kg), Vegf-a (0.2 mg/kg) and Fgf2 (0.5 mg/kg) in 4% thioglycolate medium (Angiogenic factors-driven inflammation). Before human monocyte adoptive transfer, mice were subcutaneously injected with 0.5 mg/kg of human CSF-1. Then, the monocyte preparations were stained with Celltracker Orange CMRA (Thermo Fisher Scientific) and mice intravenously injected with 2 × 10^7^ cells/mouse. For CX3CR1 blocking experiments, isolated monocytes were also incubated with dialysed anti-CX3CR1 (Genetex, GTX27200) at 50 µg/ml in vitro for 20 min followed or not by treatment with pertussis toxin for 1 h in vitro before adoptive transfer. Twenty hours after adoptive transfer, the mice were killed by CO_2_ and the peritoneum washed to collect resident and recruited leucocytes from human and murine origin. Collected cells were blocked with FACS buffer containing 0.5% BSA, 2 mM EDTA in PBS and stained with anti-Ly76 (Ter119) to identify erythrocyte, anti-Ly6G for mouse granulocytes, and anti-CD16 and anti-CD14 to identify recruited adoptively transferred human monocytes. To have an exact estimation of recruited cells, we used Flow-Count Fluorospheres (Beckman Coulter) as advised by the manufacturer. Human monocyte pre-staining with Celltracker Orange CMRA before adoptive transfer allowed their distinction from mouse leucocytes. The data are presented as mean ± s.e.m. or as scatter dot plot with median. The difference between conventional inflammation and ADIn were considered significant and indicated on graphs with stars only when *p*-value ≤0.05 after mixed models testing inflammatory conditions as fixed effects and a random effect of donors on intercept and inflammatory conditions. The results are representative of monocytes from different donors.

### Analysis by quantitative PCR

Throughout this study, different expression profiles of a panel of molecules were analysed either in the human tumour xenografts, primary human colorectal tumour samples, monocyte subsets or endothelial cells activated with cytokines. Therefore total RNA was extracted with an RNA and protein isolation kit (Macherey-Nagel) and MACS dissociator if needed (Miltenyi Biotech). The total mRNA was reverse transcribed with SuperScript II Reverse Transcriptase (Thermo Fisher Scientific) and used for qPCR by the use of specific primers (Supplementary Table 3) and Power SYBR Green PCR Master Mix (Applied Biosystems). The qPCR data were acquired with the StepOnePlus Real Time PCR machine (Applied Biosystems) and the relative quantification was performed with DataAssist3 analysis software (Thermo Fisher Scientific). All molecule expression was normalised against gene expression of specified housekeeping genes, namely *GAPDH* and *ACTB*.

For cytokine and angiogenic factor expression by human tumour xenografts, representative pieces of human tumour xenografts of DLD1, HCT116 and SKBR7 cells were lysed and the total RNA extracted. The expression levels of cytokines and angiogenic factors by the tumour cells were quantified by qPCR. To support the mRNA expression patterns, 5 samples of human tumour xenografts of DLD1, HCT116 and SKBR7 cells were lysed and the protein level of TNF, IFNγ and VEGF-A analysed by enzyme-linked immunosorbent assay (ELISA) with specific kits from affymetrix (human TNF high-sensitivity ELISA, human IFNγ high sensitivity and human VEGFA Platinum ELISA). We also analysed the mRNA extracts of 27 different xenograft samples from the CREMEC collection in order to analyse the expression levels of cytokines and angiogenic factors.

For the analysis of endothelial cells-expressed molecules involved in leucocyte migration and differentially expressed between conventional versus angiogenic factors-driven inflammations, HUVEC monolayers were treated with combinations of cytokines. Total RNA and proteins were extracted from the same samples and analysed first by qPCR. Normalised gene expression (dCt) for each molecule in different inflammatory conditions was tested for association with angiogenic monocyte transmigration by using R software. Association of molecule expression was analysed by performing mixed models testing the inflammatory condition as fixed effect with a random effect of experiments on intercept only. This mRNA screening was supported with protein analysis of CX3CL1 by western blotting to confirm association.

To analyse the expression of angiogenic factors by monocytes, the angiogenic (CD16+) and non-angiogenic (CD16-) subsets were sorted by FACS, the mRNA extracted and the expression level of factors was assayed by qPCR and compared to that of quiescent endothelial cells.

To analyse the expression of MMPs by monocyte subsets, we performed zymography as previously described by Sidibe et al.^60^ using the conditioned media of monocyte subsets collected after 24 h of culture in simple medium.

### Chromatin immunoprecipitation of transcription factors

To study the binding of transcription factors to *CX3CL1* during inflammation, we performed chromatin immunoprecipitation (Chip) experiments with Magnify chromatin immunoprecipitation kit (Thermo Fisher Scientific) according to the manufacturer protocols. Briefly, the candidate transcription factors binding to human *CX3CL1* and mouse *Cx3cl1* gene were defined, as well as their binding position with SABiosciences’ proprietary database DECODE (QIAGEN). Primers were designed around the defined binding position of the transcription factors (binding site ±300 bp).

For ChIP with stimulated HUVECs, the cell monolayers were activated for 16 h with cytokines (TNF at 500 U/ml, IFNγ at 200 U/ml, VEGF-A at 1 µg/ml) and combination of cytokines as indicated in figures. Cells were detached with TrypLE trypsin replacement solution (Thermo Fisher Scientific), the chromatin was crosslinked with 1% formaldehyde before cell lysis and chromatin shearing by sonication for 30 s. The sheared chromatin was used for transcription factor immunoprecipitation according to the manufacturer protocol with 5 µg of antibody per ChIP reaction. Immunoprecipitated transcription factors include SP1, NFkB p65, AP-1, GATA3, C/EBPα and ERG1/2/3. The trimethyl-histone H3 (Lys9) binding to SAT2 satellite repeat was used as positive control of immunoprecipitation. After immunoprecipitation, samples and input DNA (directly from sheared chromatin) were reverse crosslinked with proteinase k treatment at 65 °C and the DNA was also purified with the same kit for analysis by qPCR. The enrichment of the DNA sequences corresponding to *CX3CL1*, as well as positive controls genes was determined by qPCR.

For ChIP of tumour-associated endothelial cells, NOD/SCID mice bearing DLD1 and SKBR7 xenografts of about 0.5 cm^3^ in volume, were injected intraperitoneally with a mixture of the antibodies DC101 (40 mg/kg) and bevacizumab (6 mg/kg) (D/Bmix) or rat IgG (40 mg/kg). After 24 h, the mice were perfused with PBS-EDTA, killed and the tumours collected to make cell suspension with MACS Dissociator (Miltenyi Biotech). Mouse endothelial cells in the cell suspension were stained with Pecam1, Ve-cadherin, Ly-5 and Gp38 targeting antibodies and sorted (Pecam1+/Ve-cadherin+/Ly-5-/Gp38- cells) by FACS with MoFlo Astrios cell sorter (Beckman Coulter). Sorted cells were fixed to crosslink the chromatin with 1% formaldehyde, lysed and the chromatin sheared as described above for HUVECs with Magnify chromatin immunoprecipitation kit (Thermo Fisher Scientific). The transcription factors Gata3 and Nfkb p65 were immunoprecipitated and processed as described above.

The primers listed in Supplementary Table 4 were used in qPCR to analyse the amount of transcription factors on the *CX3CL1* gene in different inflammatory conditions. The list also contains the primers for genes serving as positive controls of individual transcription factors. For data analysis, the signals of immunoprecipitated DNA fragments were normalised as percent of input from the same samples as described previously^61^. We used the following formulae:

1 $${\mathrm{IP}}\,{\mathrm{amount}}\,( {\%} ) = {100} \times {2}^{({\mathrm {Mean}}\,{\mathrm {Input}}\,{C}_{\rm T} - {\rm IP}\,{C}_{{\rm T}})}.$$

This was performed also for IgG negative controls in parallel for each pair of primers.

### Study of calcium flux in monocytes induced by chemokines

To study calcium flux in monocyte subpopulations, we used Fura2 QBT calcium kit (Molecular Devices) according to the manufacturer recommendations. Briefly, Sorted monocyte subpopulations were plated in 96-Well Half-Area Plates Black (Fisher Scientific) at 200,000 cells per well to cover all the surface of the well. Monocytes were left in Hanks’ Balanced Salt solution at 37 °C for 20 min for adhesion before addition of an equal volume of reconstituted Fura2 loading solution. Cells were incubated at 37 °C for 1 h before performing calcium mobilisation assay on FlexStation instrument (Molecular Devices) as recommended by the manufacturer. For single induction with chemokines, the 4X concentrated chemokine solution was added after 16 s and for sequential double induction, the second chemokine was added after 60 s of run. The excitation wavelengths were 340 nm and 380 nm. The emission wavelength was 510 nm with a cutoff at 495 nm. The ratio of fluorescence (340/380 nm) was used to measure calcium mobilisation. The initial point was set at 1 by using the following formulae

2 $${\mathrm {Normalized}}\,{\mathrm {value}} = {\mathrm {ratio}}\left( t \right) - {\mathrm {initial}}\,{\mathrm {ratio}}\left( {t}{0} \right) + {1},$$

*t*: given time point and *t*0: initial time point

Chemokines was used at the concentration indicated in Supplementary Table 6. To investigate the role of CX3CR1 and other chemokine receptors in calcium mobilisation, monocytes were also incubated with dialysed anti-CX3CR1 antibody in combination or not with the pertussis toxin at concentrations indicated in figure legends.

### Validation of CX3CR1 functional blocking antibody

Based on the highly conservation of CX3CR1 between human, mouse and rat, we have tested the capacity of a neutralisation antibody commercially available (Genetext, GTX27200) and originally developed in rabbit against rat Cx3cr1. The antibody was dialysed in PBS for 24 h at 4 °C to remove Sodium Azide before use in the assays. The anti-CX3CR1 at 10 µg/ml was used as primary antibody for staining human monocytes. As negative control, we used same amount of rabbit IgG. The adequate secondary antibody was used to reveal the staining that was analysed by flow cytometry with an Accuri C6 instrument (BD Biosciences). Different concentration of anti-CX3CR1 were tested in calcium mobilisation assay (20, 40 and 50 µg/ml). Therefore, sorted monocytes were incubated with anti-CX3CR1 or IgG also during Fura2 loading at 37 °C.

### Stable knockdown and transient downregulation in HUVEC

To stably knockdown CX3CL1 expression in HUVEC, we transfected HUVEC either with 4 unique 29 mer short hairpin (sh) RNA-producing plasmids targeting human *CX3CL1* or non-effective 29 mer scrambled control shRNA (Ref: TG313636, Origene Technologies, Inc). Both plasmid types expressed kanamycin and puromycin for bacterial and eukaryotic selections, respectively, and GFP for well transfected cell sorting by FACS. Briefly, HUVEC were transfected with 10 µg of plasmids with Neon Transfection System (Thermo Fisher Scientific), and selected for their resistance to 500 ng/ml puromycin for 15 days. Highly expressing GFP clones were sorted by FACS with MoFlo Astrios cell sorter (Beckman Coulter). Efficiently transfected HUVECs were amplified and tested by qPCR and western blotting for CX3CL1 expression under quiescence and activation conditions. These transfected HUVEC were used to study angiogenic monocyte recruitment under flow. To study the role of GATA3 in the regulation of CX3CL1 expression, as well as HPMo transmigration under flow, HUVECs were transfected with small interference RNA (siRNA) targeting human *GATA3* or control siRNA (Santa Cruz Biotechnology) with Neon Transfection System (Thermo Fisher Scientific). GATA3 downregulation was confirmed by western blotting before further experiments with transfected cells.

### Stable overexpression of TIMP1 in DLD1 tumour cells

DLD1 tumour cells were transfected with either *TIMP1* or empty pCMV6-AC-GFP vectors (RG201548, Origene). Both vectors expressed ampicillin and neomycin resistance genes for bacterial and eukaryotic selections respectively and GFP for transfected cell sorting by FACS. Transfection was performed with 10 µg of plasmids with Neon Transfection System (Thermo Fisher Scientific). GFP + DLD1 cells were sorted by FACS and cultured under the selection with G418 (300 µg/ml), Geneticin (Thermo Fisher Scientific) for 2 weeks. TIMP1 overexpression was confirmed by quantitative PCR analysis.

### Tube formation and proliferation assays

To analyse endothelial cell sprouting and migration in co-cultures with monocyte subsets, endothelial tube formation assay on matrigel was used. Growth factor-reduced matrigel (BD Biosciences) (5 mg/ml) in simple M199 medium was thawed on ice and polymerised in 96-wells angiogenesis µ-plate (IBIDI) at 37 °C. Monocyte subsets were stained with Celltracker Green 5-chloromethylfluorescein diacetate CMFDA (Thermo Fisher Scientific), mixed with unlabelled human umbilical vein endothelial cells (HUVEC) and added to polymerised matrigel. The plates were imaged overtime for 10 h with motorised ImageXpress microscope (Molecular Devices). Image sequences were combined and analysed with ImageJ Angiogenesis Analyser toolset by quantifying total tube length (sum of all segments) and network stability index (ratio of total tube length/number of isolated segments). The monocyte subset with highest tube formation indices was considered as the most proangiogenic. The difference were considered significant and indicated on graphs only when *p*-value ≤0.05 after non-parametric Mann–Whitney tests.

To analyse endothelial cell proliferation induced by monocyte-secreted molecules, the fluorescent 5-ethynyl-2′-deoxyuridine (EdU) incorporation assay was used to monitor DNA synthesis with Click-iT Plus EdU Kit (Thermo Fisher Scientific) according to manufacturer recommendations. Briefly, human monocyte subsets were sorted and cultured in simple RPMI medium for 24 h and the conditioned media were collected and added (1/5 final ratio) to HUVECs in 96-wells plate in the presence of 10 µM EdU. Endothelial cells were cultured for 20 h and then fixed with 4% formaldehyde in PBS and permeabilized with 0.1% Triton X-100. EdU detection in this assay is based on a copper catalysed covalent reaction between an alkyne and a picolyl azide (click reaction). Thus, incorporated EdU containing the alkyne could then be detected by incubating with AF488-conjugated picolyl azide in the presence of copper. Total cell nuclei were stained with DAPI (4′,6-diamidino-2-phenylindole) and endothelial cell junctions with goat anti-VE-cadherin followed by a secondary DyLight-594- anti-goat IgG. Plate was imaged using ImageXpress (Molecular Devices) or Nikon Ar1 (Nikon) microscopes and the pictures analysed with Metamorph Analysis software (Molecular Devices). Data from 4 different fields per conditions were quantified per experiment and presented as a mean ± standard deviation as indicated in the figure legends. The difference were considered significant and indicated on graphs only when *p*-value ≤0.05 after non-parametric Mann–Whitney test or ANOVA Sidak adjustment.

### Signalling pathway exploration

To analyse endothelial cell signalling pathways, specific stimuli of HUVECs by applying angiogenic monocyte conditioned mediums were performed for 20 min. Endothelial cells were treated with either conditioned media, VEGF-A (100 ng/ml) or Aminophenyl mercuric acetate (APMA, 20 µM) unless specified otherwise. For experiments with inhibitors, endothelial cell monolayers were pre-treated for 15 min with the inhibitors before the stimulus addition, and stimulations were done in continuous presence of inhibitors. A list of inhibitors used in this study are presented in Supplementary Table 7. Endothelial cell monolayer were then washed with Ca^2+^ Mg^2+^ PBS, protein extracted and analysed by western blotting with specific antibodies. The same filters were used for different phospho-proteins and ACTINβ for total protein loading.

### Statistics and data presentation

Estimation of sample size in each experiments were determined with R software after preliminary experiment with at least 3 replicates per group. The calculations were performed with a minimal differences between compared groups, a standard deviation, a significance of 0.05 and a power of 0.8. The data were presented as mean ± standard deviation (s.d.), mean ± standard error of mean (s.e.m.), boxplot with whiskers and median or dot plot with median. The data presentation are specified in figure legends. Comparison between two experimental groups from independent experiments was done with Student’s *t*-test or Mann–Whitney non-parametric test as indicated in figure legends. This was done with Graphpad Prism 6. ANOVA test was performed with Holm-Sidak adjustment for multiple comparison. Multiple *t*-test was performed with Holm-Sidak adjustment without assuming consistent s.d. for different donors of monocytes. Mixed models also were performed for the effect of treatment on HPMo recruitment to xenografts by testing the treatment as fixed effect with a random effect of donors on intercept and treatment, thus taking into account the variability in average response between donors and the variability of treatment effect between donors. To investigate the association of molecule expression with ADIn or conventional inflammation, we performed mixed models testing the inflammatory condition as fixed effect with a random effect of experiments on intercept only. This analysis was performed by using R software. An effect was considered significant when *p*-value <0.05.

### Data availability

The data that support the findings of this study are available within the article and its Supplementary Information files and from the corresponding author upon reasonable request.

## Electronic supplementary material

Supplementary Information

Description of Additional Supplementary Files

Supplementary Movie 1

Supplementary Movie 2

Supplementary Movie 3

Supplementary Movie 4
