# Supplementary material for: Angiogenic factor-driven inflammation promotes extravasation of human proangiogenic monocytes to tumours
Source: Nat Commun. 2018 Jan 24;9:355. doi: 10.1038/s41467-017-02610-0 (PMC5783934; doi:10.1038/s41467-017-02610-0)
Supplement: Supplementary file 1 — Supplementary Information [file 41467_2017_2610_MOESM1_ESM.pdf]

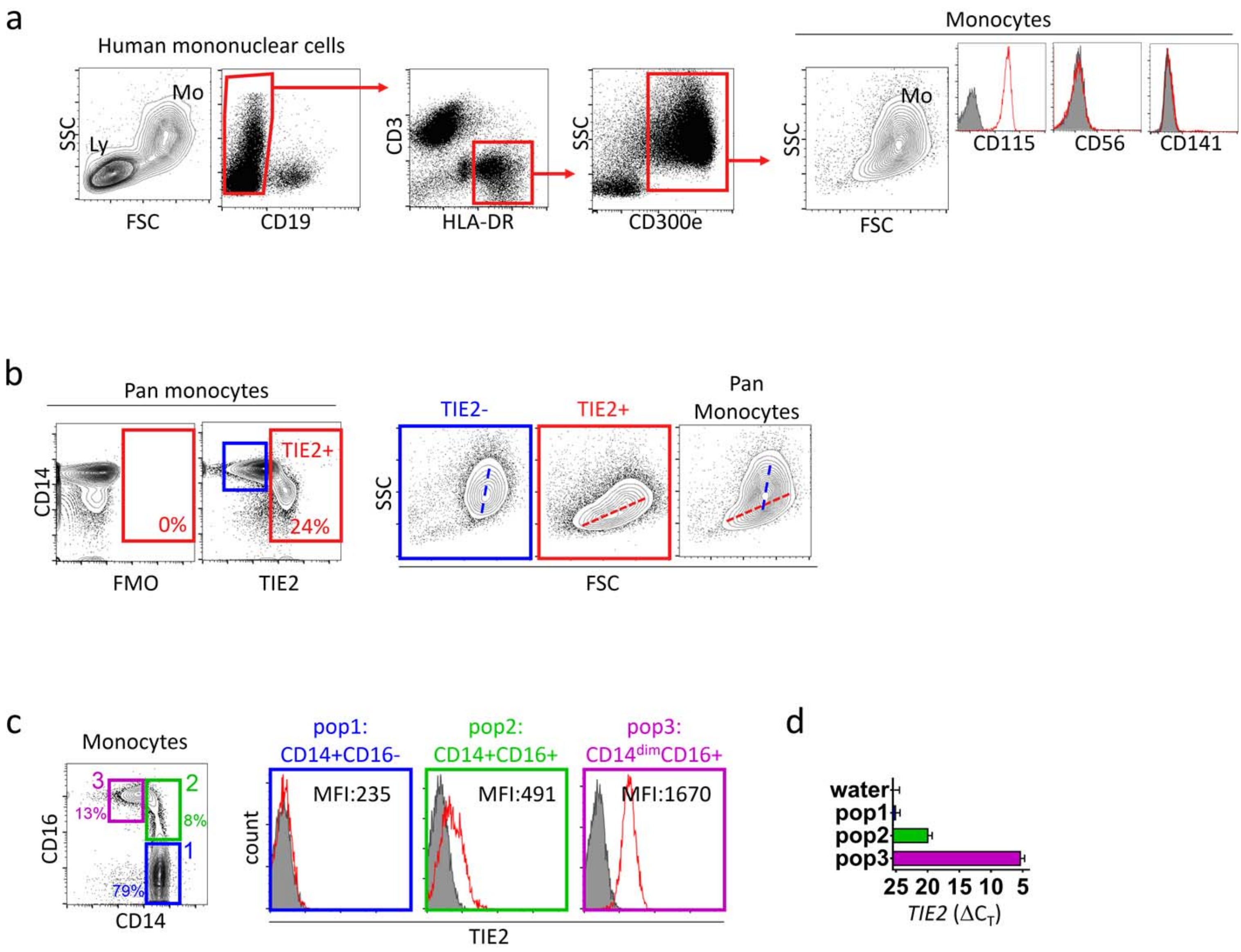

Supplementary figure 1: human non-classical monocytes express TIE2, a marker of angiogenic monocytes

a. Gating strategy used for flow cytometry of human monocytes within peripheral blood mononuclear cells. B cell (CD19+), T cells and some NK cells (CD3+) were depleted and monocytes were specifically gated with CD300e expression. Gated monocytes were CD115+ and negative for NK cell marker CD56 and dendritic cell marker CD141. b. FACS analysis of the morphology of TIE2+ monocytes compared to TIE2- ones and relatively to the pan monocytes. FMO (fluorescence minus one) staining condition was used as control for TIE2 specific staining. Blue and red dash lines indicates morphological spatial distribution of TIE2- and TIE2+ monocytes respectively. Human TIE2+ monocytes showed morphological differences from TIE2- cells. c. Analysis of TIE2 protein expression by human monocyte subpopulations based on CD14 and CD16. Inflammatory (CD14+CD16-), Intermediate (CD14+CD16+) and non-classical (CD14dimCD16+) are described as pop1, pop2 and pop3 respectively. Non-classical (CD14dimCD16+) monocytes showed the highest level of TIE2 whereas intermediate cells were slightly positive and inflammatory monocytes were negative. d. Quantification of relative *TIE2* mRNA levels (ΔCT) in human monocyte subpopulations by quantitative polymerase chain reaction (qPCR). Data are representative of at least 5 independent experiments

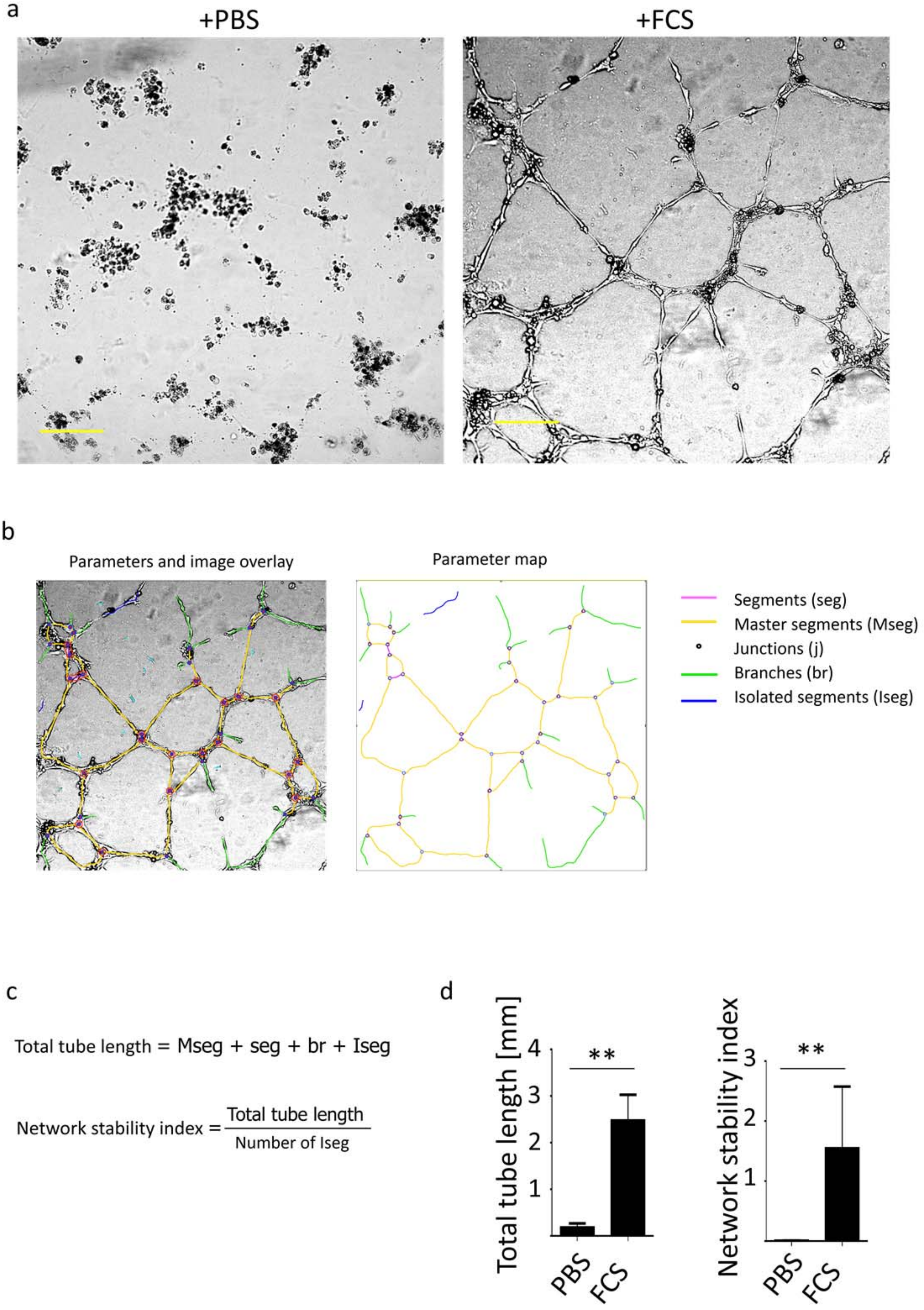

Supplementary figure 2: Endothelial tube formation assay and quantification

a. Pictures of endothelial cells in sprouting and tube formation combined assay under PBS or FCS challenge after 10 hrs. Pictures are representative of 5 replicates from 2 independent experiments In presence of FCS, endothelial cells sprout, migrate and form tube-like structure inherent to their

angiogenic activity whereas with PBS they remains singlets or form clumps but don't sprout nor migrate. Scale bar: 150 µm b. Tube-like structure quantification with ImageJ Angiogenesis Analyzer toolset. Different angiogenic parameters are measured from images of tubes including segment size, junction numbers, branch size and number, isolated segment size and number etc. c. Global measurement of the angiogenic activity by calculating the total tube length and the network stability index from angiogenic parameters. These two global measurements describe the sizes of the tubes and their respective quality. d. Example of quantification of endothelial sprouting and tube formation induced by FCS, compared to a PBS control. n=5 technical replicates per group, representative of 2 independent experiments, **:p-value<0.01 in Mann-Whitney test

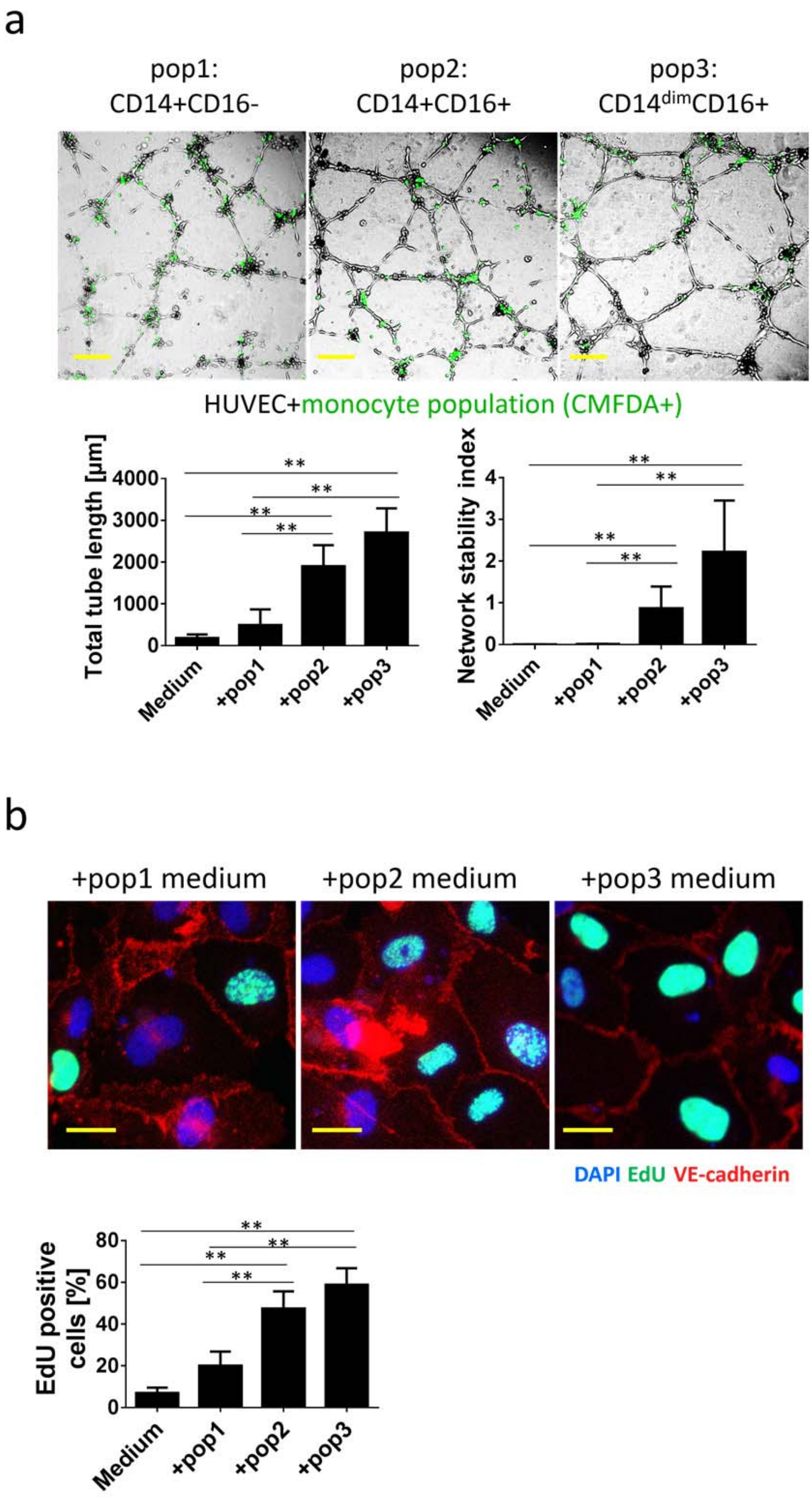

Supplementary figure 3: human non-classical and intermediate monocytes induce endothelial tube formation and proliferation

a. Endothelial cell sprouting and tube formation induced by co-culture with purified monocyte subpopulations. Monocytes were pre-stained with Celltracker™ green CMFDA (top panel), Scale bar=

100 μm; quantification of the tube length and the network stability index (bottom panel). b. Induction of endothelial cell proliferation by culturing with conditioned media derived from purified monocyte subsets assayed by EdU incorporation (top panel), Scale bar= 20 μm, and its quantification (lower panel). Data are presented as mean ± s.d. **: $p<0.01$ in Mann-Whitney test. Data are representative of at least 5 independent experiments with monocytes from different donors.

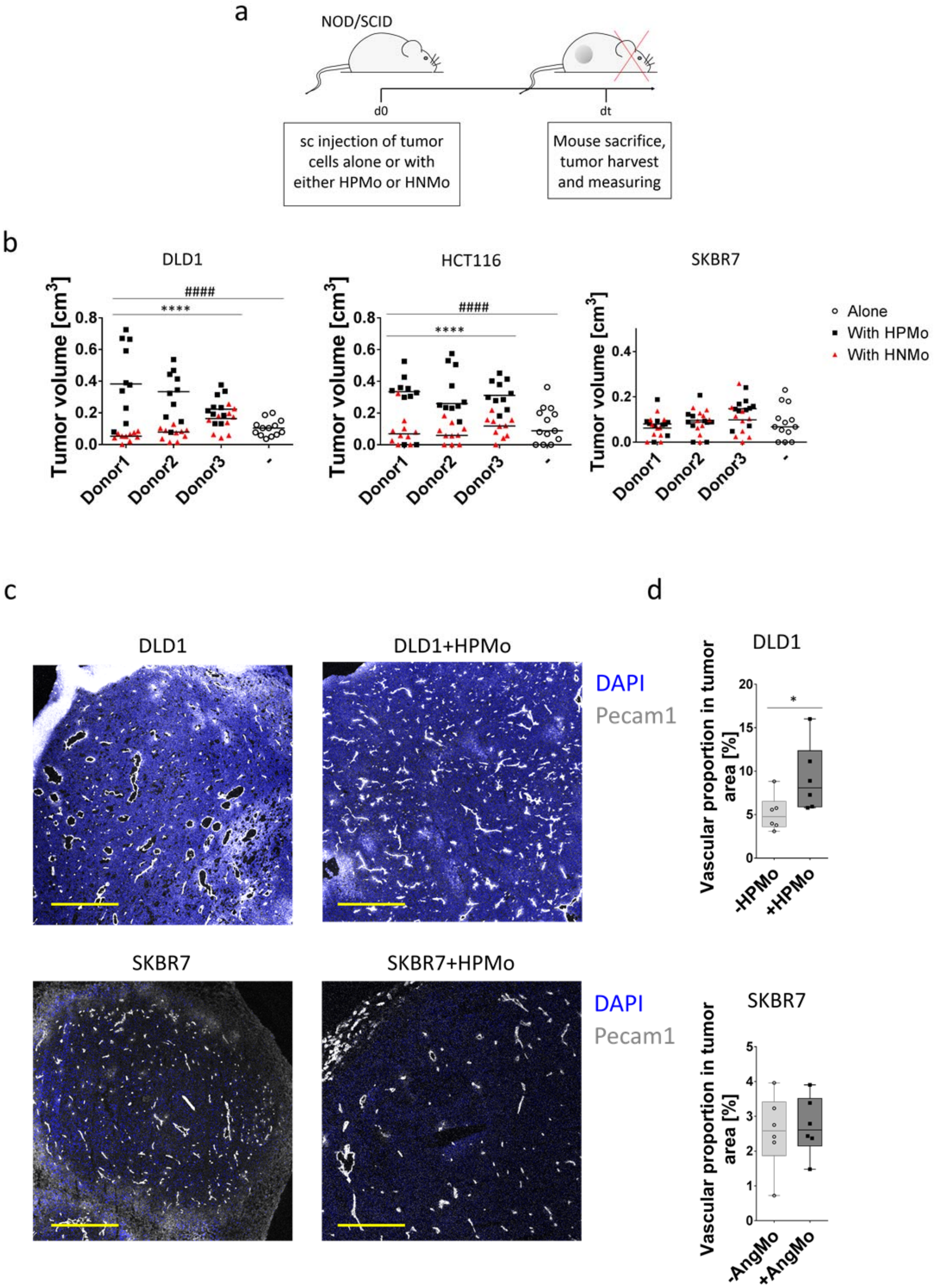

Supplementary figure 4: HPMo promote angiogenesis and growth of human tumour xenografts

a. Strategy of studying the protumorigenic capacity of human monocyte subsets to support the development of human tumour xenografts in NOD/SCID mice. Mice were subcutaneously injected with tumour cells (DLD1, HCT116 or SKBR7) alone or with one of human monocyte subsets. Mice were s.c injected every two days with human CSF-1 (0.5 mg/kg) [54]. Mice in all groups of the same tumour type were sacrificed when median of the condition of tumour cell alone reached 0.1 cm^{-3} in

volume. b. Effect of proangiogenic monocytes on the growth of different human tumour xenografts. HPMo from different donors boosted DLD1 and HCT116 xenograft growth but did not affect SKBR7 xenograft growth. c. Tumour vessel staining with Pecam1-specific antibody to evaluate the influence of HPMo co-injection on the vascular density of DLD1 and SKBR7 xenografts. The nuclei were stained with DAPI. Scale bar=200 µm. d. Quantification of the proportion of vascular areas of the conditions presented in (c). Data are presented as scatter dot plots with medians (horizontal lines) for tumour size plots in (b) or scatters with boxplot to show value range (Min-Max) in (d) *: $p<0.05$ in Mann-Whitney test; ****: $p<0.0001$ in mixed models testing monocyte subset effects with random effect of donor on HPMo or HNMo functions; ####: $p<0.0001$ in one-way ANOVA testing the effect of HPMo on tumour growth. n= 10 tumours from five mice per group in single experiment with monocytes from different donors (panel b) and n=6 tumours per group (5 sections from different plan quantified per tumour).

a

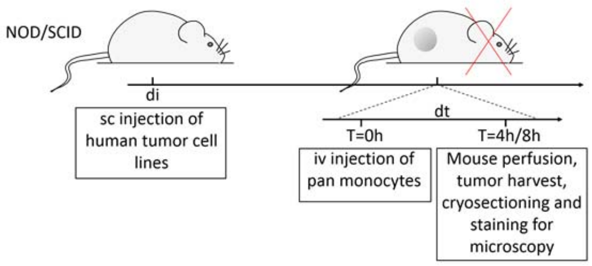

b

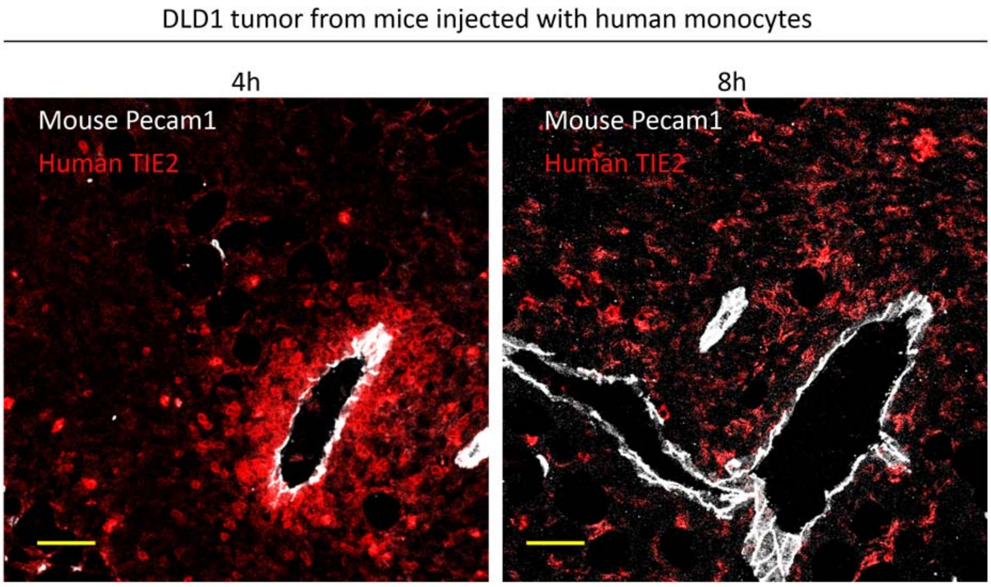

Supplementary figure 5: Distribution of human proangiogenic monocytes to tumour xenografts

a. Time course protocol of the assay of human monocyte recruitment to human tumour xenograft in vivo. Human tumour xenografts were injected subcutaneously at day di and let grow to reach 0.5-cm^3 of tumour volume. Mice were then i.v. injected at day dt with freshly isolated human monocytes. After 4-Hr or 8-Hr, mice were perfused with PBS-EDTA and sacrificed to harvest tumours that were then cryo-sectioned and the sections stained. b. Staining of sections of DLD1 tumours from mice injected with human monocytes. Sections were stained with antibody against mouse Pecam1 (Gray) to observe blood vessels and against human TIE2 (Red) to detect specifically the recruited angiogenic monocytes. After 4-h, monocytes were massively present around venules but after 8-Hr they were found spread throughout the tumours. Scale bar= 50 µm.

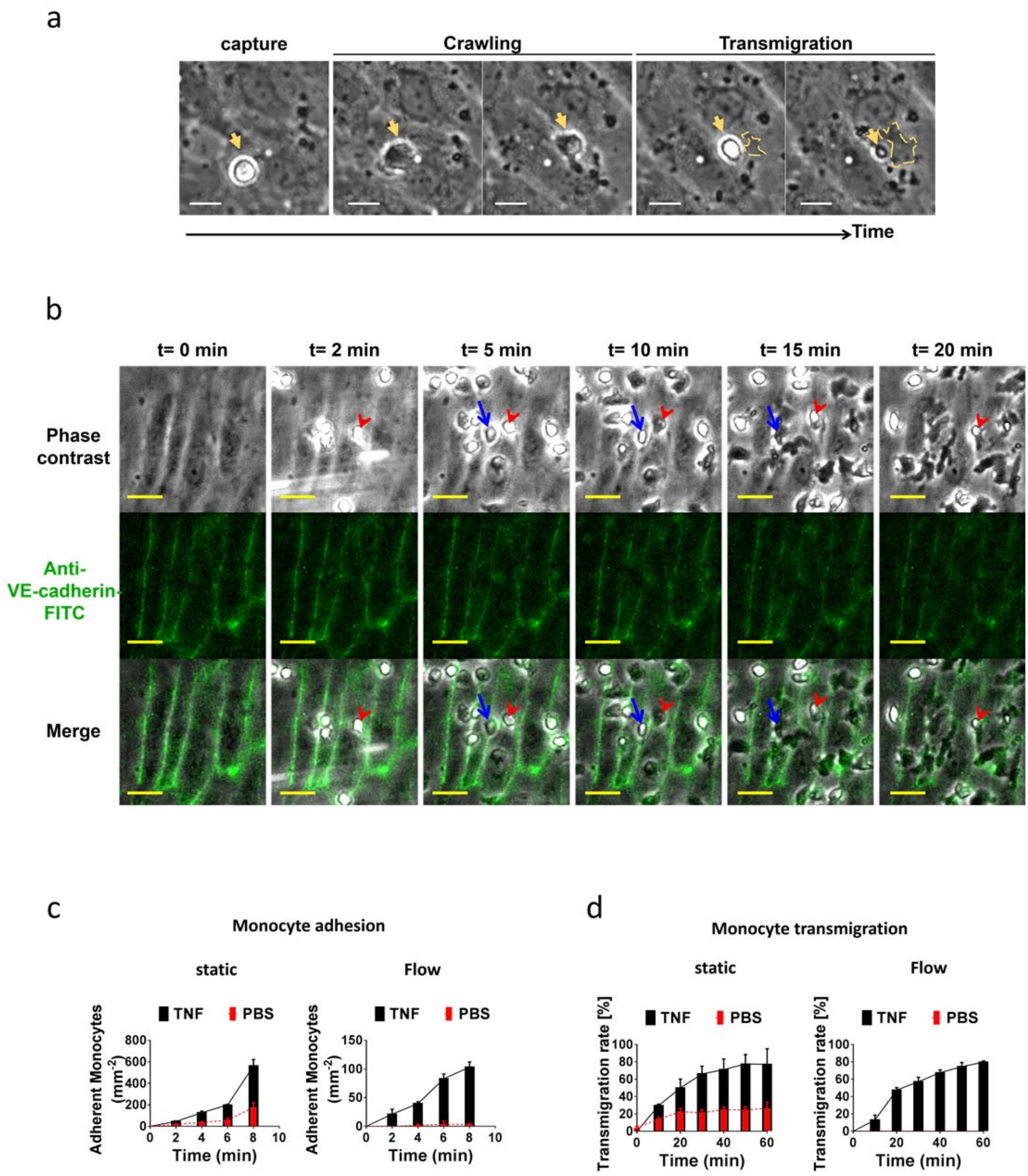

Supplementary figure 6: Flow is required for specific quantification of monocyte transmigration

a. Live imaging under flow allowed the combinatorial steps that define monocyte recruitment to be measured on individual monocytes. Yellow arrowhead indicate the position of a moving monocyte from capture to transmigration, the yellow dashed line delineates the cellular edges of the transmigrated monocyte under the endothelial monolayer. Scale bar= 5 µm. b. Composite Imaging of monocyte transendothelial migration under flow by combining phase contrast microscopy with fluorescent imaging of endothelial cell junctions, by using a non-blocking VE-cadherin antibody. Picture sequence is representative of 4 independent experiments. All monocytes exclusively transmigrated at endothelial intercellular junctions. The red arrowheads and blue arrows indicate two individual monocytes migrating toward junctions and transmigrating. Yellow scale bar= 10 µm. c. Comparison of monocyte adhesion to TNF-activated or non-activated (PBS) endothelial monolayer under static versus flow conditions. Under static condition up to 30% of adherent monocytes are

independent of endothelial cell activation status whereas monocytes adhere at very low levels to non-activated endothelial monolayer under flow. d. Comparison of monocyte transendothelial migration through TNF-activated or non-activated endothelial monolayer under static versus flow conditions. Under static condition up to 20% of adherent monocytes transmigrate through non-activated endothelial monolayer whereas under flow condition none of the sporadically adherent monocytes transmigrated through non-activated endothelial monolayers. This result shows the need of flow for robust quantification of human monocyte transmigration. n=3 technical replicates per group, representative of 3 independent experiments for panels c-d, data are presented as mean ± s.d for c-d.

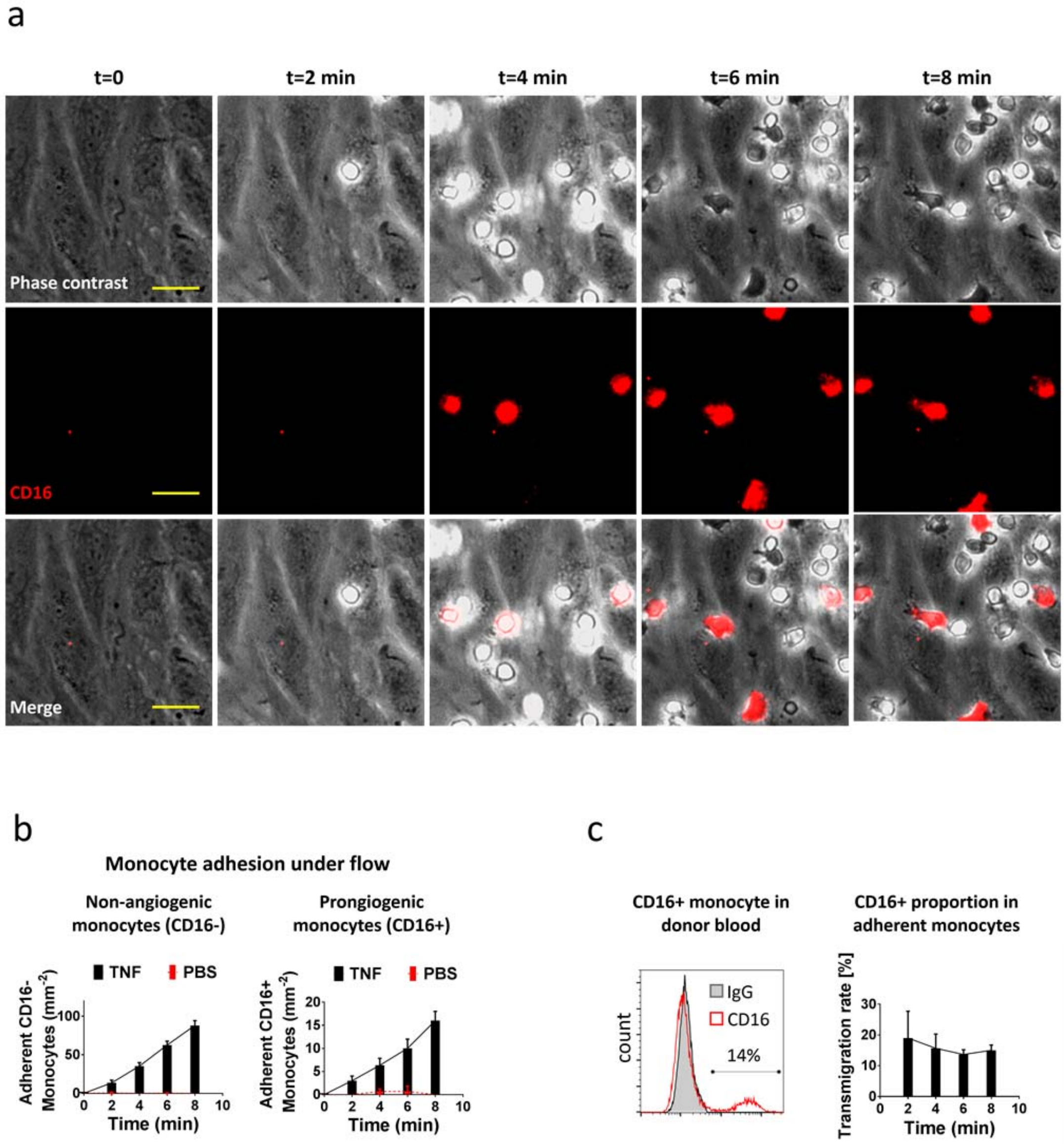

Supplementary figure 7: Proangiogenic monocytes adhere to TNF-inflamed endothelial monolayer

a. Live imaging of proangiogenic and inflammatory monocyte populations adhering to TNF-activated endothelial monolayer under flow. Proangiogenic monocytes are identifiable by CD16 staining with a non-blocking antibody to CD16. Scale bar (Yellow) = 10 µm. b. Quantification of the kinetics of proangiogenic versus non-angiogenic monocyte adhesion to TNF-activated and quiescent endothelial monolayer. Both non-angiogenic and proangiogenic monocytes adhere to TNF-activated endothelial monolayer overtime. c. Analysis of proangiogenic monocyte proportion in donor blood by cytometry (left panel) and within adherent monocytes overtime from live imaging result (right panel). Proangiogenic and non-angiogenic monocyte proportions are conserved between blood and on endothelial monolayer surface during adhesion assay under flow. n=3 technical replicates per group, representative of 3 independent experiments, data are presented as mean ± s.d. comparison of CD16+ cell percentage at each time point with a theoretical value of 14% (percentage in donor blood) by Wilcoxon Signed rank test was not significant ($p>0.75$) for each time point.

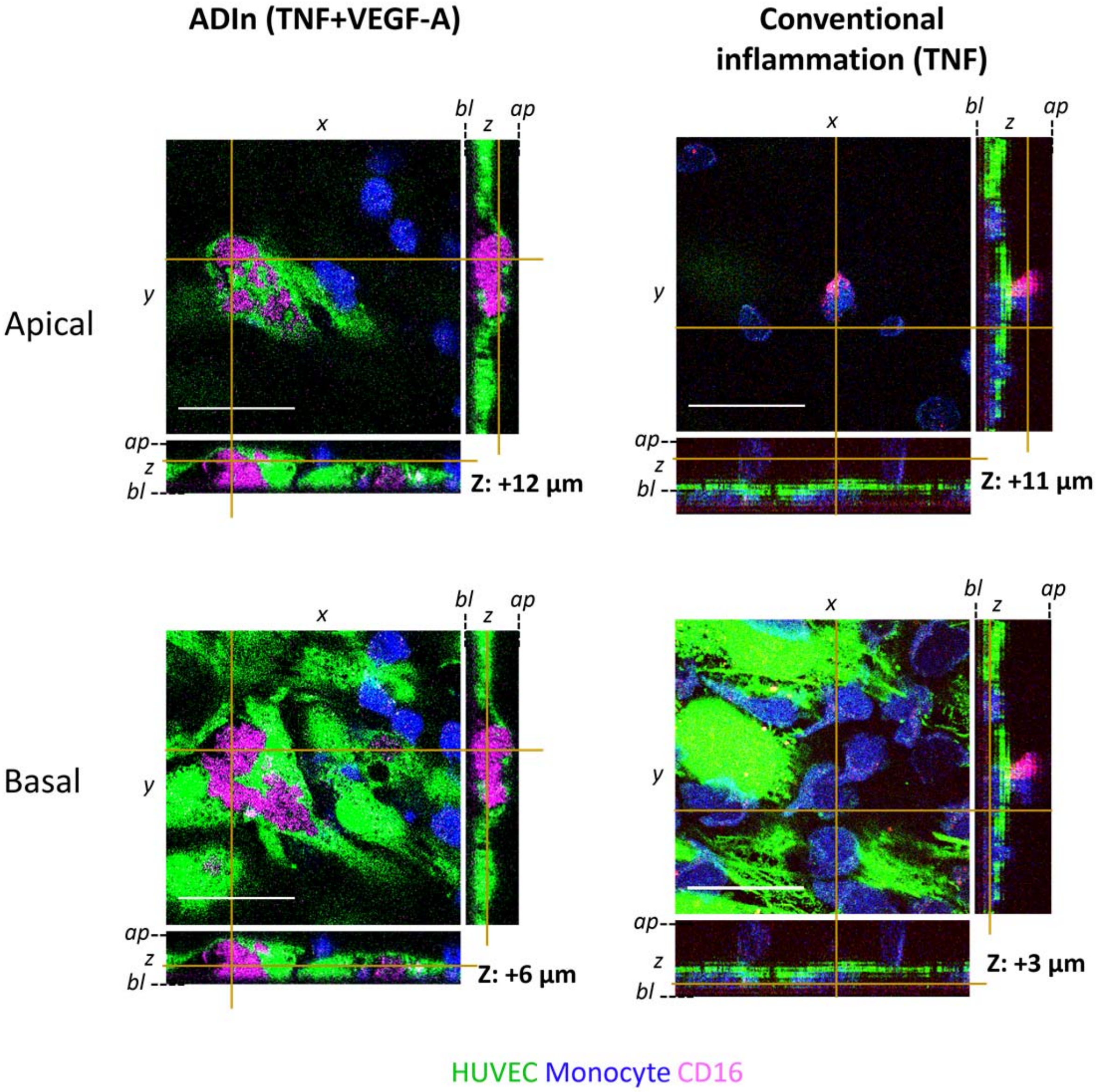

Supplementary figure 8: confocal imaging of human proangiogenic monocyte transmigration in ADIn

Post-transmigration assay analysis of proangiogenic and non-angiogenic monocyte positioning by confocal imaging. Scale bar= 10 µm, orthogonal presentation shows views from x, y and z axes. Basal (*bl*) and apical (*ap*) extremities are indicated. HUVEC and purified monocytes were pre-stained respectively with green and blue cell tracker prior to experimentation, and proangiogenic monocytes were stained with an anti-CD16 antibody after the transmigration assay was performed. Orthogonal views show transmigrated proangiogenic monocytes underneath endothelium when ADIn conditions were used. Data are representative of 3 independent experiments.

a

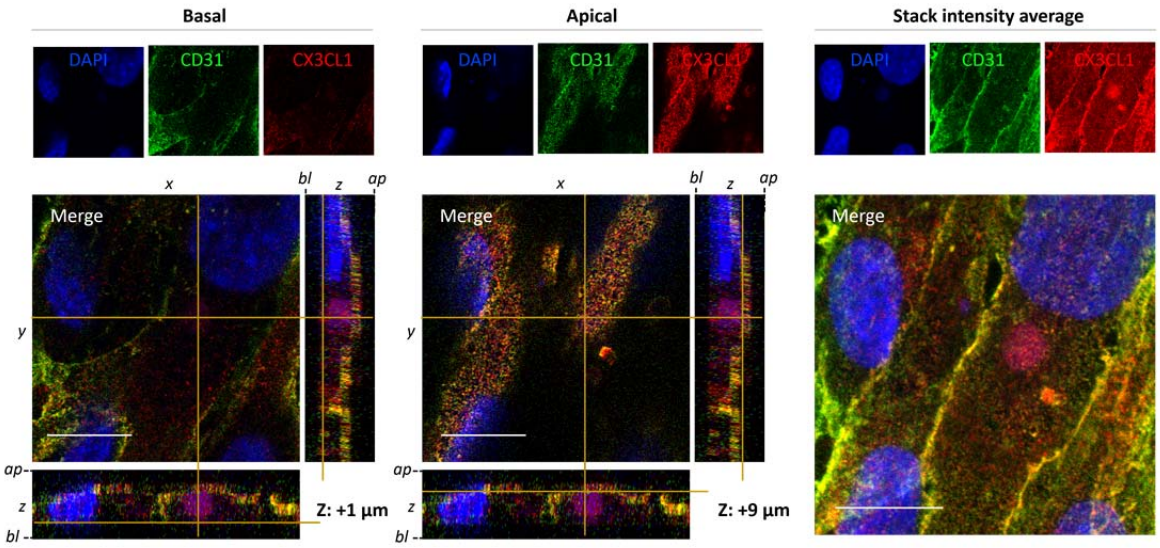

b

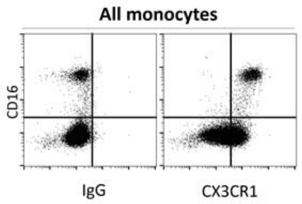

c

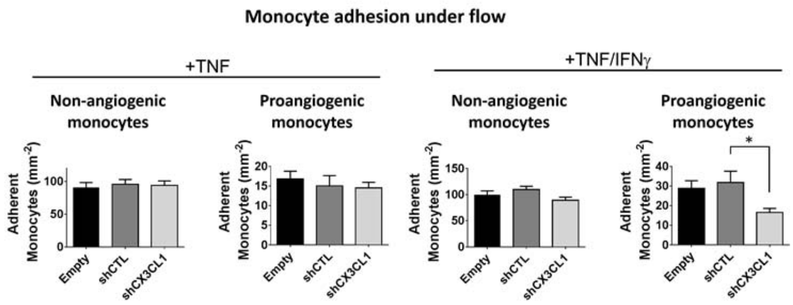

Supplementary figure 9: CX3CL1 and CX3CR1 expression on endothelial cells and monocytes respectively: Effect on adhesion of monocyte subpopulations

a. Analysis of CX3CL1 localization in endothelial cells by confocal imaging of nuclei with DAPI (Blue), CX3CL1 (red) and CD31 (green). Scale bar= 10 µm. The precise z position is indicated: Basal was at z=+1 and apical was at z=+9 µm, data are representative of 4 independent experiments, b. Analysis of CX3CR1 expression in proangiogenic (CD16+) versus non-angiogenic (CD16-) monocytes by flow cytometry, c. Analysis of CX3CL1 involvement in adhesion of monocyte subsets to TNF or TNF/IFNγ-activated HUVEC under flow. n=4 independent experiments with monocytes from 4 different donors, data are presented as mean ± s.e.m, *: $p<0.05$ in ANOVA-test.

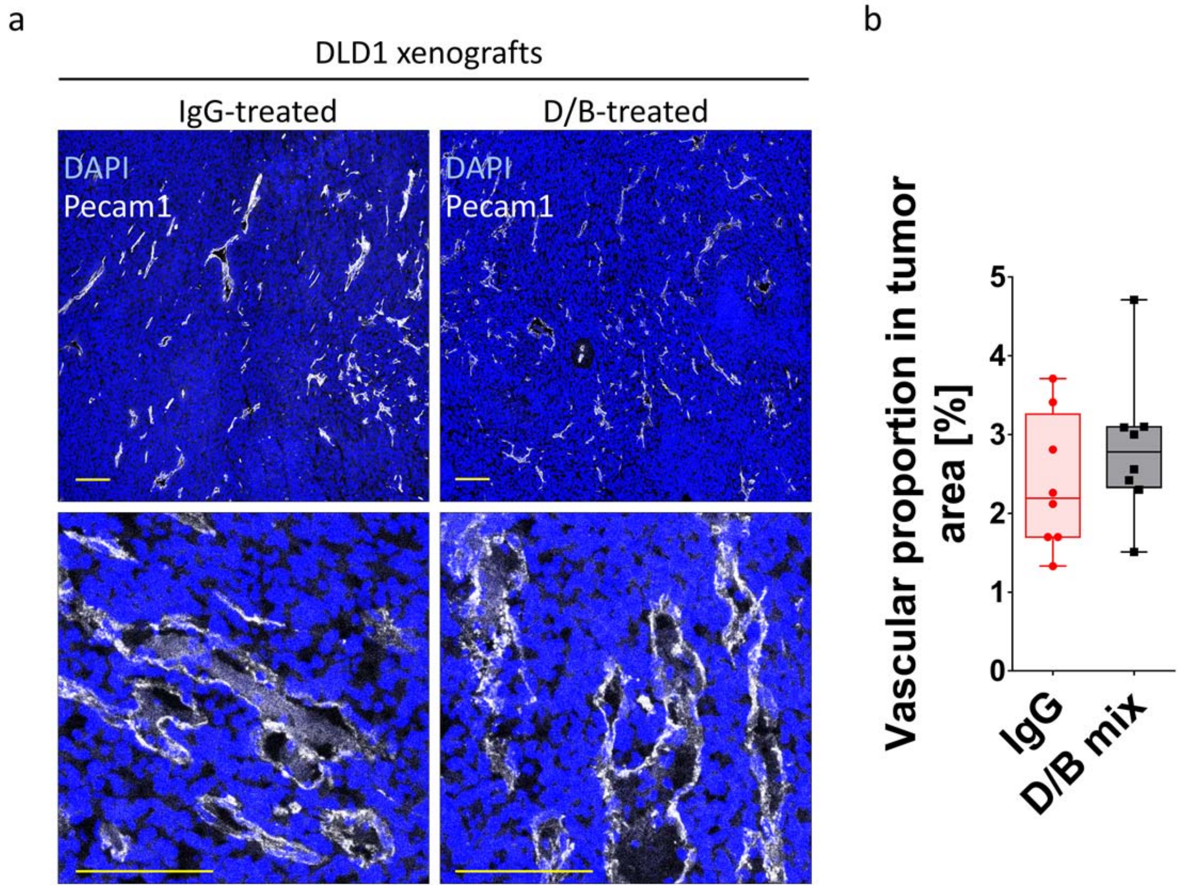

Supplementary figure 10: 24-Hrs treatment of human tumour xenografts-bearing mice with D/B mix did not affect tumour vascular density.

a. Analysis of the vascular density of DLD1 xenograft after 24-Hrs of treatment with the mixture of DC101 and bevacizumab by immunofluorescent staining of Pecam1 (gray) and the nuclei with DAPI (blue). Low (top) and high (bottom) magnifications are shown. Bar scale is 100 µm. b. Quantification of vascular density as proportion of tumour area analysed for 8 tumours per group. Data are presented as scatter dot plots and boxplot with median and range (Min-Max). Each dot represents one tumour and is the percentage of vascular area determined with five sections from different depth of the same tumour. Vascular area in tumours and total tumour areas were determined with ImageJ. Difference between the two groups was not significant (Mann-Whitney test).

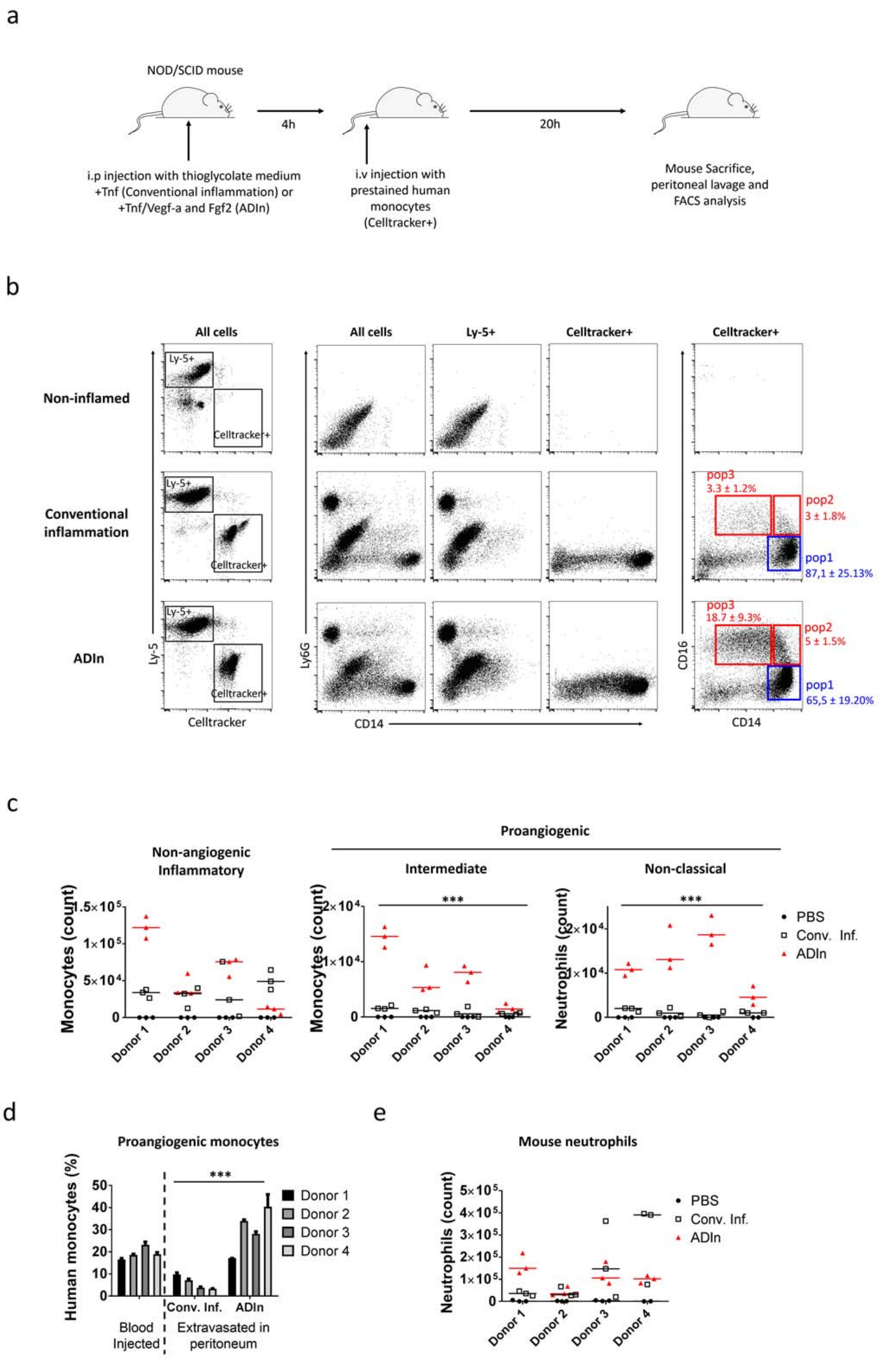

Supplementary figure 11: proangiogenic monocyte extravasation in non-tumour inflammation

a. Time course protocol of thioglycolate-induced peritonitis with conventional or angiogenic factor-driven inflammation (ADIn) in NOD/SCID mice. b. FACS analysis of leukocyte extravasation in mouse

peritoneum in indicated inflammatory conditions. Staining with an anti-Ly-5 (mouse Leukocyte common antigen CD45) antibody allowed distinction between mouse leukocyte population and engrafted human monocytes (celltracker+ cells). Both conventional inflammation and ADIn led to mouse neutrophil (Ly6G+) and human monocyte accumulation in the peritoneum. ADIn presented substantially higher frequencies of proangiogenic monocytes in the peritoneum. Increased numbers of both intermediate CD14+CD16+ (pop2) and non-classical or patrolling CD14dimCD16+ (pop3) monocyte accumulation were confirmed under ADIn conditions, when compared to conventional inflammation. Red gates indicate proangiogenic monocyte subsets including intermediate (pop2) and non-classical monocytes (pop3) whereas blue gates indicate non-angiogenic or inflammatory monocytes (pop1). Representative of independent adoptive transfer of pan-monocytes from 4 different donors, c. Actual cell counts of extravasated human monocyte subpopulations accumulated in the different inflammatory conditions. d. Percentage of human proangiogenic monocytes injected from donor blood and extravasated in mouse peritoneum in conventional Inflammation versus ADIn. e. Extravasated mouse neutrophil count in tested inflammatory conditions. For panels (c) to (e), n=3 mice per group in independent adoptive transfer of pan-monocytes from 4 donors, Data are presented as scatter dot plots with median (horizontal lines) for cell count plots and mean ± s.e.m for proangiogenic monocyte percentage plots. Black stars (***) indicate statistical significance ($p < 0.001$) in mixed models testing inflammatory conditions and considering a random effect of donors on intercept and inflammatory conditions.

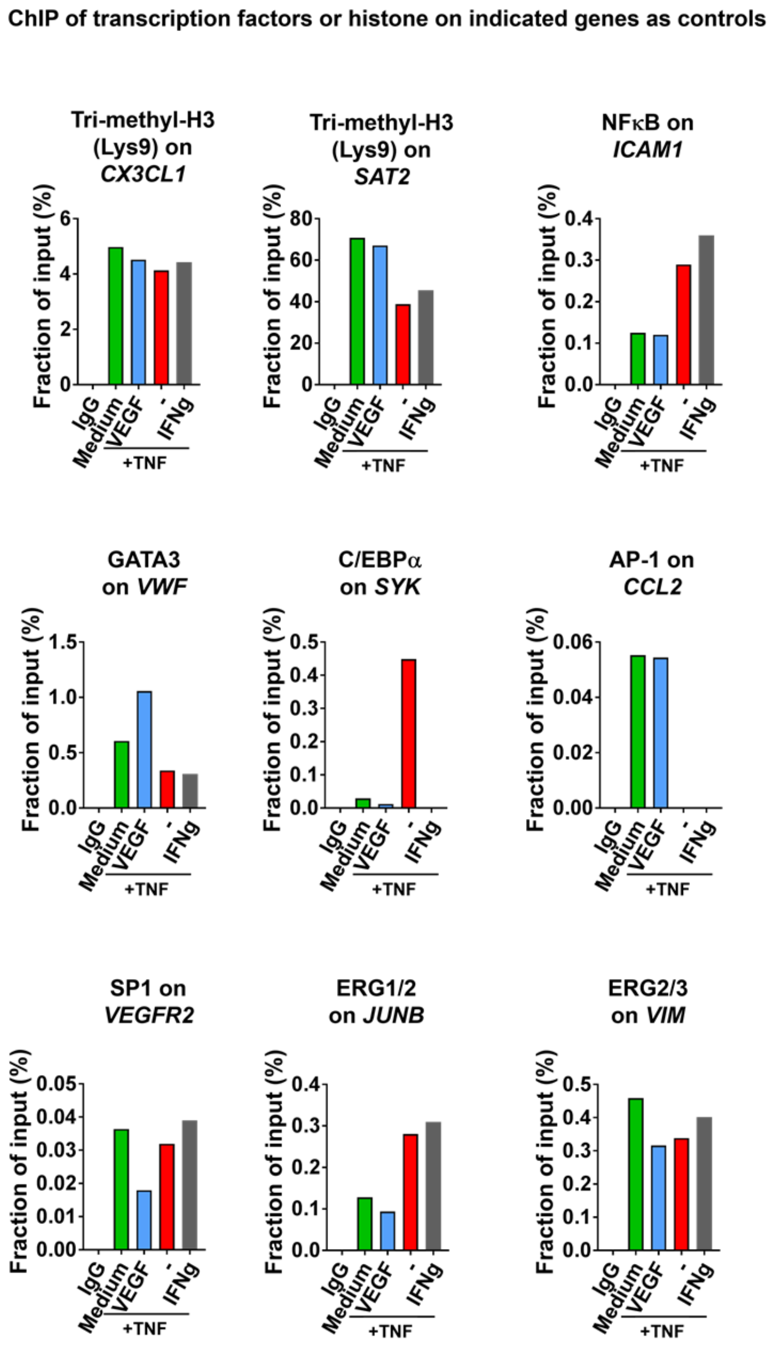

Supplementary figure 12: Analysis of control genes after chromatin immunoprecipitation (ChIP) of transcription factors and trimethyl-histone H3

Transcription factors including NFkB, AP-1, GATA3, C/EBPα, ERG1/2/3 as well as tri-methyl-H3 (Lys9) histone were immunoprecipitated and the bound DNA fragments were analysed by qPCR. Primers specific to the indicated genes were used to confirm the functionality of the ChIP assays. ChIP reactions were normalized with the input DNA. SAT2 serves as positive control for histone methylation, the *ICAM1* gene for NFkB, *CCL2* for AP-1, *von Willebrand factor* (*VWF*) for GATA3, *SYK* for C/EBPα, *JUNB* for ERG1 and 2, and *VIMENTIN* (*VIM*) for ERG2 and 3. Control genes were enriched with the transcription factors-targeting antibodies compared to IgG. Data are presented as mean of 3 technical replicates representative of 2 independent experiments.

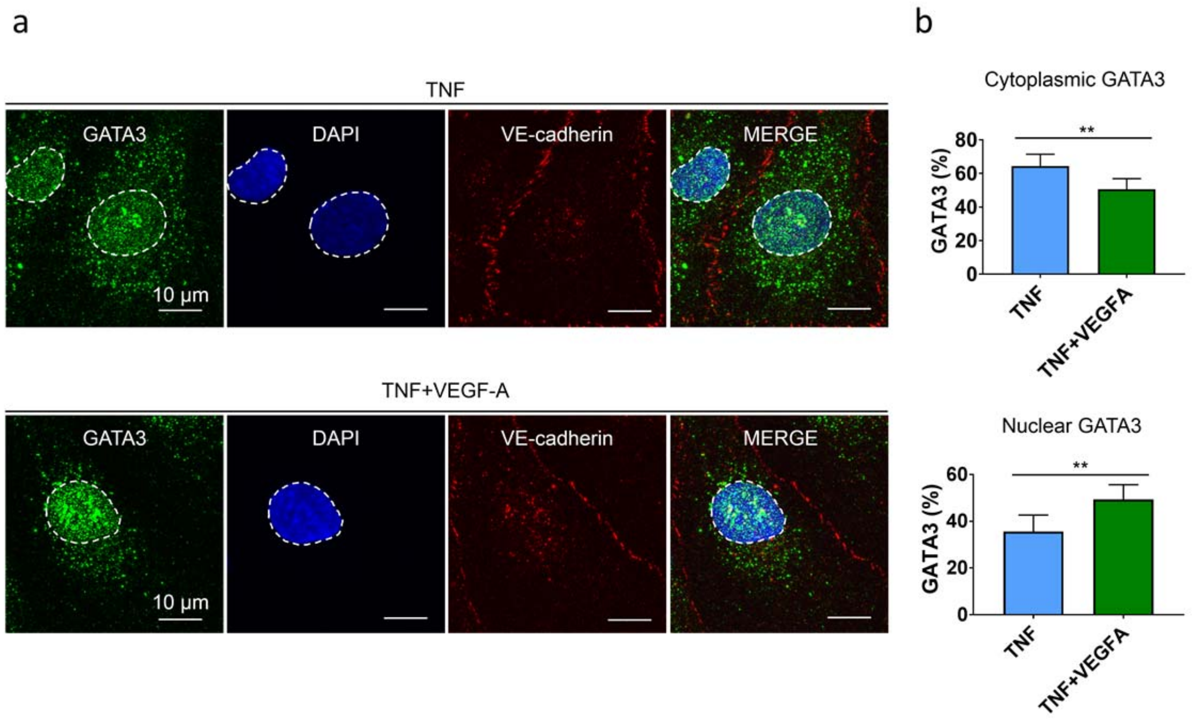

Supplementary figure 13: GATA3 translocation to the nucleus of endothelial cells under ADIn

a. Analysis of GATA3 distribution in HUVEC stimulated with TNF or TNF+VEGF-A by confocal microscopy. GATA3 (green), DAPI (blue) and VE-cadherin (red) are indicated. Images correspond to the maximum intensity of the stacks in each channel as well as merges. Scale bar=10 µm, dashed circles delimitate the nuclear regions, b. Quantification of GATA3 distribution between the cytoplasm and the nucleus is shown in percentage. The major part of GATA3 is cytoplasmic under TNF treatment and nuclear under TNF+VEGF-A challenge. Data are presented as mean ± s.d, n=6 experimental replicates pooled from 2 experiments.**:p<0.01 in t-test.

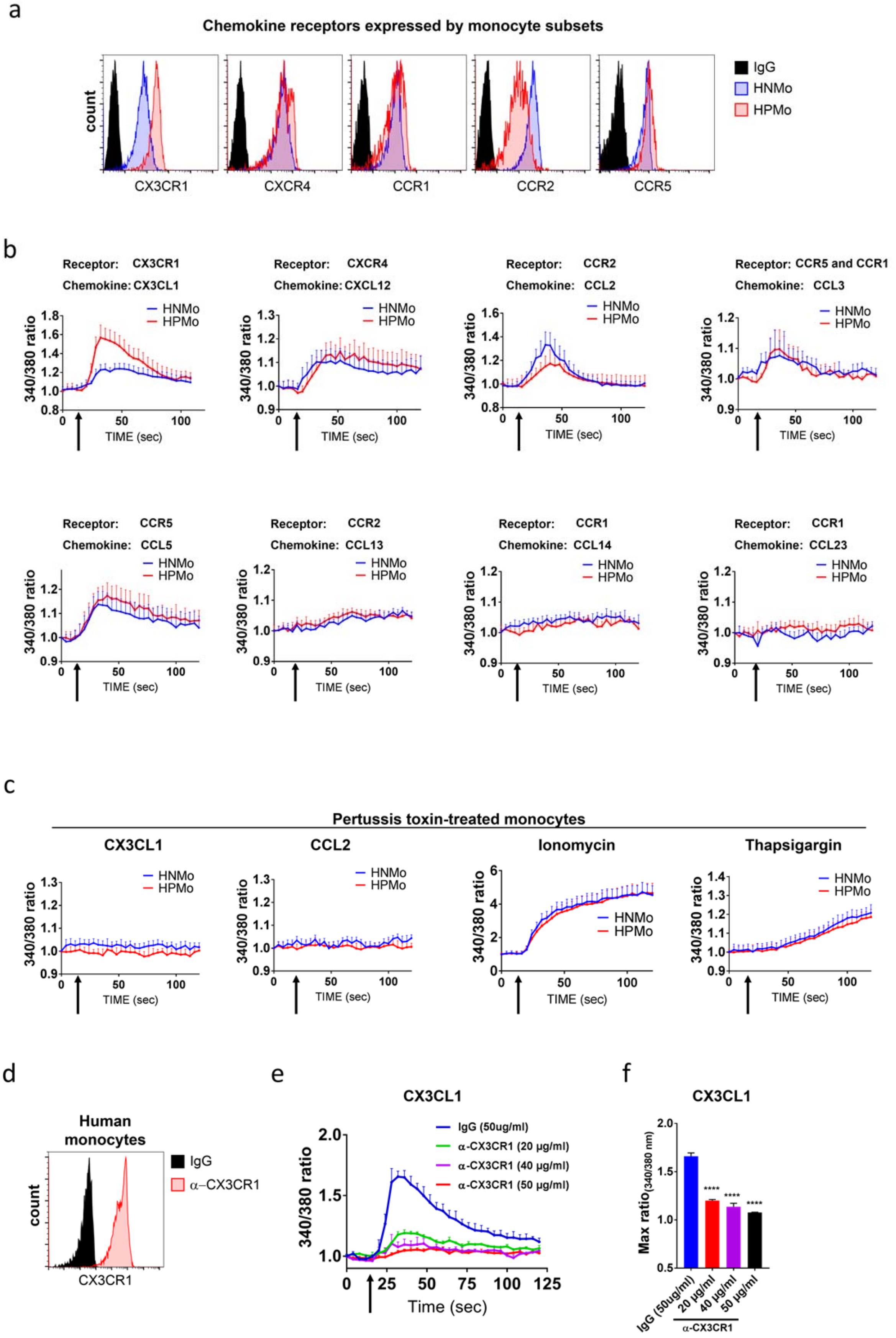

Supplementary figure 14: Chemokines-induced calcium flux in human monocyte subpopulations

a. Analysis of chemokine receptors expressed by monocyte subpopulations by flow cytometry, total monocytes incubated with IgG was used as negative control. The expression level of CX3CR1 was higher in HPMo compared to HNMo whereas CCR2 was higher in HNMo. The two subpopulations presented similar levels of the other chemokine receptors. b. Kinetics of chemokines-induced calcium flux in monocyte subpopulations. Fura2 QBT ratiometric assay was used to study the action of individual chemokine on calcium flux in isolated HPMo and HNMo. Black arrows indicate the time of chemokine addition. The chemokines were tested at 50 ng/ml. All these chemokines present at least one receptor on monocytes subsets. The action of CX3CL1 showed a stronger effect on HPMo compared to HNMo in contrast to CCL2 that showed a stronger action on HNMo. Their patterns were similar for the other chemokines. Data are presented as mean ± s.d. and made of 3 technical replicates, representative of 4 similar experiments, c. Effect of the inhibition of chemokine receptors on CX3CL1- and CCL2-induced calcium flux. The ionophore ionomycin (10 nM) and thapsigargin (10 nM), the inhibitor of calcium ATPase in endoplasmic reticulum were used as positive controls. The use of pertussis toxin (PTX), a potent inhibitor of G protein coupled receptors at 1 µg/ml for 1h before assays, inhibited CX3CL1- and CCL2-induced calcium flux. Calcium flux could still be induced by ionomycin and thapsigargin in presence of PTX. d. Dialyzed rabbit polyclonal anti-CX3CR1 (α-CX3CR1) could specifically detect human CX3CR1 by flow cytometry. e. Validation of the rabbit polyclonal anti-CX3CR1 as a functional blocking antibody against human CX3CR1. This commercially available antibody (GTX27200) was initially developed as neutralizing antibody against rat Cx3cr1. It was dialyzed in PBS before functional assays. Monocytes were incubated with anti-CX3CR1 at different concentrations as indicated or rabbit IgG at 50 µg/ml before calcium flux assay. The anti-CX3CR1 inhibited CX3CL1-induced calcium flux in a dose-depend manner. f. Comparison of maximal values (Max ratio from the data presented in panel c) of calcium flux in anti-CX3CR1- or IgG-treated monocytes. Data are presented as mean ± s.e.m of 3 technical replicates representative of 2 experiments. ****:p<0.0001 each concentration of anti-CX3CR1 compared to IgG control by ANOVA.

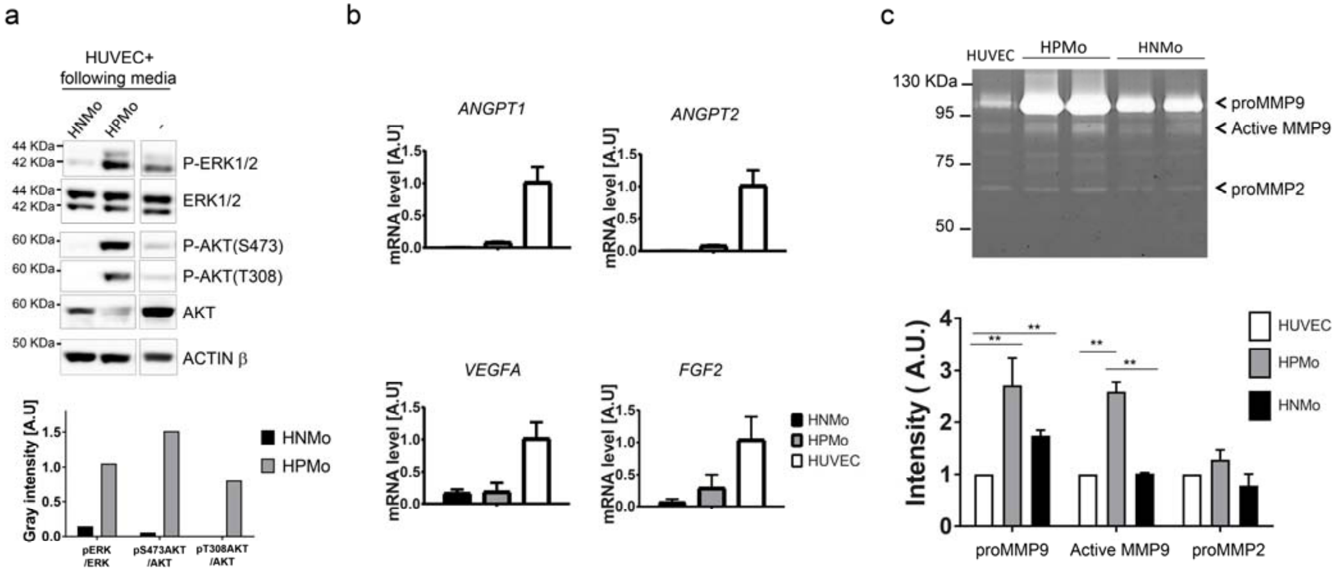

Supplementary figure 15: human proangiogenic monocytes secrete high level of active MMP9

a. Effect of monocyte subset-conditioned media on endothelial cell activation of MAPK and PI3K pathways (upper panel) and quantification (lower panel). Proangiogenic monocytes (HPMo)-conditioned media induced ERK and AKT phosphorylation at higher levels than non-angiogenic population (HNMo). The blots were cropped to remove an irrelevant lane, Figure with all lanes can be seen in the supplementary Fig. 16 b. Analysis of angiogenic factor expression by monocyte subsets by real time quantitative PCR. Endothelial cells express higher level of angiogenic factors than all monocytes. c. Zymography of metalloproteinases present in monocyte subsets-conditioned media (top panel) and the relative quantification of different zymograms (bottom panel). All the measures were presented as fold the protease amount expressed by HUVECs. Data are mean ± s.d, n=3 biological replicates from different donors. Both proangiogenic and non-angiogenic monocytes secrete higher levels of proMMP9 than HUVEC and proangiogenic monocytes exhibit more of the active form of MMP9 than either endothelial cells, or Non-angiogenic monocytes (**: $p<0.001$ in multiple t-test). Multiple t-tests were performed without assuming a consistent S.D and with Holm-Sidak adjustment.

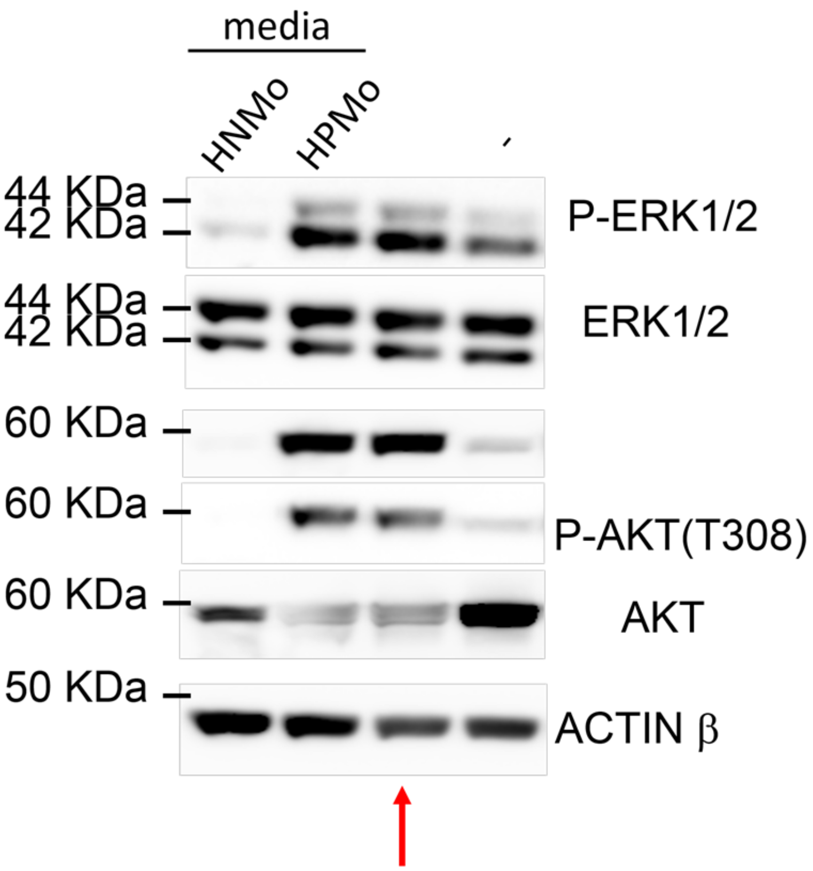

Supplementary figure 16: All lanes of the blots presented in figure 8a

In supplementary figure 15a, the blots were cropped to remove irrelevant lane indicated by red arrow.

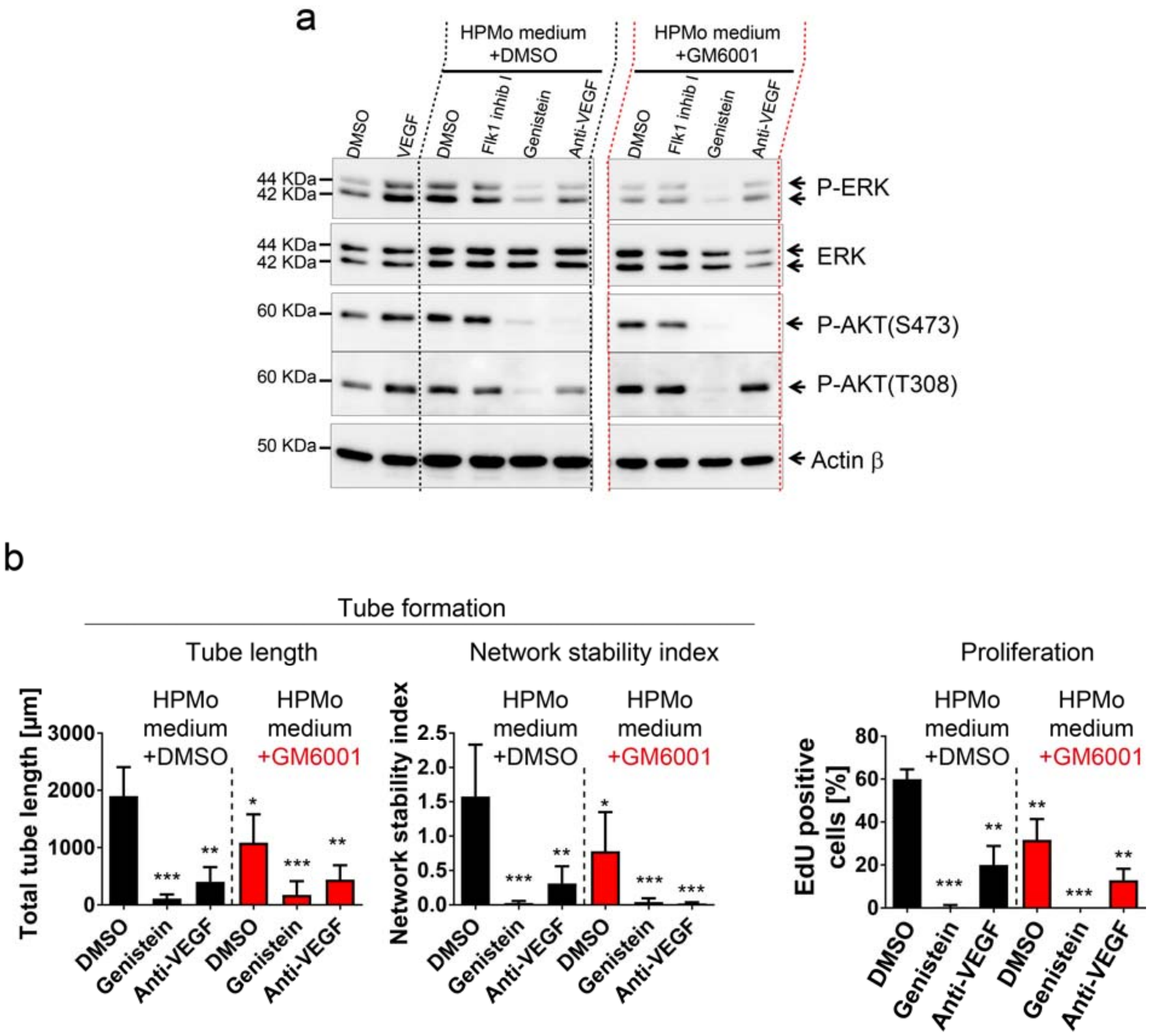

Supplementary figure 17: Active MMP9 secreted by HPMo mobilizes VEGF-A from extracellular matrix required for cell proliferation and tube formation in vitro

a. Participation of MMPs in MAPK activation induced by proangiogenic monocytes-conditioned medium. Data are representative of 4 independent experiments. Combination of MMP inhibitor and VEGF-A-specific antibody strongly decreases ERK and AKT phosphorylation. b. MMP and VEGF-A involvement in endothelial tube formation (left panel, n=5 independent experiment with monocytes from 5 different donors) and cell proliferation-induced by proangiogenic monocytes-conditioned medium (right panel). Combination of MMP inhibitor and VEGF-A-specific antibody (bevacizumab at 50 µg/ml) strongly impaired HPMo-induced endothelial tube formation and proliferation. Data are presented as mean ± s.d. *: $p<0.05$, **:$p<0.001$, ***:$p<0.0001$ in ANOVA with Sidak adjustment for multiple comparison.

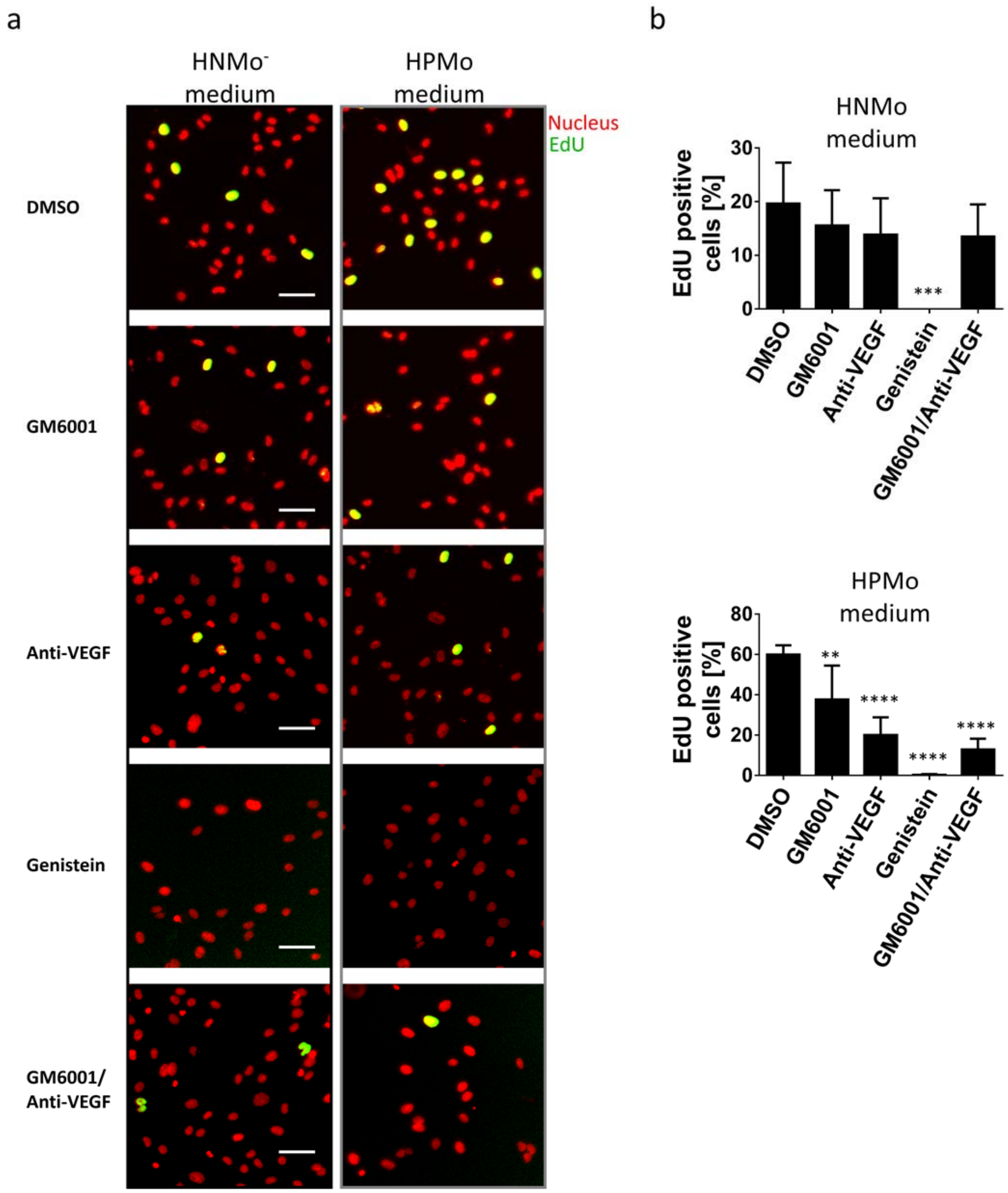

Supplementary figure 18: Medium of human proangiogenic monocytes induces endothelial cell proliferation through MMP and growth factor release

a. Effect of MMP inhibition and VEGF depletion on endothelial cell proliferation induced by the media of proangiogenic or non-angiogenic monocytes and quantified by EdU incorporation assay. EdU positive (green) nuclei were normalized with the total nuclei stained with DAPI (recolored in red on the pictures). b. Quantification of the effect of indicated inhibitors on endothelial cell proliferation induced by the media of monocyte subsets. For each subset medium, the inhibitor effects were compared to control DMSO condition. RTK inhibition with genistein completely inhibited endothelial proliferation induced by monocyte media. MMP inhibition combined with VEGF-A depletion using GM6001 and bevacizumab respectively, significantly decreased endothelial cell proliferation. n=5 independent experiments with monocytes from 5 different donors, data are mean ± s.d, **:p<0.01, ***:p<0.001, ****:p<0.0001 in ANOVA test with SIdak adjustment

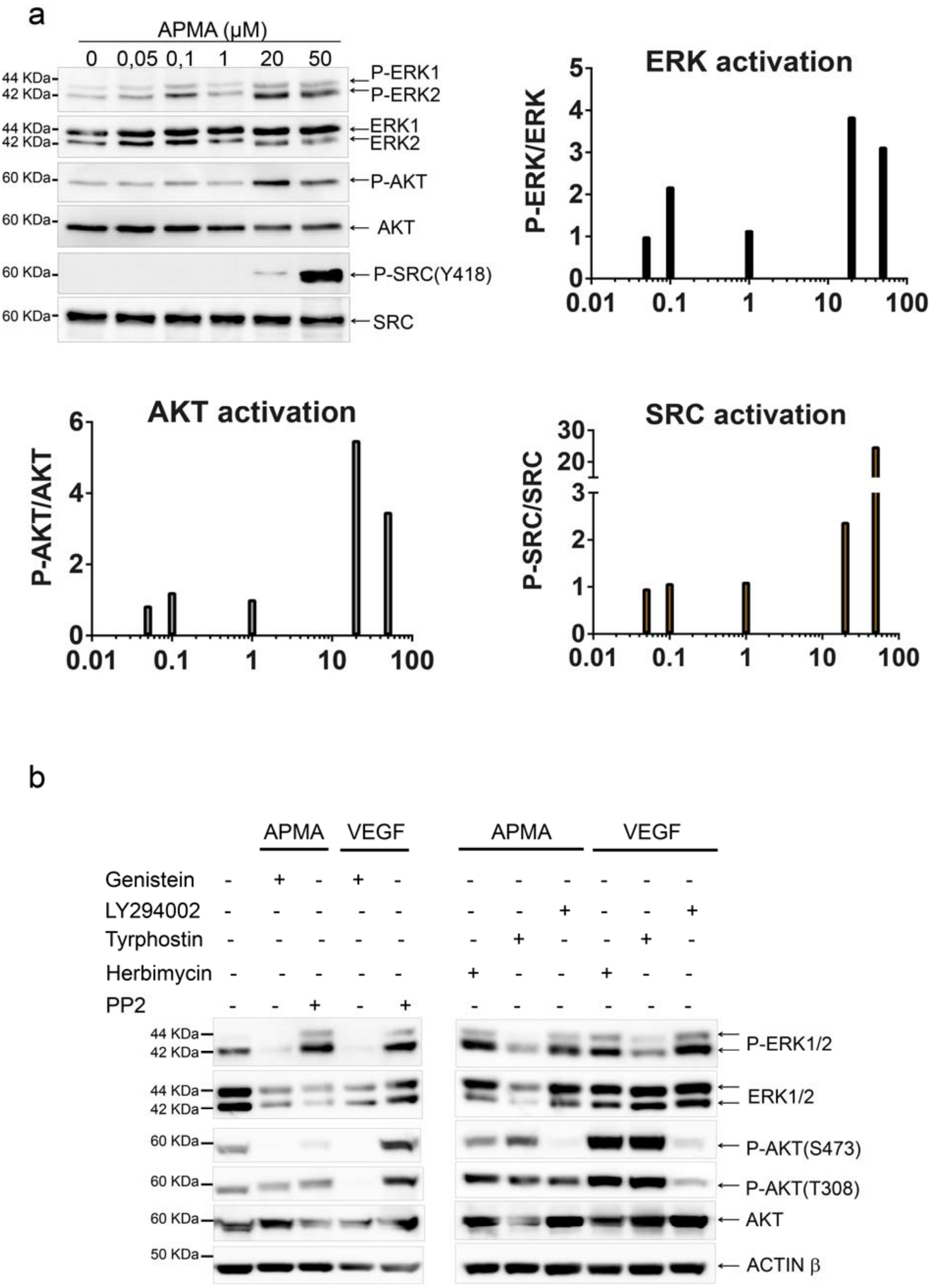

Supplementary figure 19: Activation of endothelial MMPs induces activation of MAPK, SRC and PI3K signalling pathways

a. Effect of metalloproteinase activation on signalling pathways in endothelial cells. The activation of endothelial cell-expressed MMPs with APMA (amino-phenyl mercuric acetate) is sufficient to induce dose-dependent phosphorylation of ERK1/2, AKT and SRC. b. Identification of kinases involved in

MMP-induced MAPK and AKT activations comparative to the effect of VEGF-A. Receptor tyrosine kinase inhibitor genistein inhibits both MAPK and AKT activation-induced by APMA. Epithelial growth factor receptor (EGFR) inhibitor Tyrphostin impairs ERK phosphorylation induced by APMA and VEGF-A, whereas PI3K-specific inhibitor LY294002 inhibited APMA-induced phosphorylation of AKT at S473 but not at T308. It did not affect ERK phosphorylation. The inhibitors of SRC family kinases PP2 or Herbimycin did not affect ERK and AKT activations under APMA challenges. Altogether, these results suggest the activation of receptor tyrosine kinases (RTK) after growth factor mobilization by MMP activation. Data are representative 2 independent experiments.

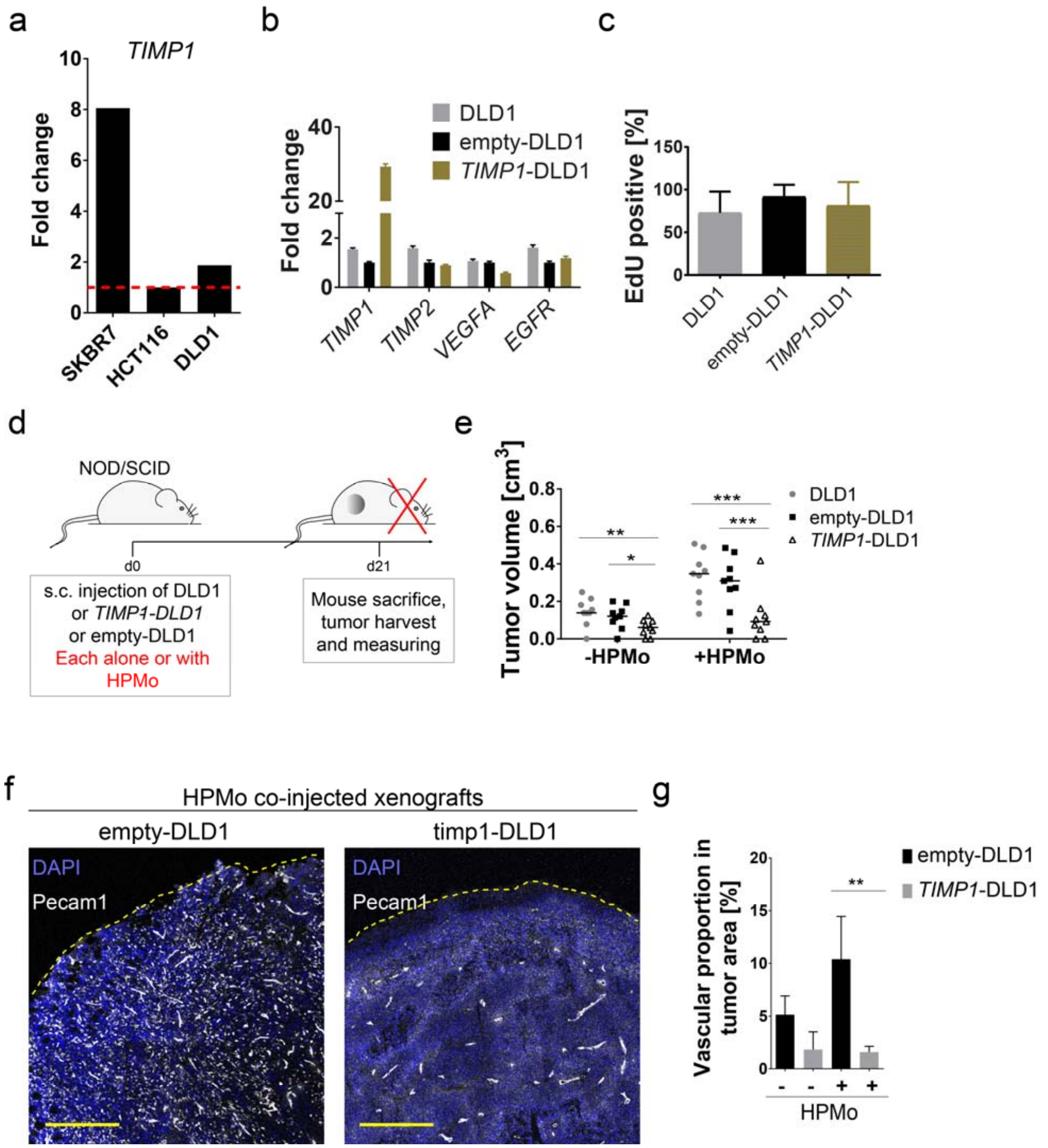

Supplementary figure 20: MMP activity mediates proangiogenic monocytes-promoting growth of DLD1 xenograft

a. Differential expression of *TIMP1* in human tumour cell lines DLD1, HCT116 and SKBR7. Tumours cells were analysed by qPCR for their expression of *TIMP1*. The *TIMP1* expression level was presented as relative to HCT116 (fold change). Data are mean of 4 technical replicate, representative of 3 independent experiments. b. Quantification of the expression of *TIMP1, TIMP2, VEGFA AND EGFR* in non-transfected DLD1, empty- or *TIMP1* vector-transfected DLD1. n=3 technical replicates in one experiment, the levels of empty-DLD1 were taken as reference for each mRNA. Data are presented as mean ± s.d. *TIMP1*-DLD1 efficiently overexpressed *TIMP1* compared to non-transfected or empty-DLD1 cells. *TIMP1* overexpression did not affect the expression levels of other genes. c. effect of TIMP1 overexpression on DLD1 proliferation potential assayed by EdU incorporation in vitro. DLD1

proliferation potential was not altered by TIMP1 overexpression. n=11 technical replicates in one experiment representative of two independent experiments, Data are presented as mean ± s.d, *TIMP1*-DLD1 proliferated at similar rate as empty-DLD1 (p=0.4965) and non-transfected DLD1 (p=0.6384). ANOVA test with Sidak adjustment was performed. d. Time course protocol to analyse the involvement of MMPs in human proangiogenic monocytes-sustained growth of DLD1 xenograft. NOD/SCID mice were subcutaneously injected with tumour cells alone (DLD1 or *TIMP1*-DLD1 or empty-DLD1) or each combined with isolated proangiogenic monocytes. e. Effect of TIMP1 overexpression on proangiogenic monocytes-sustained growth of DLD1 xenograft. TIMP1 overexpression by tumour cells alters proangiogenic monocytes-sustained growth. (n=8 xenografts per group, pooled from 2 independent experiments. f. Influence of proangiogenic monocytes co-injection on the vascular density of xenografts of DLD1 transfected either with empty vector (empty-DLD1) or *TIMP1* (*TIMP1*-DLD1). Scale bar=200 µm. g. Quantification of the influence of HPMo co-injection on the vascular density as proportion of tumour area analysed for 6 tumours per group. Data are presented as scatter dot plots with medians (horizontal lines) of tumour size for (e) and solid bars of median with maximum values as error bar for (g). *:p<0.05, **:p<0.01, ***:p<0.001 in ANOVA with Holm-Sidak adjustment for multiple comparison.

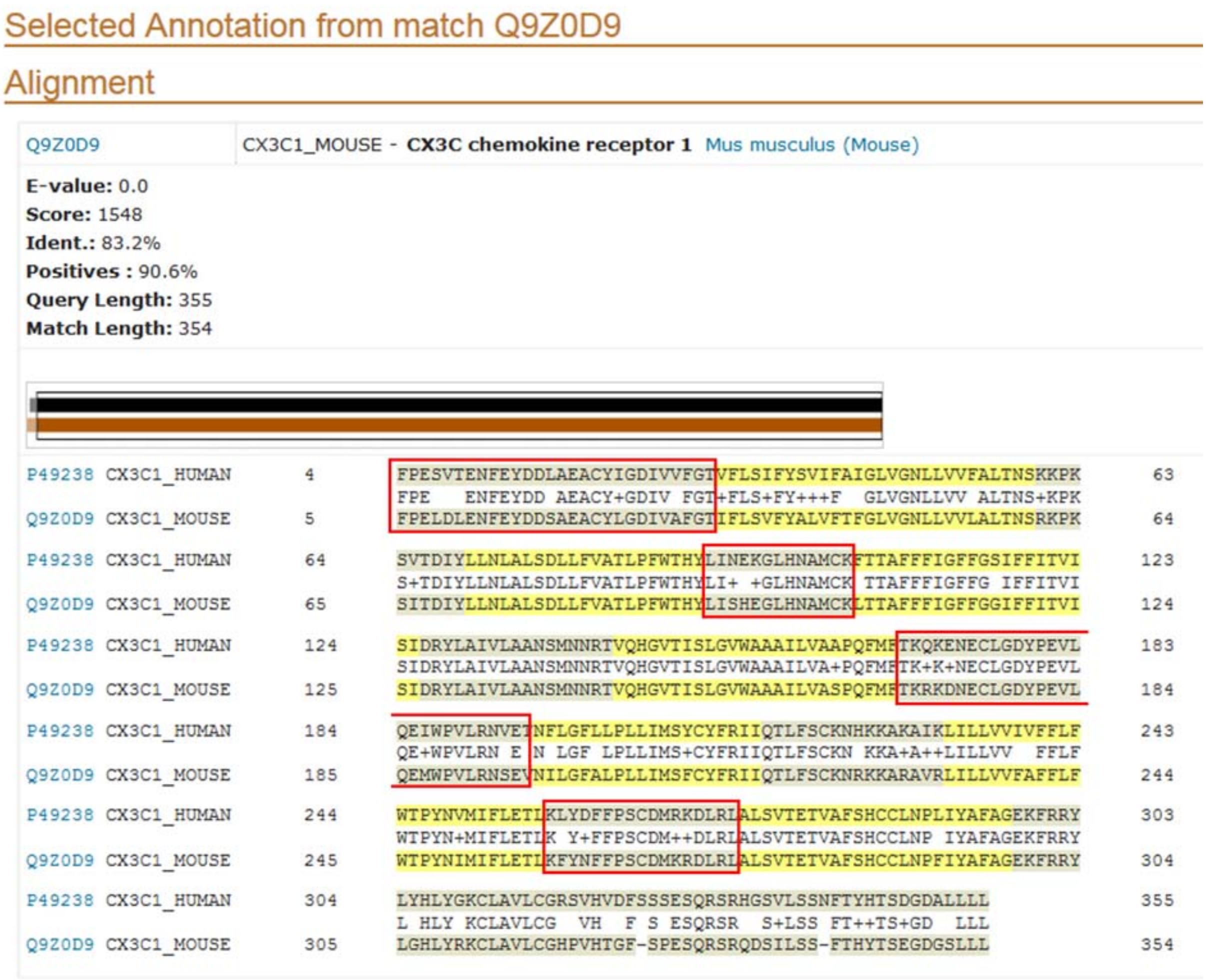

Supplementary figure 21: Alignment of human and mouse CX3CR1 amino acid sequences

The amino acid sequences of human and mouse CX3C chemokine receptor 1 were aligned with the Basic Local Alignment Search Tool (BLAST) from www.uniprot.org. The alignment showed 83.2% similarity between human and mouse CX3C receptor 1 supporting the established cross reactivity of mouse chemokine Cx3cl1 with human CX3CR1. The transmembrane sequences are underlined in yellow and extracellular portions are in red boxes.

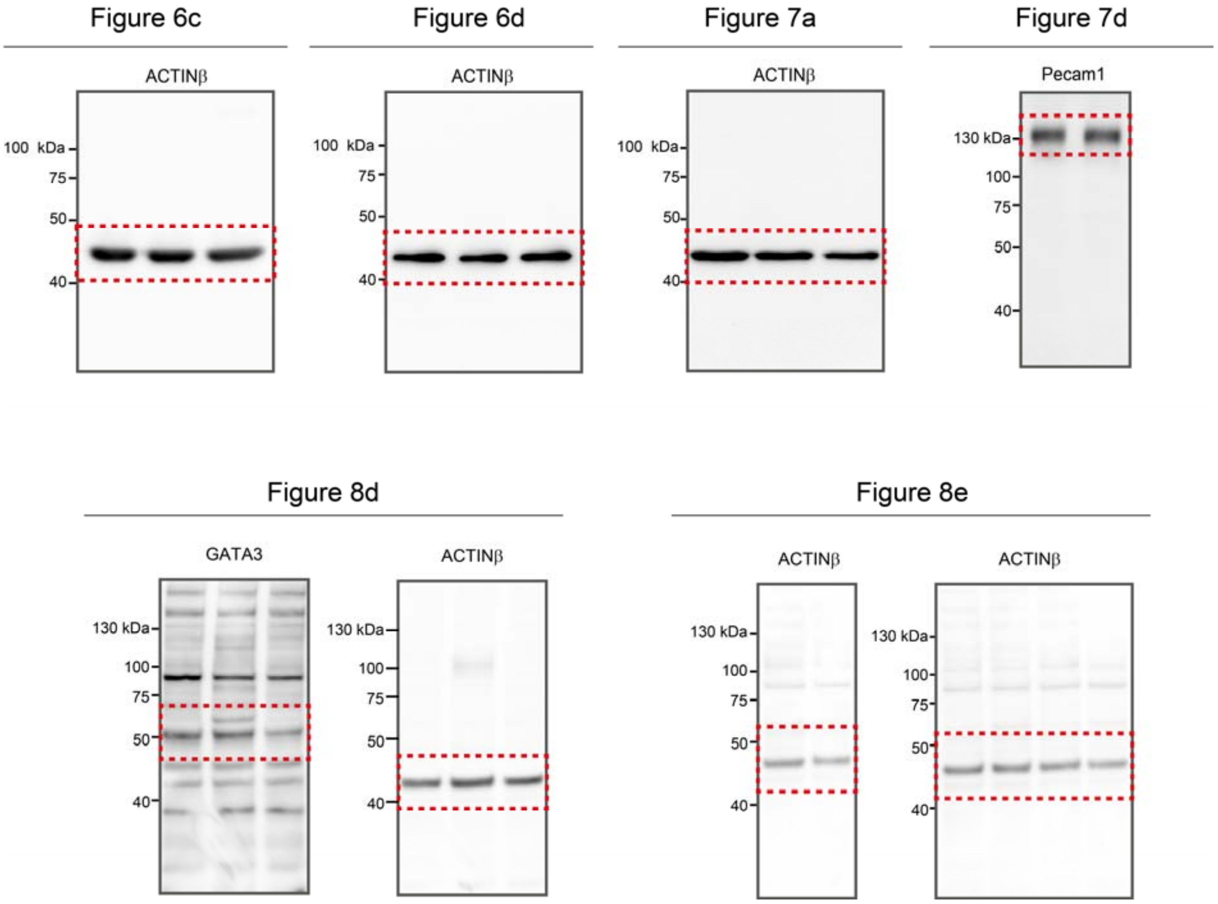

Supplementary figure 22: Full length images of blots included in main figures as portions

Dashed rectangles indicate the cropping areas presented in main figures

	TNF		TNF/VEGF-A	
	**Mean difference ± SEM**	***p-value***	**Mean difference ± SEM**	***p-value***
SELPLG	0.641669 ± 0.2011277	*0.0042*	0.025761 ± 0.2011277	*0.8992*
SELP	0.922097 ± 0.3202689	*0.0087*	0.524388 ± 0.3202689	*0.1158*
SELE	1.140353 ± 0.0917257	*<0.0001*	1.294678 ± 0.0917257	*<0.0001*
ICAM1	0.1817053 ± 0.0814856	*0.0363*	0.1543696 ± 0.0814856	*0.0714*
ICAM2	-0.145086 ± 0.1402825	*0.3123*	0.468081 ± 0.1402825	*0.0030*
CCL2	-0.678988 ± 0.1029818	*<0.0001*	-1.558228 ± 0.1029818	*<0.0001*
CXCL12	-1.476528 ± 0.2233856	*<0.0001*	0.022587 ± 0.2233856	*0.9204*
CX3CL1	**2.477763 ± 0.1038084**	*<0.0001*	**4.081778 ± 0.1038084**	*<0.0001*
CCL5	1.679716 ± 0.258347	*<0.0001*	1.440865 ± 0.258347	*<0.0001*
CCL3	-0.025387 ± 0.3138939	*0.9363*	-0.119276 ± 0.3138939	*0.7076*
ANGPT2	-2.085799 ± 0.1117464	*<0.0001*	-2.141169 ± 0.1117464	*<0.0001*
JAM1	-1.175878 ± 0.0543157	*<0.0001*	-1.192734 ± 0.0543157	*<0.0001*
JAM2	0.031106 ± 0.0169978	*0.0808*	0.029031 ± 0.0169978	*0.1017*
JAM3	-1.890425 ± 0.0994806	*<0.0001*	-2.487717 ± 0.0994806	*<0.0001*
PECAM1	-0.620564 ± 0.1042406	*<0.0001*	-0.725882 ± 0.1042406	*<0.0001*
TIE2	-1.338114 ± 0.2251464	*<0.0001*	-1.363669 ± 0.2251464	*<0.0001*
HPMo extravasation	-		+	

Supplementary table 1: Comparison of molecule expression in different inflammatory conditions.

The mRNA expression levels (dC_T) of candidate molecules were compared in HUVEC stimulated with TNF/IFNγ, TNF and TNF+VEGF-A corresponding respectively to conditions of very low, low and high transendothelial migration of proangiogenic monocytes. For the same molecule, TNF and TNF/VEGF-A were compared to TNF/IFNγ (lowest extravasation) and data are presented as mean difference of dC_T ± standard error related to TNF+IFNγ. The p-value of each comparison was shown and most of the differences were significant. The *CX3CL1* mRNA is more expressed in inflammatory conditions where proangiogenic monocytes are retained in endothelial luminal surface. CX3CL1 is then the most associated with proangiogenic monocyte retention. HPMo: proangiogenic monocytes (- indicates low extravasation and + indicates high extravasation). Result was from 3 independent experiments.

Supplementary table 2: list of antibodies

Names, -conjugation, clone	Manufacturer	Origin	Reactivity
**Blocking antibodies**			
VEGF, Bevacizumab, humanized Ab (Avastin®)	Roche	mouse	Human
Vegfr2, mAb (clone : DC101); hybridoma from	ATCC	Rat	Mouse
Rat IgG isotype control (I4131)	Sigmaaldrich	Rat	
Cx3cr1, pAb (GTX27200); dialyzed in PBS	GeneTex Inc.	Rabbit	Rat
Rabbit IgG isotype control (I5006)	Sigmaaldrich	Rabbit	
**Primary antibodies**			
CD19 (B4)-VioBlue, (clone : LT19)	Miltenyi Biotech	Mouse	Human
CD2-VioBlue, (clone: LT2)	Miltenyi Biotech	Mouse	Human
CD3-VioBlue, (clone: BW264/56)	Miltenyi Biotech	Mouse	Human
CD15-VioBlue, (clone: VIMC6)	Miltenyi Biotech	Mouse	Human
CD56-Biotin, (clone: REA196)	Miltenyi Biotech	Human	Human
CD335 (NKp46)-Biotin, (clone: 9E2)	Miltenyi Biotech	Mouse	Human
CD141 (BDCA-3)-VioBlue, (clone: AD5-14H12)	Miltenyi Biotech	Mouse	Human
CD1c (BDCA-1)-Vioblue, (clone: AD5-8E7)	Miltenyi Biotech	Mouse	Human
CD300e (IREM-2)-PE-Vio770, (clone: UP-H2)	Miltenyi Biotech	Mouse	Human
CD300e (IREM-2)-APC-Vio770, (clone: UP-H2)	Miltenyi Biotech	Mouse	Human
HLA-DR-PerCP, (clone: AC122)	Miltenyi Biotech	Mouse	Human
HLA-DR-APC-Cy7, (clone: L243)	Biolegend	Mouse	Human,
CD115(CSF-1R)-PerCP-Cy5.5, (clone:9-4D2-1E4)	Biolegend	Rat	Human
CD16-FITC, recombinant (clone: REA423)	Miltenyi Biotech	human	Human
CD16-PE, recombinant (clone: REA423)	Miltenyi Biotech	human	Human
CD16-PE, (clone: 3G8)	BD Biosciences	Mouse	Human
CD14-APC, (clone: TÜK4)	Miltenyi Biotech	Mouse	Human
CD14-FITC, (clone: M5E2)	BD Biosciences	Mouse	Human
CD45 (Ly-5)-PE-Cy7, mAb (clone: HI30)	eBioscience	Rat	Mouse
Gp38-FITC, (clone:8.1.1)	Biolegend	Syrian Hamster	Mouse
Ter119 (Ly76)-Biotin, (clone: TER-199)	eBioscience	Rat	Mouse
Ly6G-BV421, (clone: 1A8)	Biolegend	Rat	Mouse
TIE2, pAb (bs-1300R)	Bioss	Rabbit	Human
TIE2, mAb (clone: Ab33)	Biolegend	Mouse	Human
CD144 (VE-Cadherin)-FITC, (clone: REA199)	Miltenyi Biotech	Human	Human
CD144 (VE-cadherin), C19, (sc-6458)	Scbt	Goat	Human, mouse, rat, Pig
CD144 (Ve-cadherin)-APC, (clone BV13)	Biolegend	Rat	mouse
CX3CR1-APC, (clone : 2A9-1)	Miltenyi Biotech	Mouse	H
AKT (phospho-T308); mAb, (clone : C31E5E)	Cell Signaling Tech.	Rabbit	H, M, Hm, Mk
AKT (phospho-S473); mAb, (clone: D9E)	Cell Signaling Tech.	Rabbit	H, M, Hm, Mk, B, Dm,
AKT (pan); mAb, (clone : C67E7)	Cell Signaling Tech.	Rabbit	H, M, Hm, Mk, Dm

ERK1/2 (p44/42 MAPK); mAb; (clone: 137F5)	Cell Signaling Tech.	Rabbit	H, M, Hm, Mk, Dm
phospho-ERK1/2 (p44/42 MAPK); mAb; (D13.14.4E)	Cell Signaling Tech.	Rabbit	H, M, Hm, Mk, Dm
SRC (phospho-Y416), mAb (D49G4)	Cell Signaling Tech.	Rabbit	H, M, R, Mk
SRC, mAb (L4A1)	Cell Signaling Tech.	Mouse	H, M, R, Mk
CX3CL1/Fractalkine, pAb (GTX74237)	GeneTex Inc.	Rabbit	Human
CX3CL1/Fractalkine, pAb (14-7986-81)	eBioscience	Rabbit	Human, mouse, rat
CD31-PE, mAb, (clone: 390)	Biolegend	Rat	Mouse
CD31, mAb, (clone: GC51)	In house	Rat	Mouse
ACTIN β, mAb (clone: C4)	Merck Millipore	Mouse	Human
CD31 (PECAM), (clone: 9G11)	R&D	Mouse	Human
CCR1-APC, mAb (362907)	Biolegend	Mouse	Human
CCR2-APC, mAb (357207)	Biolegend	Mouse	Human
CCR5-AF647, mAb (313711)	Biolegend	Mouse	Human
CXCR4-AF647, mAb (341607)	Biolegend	Mouse	Human
**ChIP antibodies**			
NFκB p65, pAb (PA1186)	Thermo Fisher Sc.	Rabbit	Human, mouse, rat
SP1, pAb (PA19103)	Thermo Fisher Sc.	Rabbit	Human
c-Jun/AP-1, oligoclonal (711202)	Thermo Fisher Sc.	Rabbit	Human
GATA3, pAb (PA520892)	Thermo Fisher Sc.	Rabbit	Human, mouse
ERG, mAb, (clone: D-3)	Scbt	Mouse	Human
C/EBPalpha, mAb, (clone: D-5)	Scbt	Mouse	Human
H3K9Me3, pAb (491008)	Thermo Fisher Sc.	Rabbit	H, m, rt
Rabbit IgG control (from Magnify kit)	Thermo Fisher Sc.		
Mouse IgG control (from Magnify kit)	Thermo Fisher Sc.		
**Secondary antibodies or streptavidin**			
HRP- anti-goat IgG (H+L), min crossreactivity	Jackson Im. Res Lab	Donkey	Goat
HRP- anti-rabbit IgG (H+L), min crossreactivity	Jackson Im. Res Lab	Goat	Rabbit
HRP- anti-mouse IgG (H+L), min crossreactivity	Jackson Im. Res Lab	Donkey	Mouse
DyLight®649- anti-goat IgG (H+L),	Jackson Im. Res Lab	Donkey	Goat
DyLight®594- anti-goat IgG (H+L),	Jackson Im. Res Lab	Donkey	Goat
Alexa Fluor®594- anti-Mouse IgG (H+L),	Jackson Im. Res Lab	Donkey	Mouse
Alexa Fluor®488- anti-Mouse IgG (H+L),	Jackson Im. Res Lab	Donkey	Mouse
DyLight®594- anti-rabbit IgG (H+L),	Jackson Im. Res Lab	Donkey	Rabbit
DyLight®650- anti-rabbit IgG (H+L)	Thermo Fisher Sc.	Donkey	Rabbit
DyLight®405-Strepavidin	Jackson Im. Res Lab	*Strep. avidinii*	Biotin
APC-Cy7-strepavidin	Biolegend	*Strep.*	Biotin

		avidinii	
PerCP-Cy5.5-streptavidin	eBioscience	*Strep. avidinii*	Biotin

Supplementary table 3: list of primers for qPCR

Name	Forward	Reverse	size	PrimerBank ID
TIE2	GCCCAAGCCTTCCAAAACAG	TTGCCCTCCCCAATCACATC	155	
TIE2	CTGAAAATGCTGACCGGGAC	GGCACTCAAGCCCTATCCAT	135	
ANGPT1	GAGCAAGTTTTGCGAGAGGC	TGAGTCAGAATGGCAGCGAG	92	
ANGPT2	CTCGAATACGATGACTCGGTG	TCATTAGCCACTGAGTGTTGTTT	76	169646744c2
VEGFA	AGGGCAGAATCATCACGAAGT	AGGGTCTCGATTGGATGGCA	75	284172466c1
FGF2	AGAAGAGCGACCCTCACATCA	CGGTTAGCACACACTCCTTTG	82	153285460c1
CX3CL1	CGCGCAATCATCTTGGAGAC	CATCGCGTCCTTGACCCAT	78	*54111253c2*
CX3CL1	GCCACAGGCGAAAGCAGTA	GGAGGCACTCGGAAAAGCTC	179	54111253c3
CX3CL1	TATCTCTGTCGTGGCTGCTC	TCCAAGATGATTGCGCGTT	187	
SELP	ATGGGTGGGAACCAAAAAGG	GGCTGACGGACTCTTGATGTAT	118	157419153c2
SELE	CAGCAAAGGTACACACACCTG	CAGACCCACACATTGTTGACTT	128	187960041c2
SELPLG	CCTGAGTCTACCACTGTGGAG	GCTGCTGAATCCGTGGACA	122	331284235c2
ICAM1	GTATGAACTGAGCAATGTGCAAG	GTTCCACCCGTTCTGGAGTC	119	167466197c3
ICAM2	CGGATGAGAAGGTATTCGAGGT	CACCCACTTCAGGCTGGTTAC	111	153082685c1
CCL3	AGTTCTCTGCATCACTTGCTG	CGGCTTCGCTTGGTTAGGAA	*151*	4506843a1
CCL5	CCAGCAGTCGTCTTTGTCAC	CTCTGGGTTGGCACACACTT	54	22538813c1
CXCL12	ATTCTCAACACTCCAAACTGTGC	ACTTTAGCTTCGGGTCAATGC	88	296011022c1
CCL2	CAGCCAGATGCAATCAATGCC	TGGAATCCTGAACCCACTTCT	190	4506841a1
JAM1	ACCTTCTTGCCAACTGGTATCA	AGCACGATGAGCTTGACCTTG	125	164419740c3
CCL4	CTCCCAGCCAGCTGTGGTATTC	CCAGGATTCACTGGGATCAGC	70	
CCL13	CTCAACGTCCCATCTACTTGC	TCTTCAGGGTGTGAGCTTTCC	211	22538799b1
CCL14	CCAAGCCCGGAATTGTCTTCA	GGGTTGGTACAGACGGAATGG	55	221316558c1
CCL23	CATCTCCTACACCCCACGAAG	GGGTTGGCACAGAAACGTC	126	22538807c1
JAM2	GCAGTAGAGTACCAAGAGGCT	AGACACTCCGACCCAGTTTCT	94	21704284c1
JAM3	CTGCTGTTCACAAGGACGAC	CGCCAATGTTCAGGTCATAG	111	
PECAM1	AACAGTGTTGACATGAAGAGCC	TGTAAAACAGCACGTCATCCTT	148	313760623c1
ACTB	GCAAAGACCTGTACGCCAAC	CTAGAAGCATTTGCGGTGGA	260	
GAPDH	AGAAGGCTGGGGCTCATTTG	AGGGGCCATCCACAGTCTTC	258	
TNF	GAGGCCAAGCCCTGGTATG	CGGGCCGATTGATCTCAGC	91	25952110c2
IFNG	TCGGTAACTGACTTGAATGTCCA	TCGCTTCCCTGTTTTAGCTGC	93	56786137c1
IL1B	TTCGACACATGGGATAACGAGG	TTTTTGCTGTGAGTCCCGGAG	84	27894305c3
IL4	AAACGGCTCGACAGGAACCT	CTCTGGTTGGCTTCCTTCACA	71	
IL13	GAGGATGCTGAGCGGATTCTG	CACCTCGATTTTGGTGTCTCG	85	26787977c3
IL6	CCTGAACCTTCCAAAGATGGC	TTCACCAGGCAAGTCTCCTCA	75	224831235c2
TIMP1	CTTCTGCAATTCCGACCTCGT	ACGCTGGTATAAGGTGGTCTG	79	73858576c1
TIMP2	GCTGCGAGTGCAAGATCAC	TGGTGCCCGTTGATGTTCTTC	109	73858577c2
EGFR	TTGCCGCAAAGTGTGTAACG	GTCACCCCTAAATGCCACCG	142	41327735c3

Supplementary table 4: list of primers for ChIP analysis by qPCR

**Transcription factor or histone**	***Analyzed gene***	**Forward**	**Reverse**	**Size**
SP1	*human CX3CL1*	CATCCTGAGGAATCCAGCGG	TATAATGGGGTTGGCTGCCT	100
SP1	*human KDR*	CACACATTGACCGCTCTCCC	GCTCTAGAGTTTCGGCACCA	71
NFκB	*human CX3CL1*	ATGGACGAGTCTGTGGTCCT	GCTTCCTGGGAAGAAGGAGT	80
NFκB	*human ICAM1*	AGTCCAGGGCTGCTTCTACT	GCGTCCCACTTCTCAGCTTT	74
AP-1	*human CX3CL1*	AACCTGCAGCTACGAGTCAG	GAGTGGGAGTGAATGCGTCA	70
AP-1	*human CCL2*	GAGTTATCATGGACCATCCAAGCA	CCCTTGTCCTTTTCTGAGTCAC	93
GATA3	*human CX3CL1*	GGGATCTCCTTCTCCCTCCT	CCAGGAAACCAAAGCAGACA	90
GATA3	*human VWF*	ACCATAGAACAGGGCCCATC	TCGGACTCCCGGGATAACTAA	95
C/EBPα	*human CX3CL1*	AGGGGCACCGTATTTTCTGG	CAGGCCCTATTGAGTGGAGC	89
C/EBPα	*human SYK*	AGTTCAAAGCATTGCAGCGT	CCAAGAATCAGATATGGCACAACA	71
ERG-1 and ERG-2	*human cx3cl1*	ACTCCAAGAGCTGACGCATT	GGCGGGTAGGCTATAGGAGC	93
ERG-2 and ERG-3	*human CX3CL1*	CATCTGGCAGGGTGACCTTC	TCTGGACAGTGGGGACTACT	78
ERG-1 and ERG-2	*human JUNB*	GGGACAGTACTTTTACCCCCG	CCTCCTGCTCCTCGGTGA	83
ERG-2 and ERG-3	*human VIM*	CTCTGGTTCAGTCCCAGGC	GGGGCTTTCCGGATGATAGT	100
H3K9Me3	*human CX3CL1*	CATCCTGAGGAATCCAGCGG	TATAATGGGGTTGGCTGCCT	100
H3K9Me3	*human SAT2*	CTGCAATCATCCAATGGTCG	GATTCCATTCGGGTCCATTC	178
Gata3	*mouse Cx3cl1*	GCAGCCAGGCTCCTAAGATAG	CGGGGACAGGAGTGATAAGC	84
Nfκb	*mouse Cx3cl1*	AACTTCCGAGGCACAGGATG	AGATGTCAGCCGCCTCAAAA	74

Supplementary table 5: list of cytokines and concentrations of use

Factor name	Concentration of use	Origin	Manufacturer
TNF	500 U/ml	Human	Peprotech
Tnf	10^6 U/kg	Mouse	Peprotech
IFNγ	200 U/ml	Human	Peprotech
IFNγ, research grade	200 U/ml	Human	Miltenyi Biotech
IL-1β	200 U/ml	Human	Peprotech
IL-4, research grade	200 U/ml	Human	Miltenyi Biotech
IL-13, research grade	200 U/ml	Human	Miltenyi Biotech
CSF-1	0.5 mg/kg	Human	Peprotech
VEGF-A	1 µg/ml	Human	Peprotech
Vegf-a	0.2 mg/kg	Mouse	Peprotech
FGF2	1 µg/ml	Human	Peprotech
Fgf2	0.5 mg/kg	Mouse	Peprotech
ANGPT2	1 µg/ml	Human	Peprotech

Supplementary Table 6: list of chemokines and concentration of use

Chemokine	Concentration of use	Origin	Manufacturer
CX3CL1	50 ng/ml	Human	Peprotech
CXCL12	50 ng/ml	Human	Peprotech
CCL2	50 ng/ml	Human	Peprotech
CCL3	50 ng/ml	Human	Peprotech
CCL5	50 ng/ml	Human	Peprotech
CCL13	50 ng/ml	Human	Peprotech
CCL14	50 ng/ml	Human	Peprotech
CCL23	50 ng/ml	Human	Peprotech

Supplementary Table 7: list of chemical inhibitors used in this study

Inhibitor name	Concentration of use	Target	Manufacturer
Genistein	100 µM	Tyrosine kinases	EMD Millipore
LY294002	50 µM	PI3K	EMD Millipore
PP2	10 µM	SFK	EMD Millipore
Tyrphostin 47	50 µM	EGFR kinase	EMD Millipore
Herbimycin A	10 µM	SFK	EMD Millipore
Bevacizumab	50 µg/ml	Soluble VEGF-A	Roche
GM6001	10 µM	MMPs	SigmaAldrich

Supplementary Note 1: Human non-classical monocytes express TIE2.

The expression of TIE2 is a hallmark of angiogenic phagocytes in mouse and human[1,2]. Using flow cytometry, we defined pan-monocytes in peripheral blood as $CD19^-CD3^-HLA\text{-}DR^+CD300e^+CD56^-CD115^+CD141^-$ (Supplementary figure 1a). Morphological analysis showed that human TIE2+ monocytes present variable cell diameter having low granularity compared to TIE2- cells presenting higher granularity with bigger and uniform cell sizes (Supplementary figure 1b). We compared human monocyte populations (pop) including inflammatory $CD14^+CD16^-$ (pop1), intermediate $CD14^+CD16^+$ (pop2) and non-classical or patrolling $CD14^{dim}CD16^+$ (pop3) cells for TIE2 expression (Supplementary figure 1c). The non-classical $CD14^{dim}CD16^+$ monocytes highly expressed TIE2 (MFI=1670), the intermediate monocytes expressed lower levels (MFI=491) whereas inflammatory monocytes were negative (MFI=235). Analysis of TIE2 mRNA levels by real time quantitative polymerase chain reaction (qPCR) of the three subpopulations correlated with the differences observed at protein level (Supplementary figure 1d). These results indicate that Tie2 expression is a novel marker for human non-classical $CD14^{dim}CD16^+$ monocytes. Previously it was found that non-sorted human intermediate and non-classical monocytes were angiogenic, we therefore tested whether this was due to the non-classical monocytes in this $CD16^+$ pool of cells.

Supplementary Note 2: Tube formation assay to quantify angiogenesis in vitro

Endothelial cells in proangiogenic conditions on 3D matrix spontaneously sprout, migrate, interconnect and form tube-like structures[3,4]. As exemplified in Supplementary figure 2a, fetal calf serum (FCS) induces endothelial cell sprouting and tube formation whereas simple culture medium alone fails to trigger tube formation. Different parameters of the endothelial network can be measured with ImageJ Angiogenesis Analyzer toolset[5] (Supplementary figure 2b). Angiogenic capacity can be reliably quantified with the total tube length, which is the sum of all segments and branches (Supplementary figure 2c). The tubing integrity (network stability index) is the ratio of tube length to the number of isolated segments, and is another measurement that illustrates the network unity and integrity. As illustrated in Supplementary figure 2d, these two measurement quantify the proangiogenic potential of FCS and demonstrates how it can reliably induce endothelial tube formation, when compared to simple medium. We therefore employed these assays to quantify the capacity of sorted monocyte subsets to induce formation of endothelial tube-like structures as an index of their proangiogenic potential.

Supplementary Note 3: Human non-classical monocytes are *bona fide* proangiogenic cells that promote growth of human cancer xenografts in NOD/SCID mice.

Monocyte subsets were stained with green cell tracker CMFDA and co-cultured with human umbilical vein endothelial cells (HUVEC) on matrigel and imaged by time-lapse microscopy combining phase contrast and fluorescence (Supplementary figure 3a). Inflammatory monocytes (pop1) showed lower index of endothelial tube formation and integrity, compared to intermediate (pop2) and non-classical monocytes (pop3). We also used 5-ethynyl-2'-deoxyuridine (EdU) incorporation into HUVEC to measure the mitogenic activity of monocyte supernatants. Non-classical and intermediate monocytes were two- to three-fold more mitogenic for endothelial cells compared to inflammatory monocytes (Supplementary figure 3b). This demonstrates that non-classical and intermediate monocytes have the capacity to promote angiogenesis. We then refer to CD16+ monocytic pool collectively as human proangiogenic monocytes (HPMo) and to CD14+CD16- inflammatory cells as human non-angiogenic monocytes (HNMo). We tested the effect of HPMo and HNMo on the development of human colorectal (DLD1 and HCT116) and breast (SKBR7) tumours after subcutaneous (s.c) co-injection in NOD/SCID mice (Supplementary figure 4a). Concordant with their angiogenic capacity, HPMo boosted DLD1 and HCT116 growth compared to tumour cells injected alone or with HNMo (Supplementary figure 4b). The growth of breast tumour SKBR7 xenografts was

not affected by any of the monocyte subpopulations co-injected. This indicates a tumour-dependent influence of proangiogenic monocytes. Tumour vascular density was analysis after cross section and immunofluorescence staining of pieces of xenografts with antibody against mouse Pecam1 and the nuclei with DAPI (Supplementary figure 4c,d). HPMo co-injection with DLD1 induced an increased vascular density compared to DLD1 alone. Concordant with an absence of effect on SKBR7 xenografts, HPMo co-injection with this tumour cells did not change the vascular density in SKBR7 xenografts. These findings have demonstrated that the $CD16^+$ pool (non-classical and intermediate) of monocytes are proangiogenic and have the capacity to boost human tumour xenograft development *in vivo.*

Supplementary Note 4: Live imaging of human monocyte transmigration under flow

Throughout our study, we used a combination of live phase-contract imaging and fluorescence microscopy of human monocyte recruitment to inflamed endothelial cell monolayer as previously described[6-8]. This method takes advantage of the physical principles of phase-contrast imaging allowing structure discrimination according to their refractive indices. We can clearly differentiate between white captured monocytes, grey body with brightened edges of crawling non-transmigrated monocytes and completely dark body and flat shape of transmigrated monocytes (Supplementary Figure 6a). The video sequence demonstrates the shift in phase and shape changing that occurs with individual monocytes during the course of recruitment, which allows the quantification of the sequential steps that define the recruitment process. Co-culturing monocytes under flow with HUVECs stained for VE-cadherin using a non-blocking antibody, clearly shows over time monocyte capture (phase white), luminal crawling toward endothelial cell junctions (phase grey with white edges), and transmigration where they appear dark gray and flat (Supplementary figure 6b and Movie 1). We used this time-lapse imaging method that allow recording of the migratory history of each individual monocytes (Supplementary Movie 2). We compared monocyte recruitment to inflamed or non-inflamed endothelium under flow and static conditions. Under static conditions, up to 30% of monocyte adhesion and transmigration were independent of endothelial cell inflammation status (Supplementary figure 6c-d). Conversely under flow; a condition more relevant to blood circulation in post-capillary venules, monocyte adhesion and transmigration were reliant on endothelial cell activation. These results demonstrated that applying monocytes under flow is required to breakdown the step-by-step combinatorial process that defines recruitment, with the described technique providing a robust method to explore the mechanisms that define human angiogenic monocyte recruitment.

Supplementary Note 5: homing of proangiogenic monocytes in non-tumoral inflammation

We investigated whether angiogenic factors would induce angiogenic factors-driven inflammation (ADIn) when supplemented in a peritonitis model. NOD/SCID mice were i.p injected with thioglycolate and either Tnf alone (conventional inflammation), or Tnf plus combined angiogenic factors including Vegfa and Fgf2 (ADIn). Mice were subcutaneously injected with 0.5 mg/kg of human CSF-1[9]. HPMo extravasation was analysed after adoptive transfer of human pan-monocytes (Supplementary figure 11a). The number of recruited human proangiogenic CD16+ monocytes was low in conventional inflammation but increased substantially in ADIn (mean difference of proportion = 0.24; 95%CI [0.14; 0.34], p=0.001) (Supplementary figure 11b-d). The recruitment of human inflammatory monocytes appeared similar in both inflammatory conditions (Supplementary figure 11b,e). Furthermore, non-classical CD16+ monocytes have the capacity to extravasate into non-tumour environments if ADIn stromal cues are present, describing a potential recruitment mechanism for these cells in chronic inflammation.

Additional references

1 De Palma, M., Venneri, M. A., Roca, C. & Naldini, L. Targeting exogenous genes to tumor angiogenesis by transplantation of genetically modified hematopoietic stem cells. *Nature medicine* **9**, 789-795, doi:10.1038/nm871 (2003).

2 De Palma, M. *et al.* Tie2 identifies a hematopoietic lineage of proangiogenic monocytes required for tumor vessel formation and a mesenchymal population of pericyte progenitors. *Cancer Cell* **8**, 211-226, doi:10.1016/j.ccr.2005.08.002 (2005).

3 Montesano, R., Orci, L. & Vassalli, P. In vitro rapid organization of endothelial cells into capillary-like networks is promoted by collagen matrices. *J Cell Biol* **97**, 1648-1652 (1983).

4 Yang, S., Graham, J., Kahn, J. W., Schwartz, E. A. & Gerritsen, M. E. Functional roles for PECAM-1 (CD31) and VE-cadherin (CD144) in tube assembly and lumen formation in three-dimensional collagen gels. *Am J Pathol* **155**, 887-895, doi:10.1016/S0002-9440(10)65188-7 (1999).

5 Carpentier, G. Contribution: Angiogenesis Analyzer. *ImageJ News* (2012).

6 Luu, N. T., Rainger, G. E. & Nash, G. B. Kinetics of the different steps during neutrophil migration through cultured endothelial monolayers treated with tumour necrosis factor-alpha. *J Vasc Res* **36**, 477-485, doi:25690 (1999).

7 Schenkel, A. R., Mamdouh, Z. & Muller, W. A. Locomotion of monocytes on endothelium is a critical step during extravasation. *Nat Immunol* **5**, 393-400, doi:10.1038/ni1051 (2004).

8 Bradfield, P. F. *et al.* JAM-C regulates unidirectional monocyte transendothelial migration in inflammation. *Blood* **110**, 2545-2555, doi:10.1182/blood-2007-03-078733 (2007).

9 Qian, B. Z. *et al.* CCL2 recruits inflammatory monocytes to facilitate breast-tumour metastasis. *Nature* **475**, 222-U129, doi:10.1038/nature10138 (2011).
