# Supplementary material for: Angiogenic factor-driven inflammation promotes extravasation of human proangiogenic monocytes to tumours
Source: Nat Commun. 2018 Jan 24;9:355. doi: 10.1038/s41467-017-02610-0 (PMC5783934; doi:10.1038/s41467-017-02610-0)
Supplement: Supplementary file 2 — Description of Additional Supplementary Files [file 41467_2017_2610_MOESM2_ESM.pdf]

**Description of Additional Supplementary Files**

File Name: Supplementary Movie 1
Description: Monocyte transendothelial migration through paracellular route under flow.

Activated endothelial cell junctions were stained with a fluorescently-labeled, non-blocking anti-VE- cadherin antibody (Green) to monitor the site of monocyte transmigration. Combining fluorescent and phase-contrast microscopy allowed live-imaging of the site of monocyte transmigration. Monocytes exclusively transmigrate through cell junction under flow.

File Name: Supplementary Movie 2
Description: Tracking of monocytes from capture to transendothelial migration.

To quantify the initial steps of monocyte recruitment from capture to transmigration, individual cells were tracked and their migration analyzed. Tracks of monocytes before and after transmigration are shown in red and yellow respectively.

File Name: Supplementary Movie 3
Description: Proangiogenic monocytes continuously crawl in conventional inflammation.

Zoomed video showing capture and continuous crawling of non-classical proangiogenic monocytes (CD16+) without transendothelial migration whereas inflammatory monocytes (CD16-) transmigrate. This constitutes the representative migratory behavior of non-classical monocyte in acute inflammation.

File Name: Supplementary Movie 4
Description: Angiogenic factors-driven inflammation triggers their transmigration

Zoomed video showing capture and rapid transmigration of patrolling monocytes (CD16+) at the same rate than inflammatory monocytes.
